# An efficient mid-infrared computational spectrometer based on synergistic microcavity-coupled photonic crystal waveguides

**DOI:** 10.1038/s41467-026-73934-z

**Published:** 2026-06-03

**Authors:** Lipeng Xia, Yuhan Sun, Jiahua Jiang, Hong Zhang, Weixiong Huang, Yuheng Liu, Chang Chang, Yixiang Zhang, Chaofeng Ye, Yiming Ma, Xiaochuan Xu, Chengkuo Lee, Yi Zou

**Affiliations:** 1 School of Information Science and Technology, ShanghaiTech University, Shanghai, China; 2 Shanghai Institute of Microsystem and Information Technology, Chinese Academy of Sciences, Shanghai, China; 3 University of Chinese Academy of Sciences, Beijing, China; 4 Department of Mathematics, University of Birmingham, Birmingham, UK; 5 School of Microelectronics, Shanghai University, Shanghai, China; 6 State Key Laboratory on Tunable Laser Technology, Harbin Institute of Technology, Xili University Town, Harbin Institute of Technology campus, Shenzhen Guangdong, China; 7 Department of Electrical and Computer Engineering, National University of Singapore, Singapore, Singapore

**Keywords:** Mid-infrared photonics, Infrared spectroscopy

## Abstract

The mid-infrared spectral region holds substantial promise for telecommunications, chemical sensing, and biomedical diagnostics applications. These fields increasingly demand high-resolution, miniaturized, and portable spectrometers. Leveraging recent advances in silicon photonics and computational reconstruction techniques, on-chip spectrometers offer a compact and lightweight solution. This paper presents a distinctive photonic spectrometer prototype based on microcavity-coupled photonic crystal waveguide (MPCW) architecture, operating at the mid-infrared region. The device achieves uniquely defined spectral responses by synergistically integrating non-uniform microcavity resonances with sharp photonics crystal band edges. Benefiting from the slow-light effect, titanium microheaters provide efficient thermal tuning, facilitating further precise spectral response control. The alternating optimization approach for spectral reconstruction eliminates the need for exhaustive parameter searches and substantially alleviates the computational burden. Thus, our MPCW spectrometer can retrieve an unknown spectrum with a resolution of 0.5 nm over a 100 nm bandwidth within several seconds, demonstrating high performance and robustness. Furthermore, the scalable nature of photonic crystal designs permits straightforward adaptation to other wavelength regimes by tailoring the MPCW dimensions.

## Abstract (web summary)

Researchers developed a compact silicon mid-infrared spectrometer using photonic crystal waveguides. This tiny chip achieves high-resolution, fast reconstruction, providing a scalable and efficient platform for integrated spectral analysis.

## Introduction

Infrared spectroscopy is significant in multiple fields, including atmospheric research, pharmaceutical manufacturing, environmental monitoring, and disease diagnostics^1–8^. At the core of infrared spectroscopy lies the optical spectrometer, an essential instrument for spectral measurement. Traditional bulk spectrometers, which often rely on diffractive or interferometric elements, are mature, stable, and widely used in commercial applications. However, their bulky and fragile nature, along with high costs, make them unsuitable for emerging use cases like the Internet of Things (IoT) and wearable technologies^9,10^. As such, reducing the size, weight, and power consumption (SWaP) of spectrometers, without sacrificing performance, has become a critical development focus.

Recent advancements in CMOS fabrication have accelerated the growth of silicon photonics^8^. As part of this evolving ecosystem, on-chip optical spectrometers present a promising alternative for spectral measurement^11^. Early efforts sought to replicate bulk spectrometers using integrated photonic structures^10,12^. Diffractive-type integrated spectrometers are commonly employed, such as planar concave gratings, arrayed waveguide gratings, and meta-lenses^13–15^. However, in these systems, higher spectral resolution requires longer optical paths, which are constrained by the chip footprint, typically on the centimeter scale^10^. Fourier-transform spectrometers using passive interferometric architectures, such as Mach-Zehnder interferometer (MZI) arrays, require multiple interferometers to achieve a broad bandwidth and higher resolution. Their resolution depends on the maximum optical path difference (OPD), while bandwidth is limited by the number of interferometers, leading to significant spatial consumption^16–19^. Modulation-based interferometric spectrometers improve OPD but often demand high power levels and are sensitive to temperature variations, which can degrade spectral accuracy and coupling efficiency, necessitating complex compensation mechanisms^20,21^. To balance high resolution and compact size, narrowband filters such as micro-ring resonators and Fabry-Pérot (F-P) cavities have been employed. Micro-ring resonators are well-known for their high-quality factors (Q), essential for many applications. However, they often require post-fabrication tuning to align with the desired wavelengths precisely. Moreover, their bandwidth is limited by the free spectral range (FSR), and additional demultiplexing components are often needed to fully exploit their capabilities^22^. In contrast, Fabry-Pérot (F-P) type filters offer smaller footprints that enable larger FSR. However, they generally exhibit lower Q-factors^23–25^. Recently, computational spectrometers have emerged as a powerful solution, offering both high resolution and broad bandwidth through spectral response engineering and signal reconstruction techniques^10,26^. These devices use pre-calibrated, broadband-encoded spectral filters, such as waveguide filters, cascaded MZIs, and quantum dot arrays, and reconstruct the input spectrum computationally^27–31^. This approach has shown excellent potential for compact and high-performance spectroscopic applications.

However, most reported on-chip spectrometers operate in the near-infrared region, with relatively limited exploration into longer wavelengths^18,32,33^. The mid-infrared (MIR) waveband offers distinct advantages, including significantly enhanced absorption from molecular vibrations that are orders of magnitude stronger than those in the telecom band. Additionally, silicon dioxide (SiO_2_) remains transparent around 2 μm, allowing compatibility with silicon-on-insulator (SOI) platforms and fiber-based systems^5,34^. These properties make the 2 μm MIR particularly valuable for gas sensing, environmental monitoring, and biomedical diagnostics applications, underscoring the need for compact spectrometers operating in this spectral range.

**Fig. 1:**
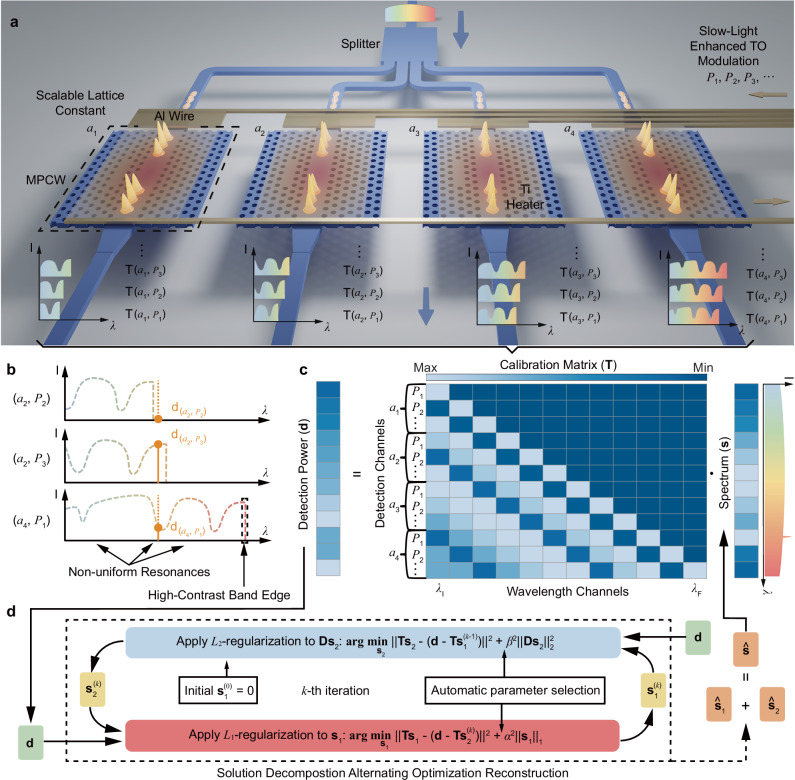
Visualization of our MPCW Spectrometer and its sampling and reconstruction process. **a** Working principle of the on-chip MPCW-based spectrometer. Blue regions indicate the silicon device layer, while semi-transparent and brown overlays correspond to Ti microheaters and Al wires, respectively. The MPCW unit consists of an L21 microcavity coupled PCW with a group index taper. Each MPCW has a unique lattice constant (*a*), corresponding to a spectral response with a specific band edge wavelength and distinct resonances. With the aid of the slow-light enhanced TO modulation, the band edge wavelength and resonance of the response can be finely tuned by applying different thermal power ($P$). Each unique sampling step (${a}_{i},\,{P}_{j}$) possesses a specific spectral response ${{{{\rm{T}}}}}_{\left({a}_{i},\,{P}_{j}\right)}$. I: intensity, $\lambda$: wavelength. **b** The response spectrum of the MPCW, featuring a high-contrast band edge (enabled by group index taper) and non-uniform resonances (enabled by L21 Microcavity). From the top and middle panels, a specific wavelength is first sampled by the high-contrast band edge from step $({a}_{2},\,{P}_{2})$ to $({a}_{2},\,{P}_{3})$, and then sampled by the resonance in step, like $\left({a}_{4},\,{P}_{1}\right)$ in the bottom panel. Each sampling step $({a}_{i},\,{P}_{j})$ outputs the corresponding detection power ${{{{\rm{d}}}}}_{\left({a}_{i},{P}_{j}\right)}$. **c** Illustration of the matrix form spectral sampling process: the continuous input spectrum can be described as a discretized vector (${{{\bf{s}}}}$), sampled by the spectrometer, and converted into a detection vector (${{{\bf{d}}}}$) based on power readings from the sampling step $({a}_{i},\,{P}_{j})$. **d** The computational spectral reconstruction process using the solution decomposition alternating optimization method. This method works by dividing the entire problem into two subproblems (${L}_{1}$ and ${L}_{2}$), solving them alternately with automatic hyperparameter ($\alpha$ and $\beta$) selection, and estimating the unknown input spectrum efficiently.

In this paper, we present a computational spectrometer based on microcavity-coupled photonic crystal waveguides (MPCWs). It comprises eight MPCWs integrated with titanium (Ti) thermo-optic (TO) heaters, each measuring approximately $7\times 30$ μm^2^. Figure 1a depicts a group of the MPCW units. The MPCW unit consists of an L21 microcavity side-coupled to a W1 photonic crystal waveguide (PCW) incorporating a group index engineered taper. This taper reduces Fresnel reflection and sharpens the band edge, functioning as an effective band-edge filter. Furthermore, variations in group index across the PCW transmission band enable microcavity-induced resonances with a non-uniform FSR over a broad wavelength range. As shown in Fig. 1b, the sharp band edges in response facilitate high-resolution sampling, while the non-uniform resonance helps overcome the limitations of uniform FSR in sampling broad-range spectra. In our spectrometer, each MPCW unit has a unique lattice constant $a$ and can be modulated by the heater with different modulation power $P$, contributing to distinct bandgap positions and fine control of the band edge, respectively, thereby increasing sampling density. These sampling processes can be described in matrix form, shown as Fig. 1c. It is worth noting that our MPCW exhibits a slow-light effect near the photonic bandgap, which facilitates efficient heating within a compact footprint and allows the device to operate at a maximum tuning power of just 60 mW. Additionally, the scalability of our MPCWs enables easy extension of the operational range to other wavelength bands. For spectrum reconstruction, we employ a solution decomposition-based alternating optimization algorithm (Fig. 1d), enabling high-fidelity and fast recovery of various types of spectra. Experimentally, the spectrometer achieves a 100 nm operating bandwidth with 0.5 nm resolution within 4.4 s, demonstrating its potential for high-performance, miniaturized spectral analysis in the MIR region.

## Results

### Reconstruction principle and algorithm

In our MPCW spectrometer, as depicted in Fig. 1a, the dual tuning of lattice constant $a$ and thermal power $P$ contributes to two key outcomes: distinct band edge and resonance positions (driven by $a$) and fine control over the band edge and resonance (enabled by $P$), respectively. In spectrum sampling, a specific MPCW unit and a specific thermal power correspond to a specific sampling step, and each of these steps can be expressed as $({a}_{i},{P}_{j})$, and the corresponding response is denoted as ${{{{\rm{T}}}}}_{({a}_{i},{P}_{j})}$. Figure 1b illustrates the single peak sampling process via both sharp band edges and non-uniform resonances at the corresponding steps. The sampling process of our MPCW units is governed by the following integral equation:

1 $${d}_{({a}_{i},{P}_{j})}=\int\limits^{{\lambda }_{{{{\rm{F}}}}}}_{{\lambda }_{{{{\rm{I}}}}}}{{{{\rm{T}}}}}_{({a}_{i},{P}_{j})}(\lambda )s(\lambda ){{{\rm{d}}}}\lambda$$

where ${\lambda }_{{{{\rm{I}}}}}$ and ${\lambda }_{{{{\rm{F}}}}}$ indicate the initial and final wavelengths of the spectral range to be reconstructed. ${{{{\rm{T}}}}}_{({a}_{i},{P}_{j})}\left(\lambda \right)$ defines the power transmission function of the sampling step $({a}_{i},{P}_{j})$, and $s(\lambda )$ denotes the input spectrum. The output power is represented by ${d}_{({a}_{i},{P}_{j})}$. For spectral integration problems, the continuous integral equation must be discretized for numerical solution. Specifically, by aggregating the responses of all channels, the transmission matrix **T** (or calibration matrix, as depicted in Fig. 1c) is constructed, and this matrix is then used to approximate Eq. (1) in matrix form:

2 $${{{\bf{d}}}}={{{\bf{T}}}}{{{\bf{s}}}}$$

Here, ${{{\bf{d}}}}\in {{\mathbb{R}}}^{m\times 1}$ is the detection vector, ${{{\bf{T}}}}\in {{\mathbb{R}}}^{m\times n}$ is the spectral response matrix (obtained via pre-calibration), and ${{{\bf{s}}}}\in {{\mathbb{R}}}^{n\times 1}$ is the discretized input spectrum. The number of sampling channels is $m$, corresponding to ${i}_{\max }\times {j}_{\max }$, and $n$ is the number of wavelength points.

However, in practice, measurement errors from noise and system imperfections can lead to instability and jagged reconstructions and restrict the direct solve the Eq. (2)^35^. In our work, the solution decomposition least-squares method is used to reconstruct the spectrum^28,36,37^:

3 $$\begin{array}{c}{\hat{{\bf{s}}}}={\hat{{\bf{s}}}}_{1}+{\hat{{\bf{s}}}}_{2},\left({\hat{{\bf{s}}}}_1,{\hat{{\bf{s}}}}_2\right)={\arg }{\min}_{{{\bf{s}}}_1,{{\bf{s}}}_2} {\| {{\bf{T}}}\left({{\bf{s}}}_{1}+{{\bf{s}}}_{2}\right)-{{\bf{d}}}\|}^{2}+{\alpha }^{2}{\| {{\bf{s}}}_{1}\|}_{1}+{\beta}^{2}\|{{{\bf{D}}{\bf{s}}}}_{2}\|_{2}^{2}\end{array}$$

This approach decomposes the spectrum ${{{\bf{s}}}}$ into a sparse component ${{\bf{s}}}_{1}$ and a smooth component ${{{{\bf{s}}}}}_{2}$ and applies ${L}_{1}$ regularization to ${{{{\bf{s}}}}}_{1}$ and ${L}_{2}$ regularization to ${{{{\bf{Ds}}}}}_{2}$, where ${{{\bf{D}}}}$ denotes the second-order derivative matrix. $\alpha$ and $\beta$ are independent regularization parameters controlling the strengths of the ${L}_{1}$ and ${L}_{2}$ regularizations, respectively. This formulation enhances both sparsity and smoothness in spectral reconstruction. In the actual calculation process, most studies employ a powerful tool, convex optimization, to directly minimize Eq. (3), with regularization parameters selected via K-fold cross-validation (CV). However, this approach incurs substantial computational costs, rendering it impractical for large-scale or real-time spectrum reconstruction. Additionally, K-fold CV requires repeated execution of the same optimization process, which undoubtedly introduces additional computational overhead. Compared to the convex optimization method with K-fold CV, the subspace iterative method is more suitable for the computational reconstruction spectrometer due to its computing speed^38^. It iteratively refines the solution by projecting the problem onto a low-dimensional subspace, updating the subspace in each iteration to focus on the most relevant components of the spectrum, thereby reducing the computational burden while maintaining reconstruction accuracy. However, most iterative methods typically only support a single regularization constraint. Here, we employ an alternating optimization framework built upon the conventional subspace iterative method to address this limitation, eliminating the need for algorithmic modifications. This scheme decomposes the optimization problem of Eq. (3) into two subproblems, one for ${L}_{1}$ regularization and another for ${L}_{2}$ regularization, and solves the sparse component ${\hat{{{{\bf{s}}}}}}_{1}$ and the smooth component ${\hat{{{{\bf{s}}}}}}_{2}$ independently with these subproblems (Fig. 1d and “Methods” section). Additionally, by minimizing the weighted generalized cross-validation (wGCV) function in each iteration, the optimal values of $\alpha$ and $\beta$ can be automatically determined without requiring any prior knowledge (see Supplementary Note 1 for details). This automatic parameter selection helps accelerate the computation and avoids repeatedly solving Eq. (3) when selecting the regularization parameters.

### Device design and fabrication

**Fig. 2:**
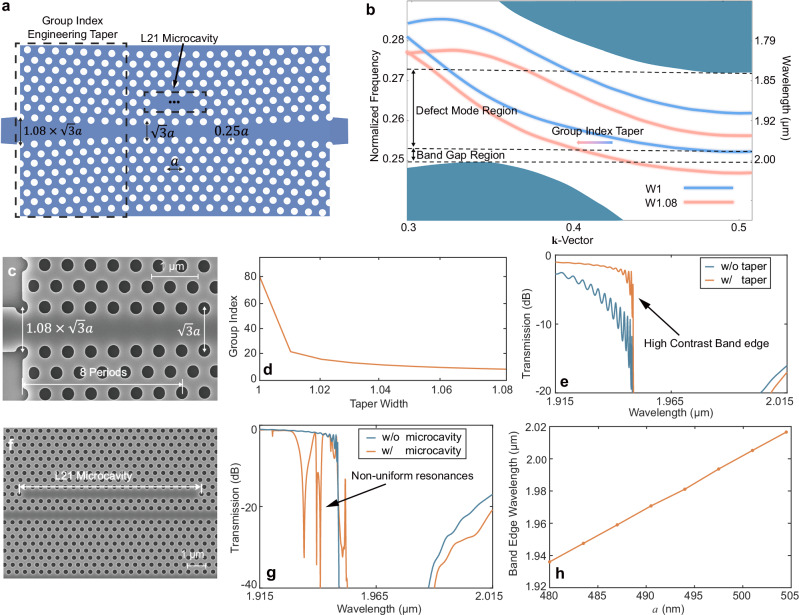
Design of the passive section of our MPCW. **a** Schematic of the proposed MPCW structure, consisting of an L21 PC microcavity side-coupled to a W1 PCW with a group index engineered taper. **b** Blue curves denote the W1 defect mode, while pink curves correspond to the W1.08 defect mode. The group index taper facilitates mode conversion from the strip waveguide to PCW. **c** SEM image of the fabricated group index taper. **d** Simulated group index variation along the taper as a function of taper width. **e** Simulated transmission spectra of the PCW with and without the group index taper, demonstrating its effect on spectral shaping. **f** SEM image of the fabricated MPCW, consisting of a W1 PCW and a side-coupled L21 PC microcavity. **g** Simulated transmission spectra of the PCW with and without the side-coupled microcavity, showing the enhanced filtering behavior enabled by non-uniform resonances. **h** Simulated relationship between the photonic band edge wavelength and the lattice constant $a$ of the MPCW.

Following the reconstruction principle, we design and fabricate the sampling units to achieve the transmission matrix ${{{\bf{T}}}}$. Our MPCW spectrometer was fabricated on a 340 nm SOI wafer with a 2 μm buried oxide layer and a 1 μm SiO_2_ top cladding. Fabrication was performed at the ShanghaiTech Material Device Lab (SMDL) using a process similar to our previous work (see “Methods” section)^39,40^. As Fig. 2a shows, each MPCW unit consists of a fully etched PCW with a group-index engineered taper and a side-coupled L21-type microcavity. According to the photonic band diagram (Fig. 2b), the PC adopts a hexagonal lattice with a period *a* ≈ 500 nm and hole radius *r* = 0.25*a*, placing the band edge near 2 μm. The W1-type PCW is formed by removing a single row of holes, with a width of $\sqrt{3}a$, supporting guided modes. Due to the group index ${n}_{g}$ mismatch between the conventional strip waveguide and PCW, Fresnel reflection occurs, degrading performance^41^. To address this, a group index taper is implemented, increasing PCW width from $\sqrt{3}a$ (W1) to $1.08\times \sqrt{3}a\,$(W1.08) over eight lattice periods (step size: $0.01a$), as shown in the scanning electron microscope (SEM) image in Fig. 2c. Figure 2d illustrates that ${n}_{g}$ reduces from >80 (W1) to ~8 (W1.08), matching that of a conventional waveguide ( ~ 4.4). Further extending the taper length yields diminishing returns. Three-dimensional finite-difference time-domain (3D-FDTD) simulations demonstrate that tapering reduces Fresnel reflection (Fig. 2e) and improves the contrast of the band edge, yielding a sharp 3 dB roll-off of 0.05 nm and a high roll-off rate of ~62.5 dB nm^−1^, which is crucial for spectrometer resolution.

Although our device primarily performs sampling through band edges, due to fabrication errors, it is not fully guaranteed that the band edge shifts induced by varying the lattice constant $a$ are entirely accurate and equally spaced. For PCWs without the microcavity, the spectrum at wavelengths shorter than the band edge is nearly flat, making it challenging to determine the wavelength at which the detected energy originates. We further aim to utilize light energy at wavelengths shorter than the band edge of the PCW to provide additional information. When a microcavity is side-coupled to the PCW, energy at resonant wavelengths is extinguished within the cavity^42,43^. This extinction introduces more variations at wavelengths shorter than the band edge. If the cavity is too short, the number of resonant dips is insufficient. Conversely, while excessively long microcavities generate more resonances with higher Q-factors in the response spectrum, they also reduce the roll-off of the band edge (Supplementary Note 2, Fig. S1). Additionally, since resonances manifest as a dip (downward valleys) in the response spectrum, these dips introduce additional spectral variations. Consequently, a higher Q-factor is not always beneficial in our context. Compared to the Q-factor, the FSR of resonances is more interesting. The FSR of this microcavity is given by:

4 $${{{\rm{FSR}}}}(\lambda )=\frac{{\lambda }^{2}}{{n}_{g}(\lambda )L}$$

where $\lambda$ is the resonance wavelength and $L$ is the cavity length. From Fig. 2b, ${n}_{g}$ is relevant to the wavelength and corresponds to a non-uniform FSR. This non-uniform FSR could break the reconstruction limitation of FSR. Thus, to balance the number of resonances and the roll-off of the band edge, an L21 microcavity is selected for side-coupling to the PCW (Fig. 2f). The MPCW’s simulated transmission (Fig. 2g) shows enhanced resonance features compared to the conventional PCW, improving information density within a broad wavelength range.

One key advantage of PC structures is their inherent scalability, as the band edge position can be precisely tuned by adjusting the lattice constant $a$^24^. Fig. 2h illustrates that varying $a$ from 480 to 504.5 nm (step size: 3.5 nm) spans a spectral range of 1915 − 2015 nm. In Supplementary Note 2, we also simulated the corresponding relation between different working wavebands (from 1.55 to 3.5 μm) and lattice constant $a$ to demonstrate the scalability of our MPCW (Fig. S2). To implement multi-channel sampling, a cascaded $1\times 2$ and two $1\times 4$ multimode interferometers (MMIs) are used to split input power across 8 MPCW units, each with a distinct $a$.

**Fig. 3:**
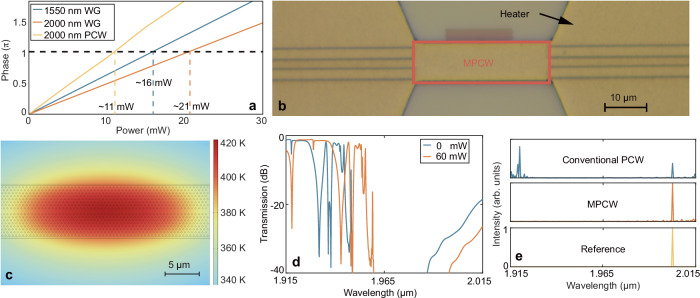
Design of the active section of our MPCW. **a** Comparison of simulated TO tuning efficiency for conventional strip waveguides (1550 nm and 2000 nm) and the PCW operating at the wavelength of 2000 nm. **b** Optical image of the MPCW covered by a Ti heater. **c** Top view of the simulated steady-state thermal distribution in the PC device at 60 mW heating power. **d** Simulated transmission spectra of the MPCW under heating powers of 0 mW and 60 mW. **e** Comparison of reconstruction performance under 7% random detection noise. The yellow line represents the original input spectrum, the blue line corresponds to the spectrum reconstructed using a conventional PCW, and the orange line denotes the spectrum reconstructed using the MPCW.

For the active section, TO modulation is integrated into our spectrometer design to enable fine-tuned control of the PC band edge. At longer wavelengths, achieving sufficient phase modulation typically requires larger waveguide cross-sectional dimensions and longer π-shift lengths. However, due to the slow-light effect near the PC band edge, thermal tuning efficiency can be significantly enhanced compared to conventional strip waveguides. To quantify this improvement, we simulated the π-phase shift power (${P}_{\pi }$) for standard silicon strip waveguides at both 1550 nm (500 × 220 nm^2^) and 2000 nm (650 × 340 nm^2^), alongside the PCW used in our spectrometer. Details of the simulation procedure are provided in Supplementary Note 3. As shown in Fig. 3a, the ${P}_{\pi }$ of the strip waveguide designed for 1550 nm is ~16 mW, while the waveguide designed for 2000 nm requires ~21 mW. In contrast, the PCW in our spectrometer achieves a significantly lower ${P}_{\pi }$ of ~11 mW near the band edge, demonstrating the enhanced thermal efficiency enabled by the slow-light effect. An optical image of the MPCW with the integrated Ti heater is presented in Fig. 3b. We further simulated the thermal distribution of the PCW under a 60 mW heating condition, as shown in Fig. 3c, S3 and S4, which reveals a temperature gradient of ~2.05 K mW^−1^ (details in Supplementary Note 3). Incorporating this thermal perturbation into an FDTD model, we estimate a band edge shift of approximately ~0.182 nm mW^−1^ (Fig. 3d). In the MPCW spectrometer, each MPCW unit is independently addressable via microheaters connected by aluminum (Al) wires, allowing precise and localized control of the spectral response for each output channel. Figs. S6 and S7 depict the simulated multi-channel TO modulated spectral responses matrices of a conventional PCW and our optimized MPCW, highlighting substantial improvements in spectral sampling and reconstruction fidelity. To assess robustness under realistic operating conditions, we introduce 7% random noise into the detection vector before spectral reconstruction (Fig. 3e, details in Supplementary Note 4). Even under this perturbation, our optimized MPCW structure demonstrates significantly improved reconstruction accuracy compared to the conventional PCW design.

### Device response and spectrum reconstruction

**Fig. 4:**
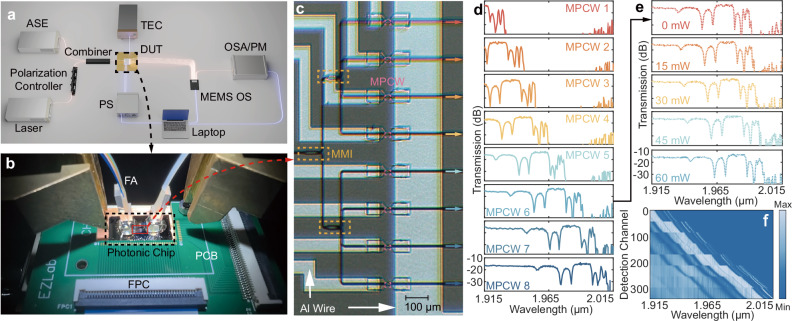
Experimental setup, devices characterization and calibration. **a** Schematic of the experimental setup. Red lines indicate optical paths (fiber), and blue lines indicate electrical control and data acquisition. ASE: amplified spontaneous emission source; PS: power supply; DUT: device under test; TEC: thermoelectric cooler; MEMS OS: micro-electromechanical systems optical switch; OSA: optical spectrum analyzer; PM: power meter. **b** Image of the packaged chip mounted on a printed circuit board (PCB). FA: fiber array; FPC: flexible printed circuit. **c** Optical microscope image of the MPCW spectrometer. The pink dashed box denotes the MPCW unit, and the orange dashed box represents the MMI. White wires are Al electrical connections, while black lines correspond to photonic waveguides. The passive measurement results of each channel are depicted in (**d**) with corresponding colors. **d** Measured transmission spectra of each MPCW channel, illustrating the shift of band edge and resonance wavelengths with increasing lattice constant. **e** Thermally tuned transmission spectra of the 6^th^ MPCW channel under heating powers of 0, 15, 30, 45, and 60 mW. In **d**, **e**, the increased spectral noise in the longer-wavelength range is attributed to reduced source power at these wavelengths. **f** Measured transmission matrix ${{{\bf{T}}}}$ of the MPCW spectrometer, constructed through calibration.

The experiment setup is shown in Fig. 4a. An amplified spontaneous emission (ASE) source and laser sources (AdValue Photonics and OELTS-300) were used as input light sources. The packaged chip (Fig. 4b) was mounted on a thermoelectric cooler (TEC) to maintain a stable ambient background temperature of 23 °C. Packaging procedures are detailed in the “Methods” section. Electrical power was supplied via a Keysight E36312A source, connected through a flexible printed circuit (FPC) to a printed circuit board (PCB). As depicted in Fig. 4c, the Ti heaters were connected with Al wires (white), with all components sharing a common ground. To ensure repeatability and measurement stability, we adopted a nonlinear control optimization strategy for regulating heating power rather than directly setting voltage. The light was coupled into and out of the chip using focusing subwavelength grating couplers^44^. Since our MPCW spectrometer has eight output channels, we employed an external micro-electromechanical system (MEMS) optical switch to sequentially connect each channel to a single detector, ensuring consistent and stable output power measurements.

We first characterized the passive transmission spectra of each MPCW channel (without thermal tuning) using a commercial optical spectrum analyzer (OSA, Yokogawa AQ6376). Figure 4d presents the normalized transmission spectra (relative to the grating coupler) of each MPCW channel. The average band edge shift between adjacent MPCW units is around 11.3 nm, corresponding to a shift ratio of about 3.2 per unit change in the PC lattice constant. The fabrication tolerance, additional loss data for individual MPCW units, and polarization stability are provided in Supplementary Notes 5–7. These passive spectral responses effectively cover the entire target spectral range from 1915 to 2015 nm. To achieve fine spectral sampling, we applied thermal tuning via integrated microheaters. Figure 4e shows the measured transmission spectra of the 6^th^ MPCW channel at heating power levels of 0, 15, 30, 45, and 60 mW. Although the heating of MPCW channels is independent, the analysis of temperature perturbation and thermal crosstalk is presented in Supplementary Note 8 and 9. The observed band edge shift rate was approximately 0.158 nm mW^−1^, closely matching our simulated ~0.182 nm mW^−1^ result. Figure 4f depicts the calibration matrix **T**, and the repeatability of the calibration matrix is displayed in Supplementary Note 10. To construct this matrix, we calibrated the MPCW spectrometer by measuring the output spectra for each channel across heating powers ranging from 0 to 60 mW, in 1.5 mW steps (corresponding to 41 times output spectrum measurement at different thermal powers), using the OSA at 0.5 nm resolution. The measured spectra were normalized to the light source, thus incorporating the grating coupler responses into the effective sampling function. By aggregating these thermal tuning responses of 8 MPCW channels, we get a 328 measurement channels calibration matrix $\left(m=328\right)$.

**Fig. 5:**
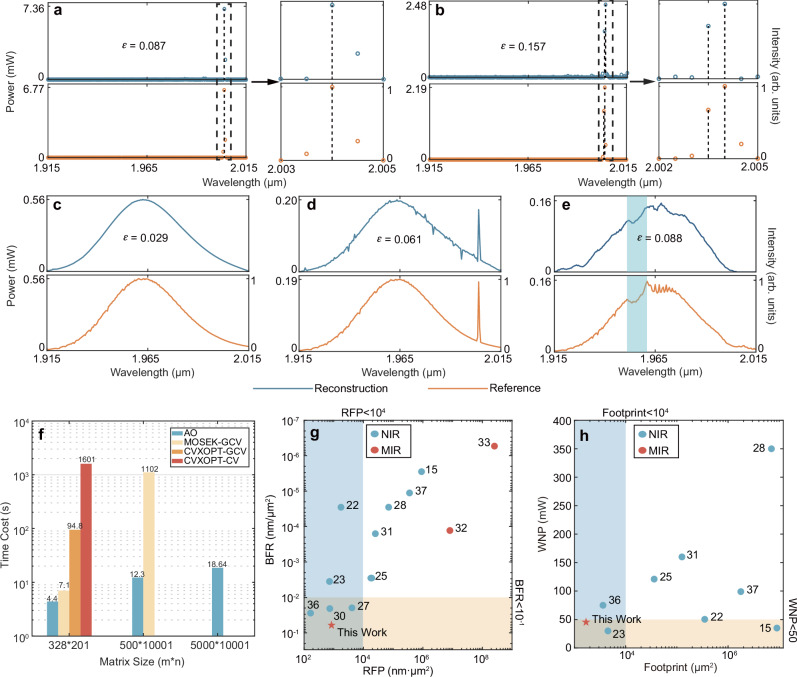
Reconstruction results and performance comparison. **a**–**e** The blue line is the algorithm reconstruction result, and the orange line is the reference measured by the commercial spectrometer. $\varepsilon$ denotes relative reconstruction error. **a** Single peak input spectrum and its reconstructed result. The peak is at 2004 nm. The right side shows an enlargement of the left side, which exhibits a full width at half maximum (FWHM) of 0.5 nm. **b** Dual peaks reconstructed result. An additional peak is set at 2003.5 nm adjacent to the 2004 nm peak. The right side displays a zoomed-in view of the left side. Compared to the (**a**), the reconstruction result displays the additional peak line. **c** Broadband ASE input spectrum and its reconstructed result. **d** The input hybrid spectrum, which combines the ASE and a laser at 2004 nm. **e** Spectrum of ASE after absorption by carbon dioxide and its reconstruction result. The blue region represents the reconstructed absorption envelope. **f** Reconstruction efficiency of different methods for different matrix dimensions. For larger-scale problems, the reconstruction time of our alternating optimization does not increase significantly. Direct solution via convex optimization was implemented using CVXPY (solver: CVXOPT and MOSEK), while the alternating optimization method was carried out with the IRtools. Calculations were all performed on a laptop with a 10-core Apple M1 Pro CPU and 16 GB of RAM. AO: alternating optimization; MOSEK-GCV: convex optimization with MOSEK backend with GCV; CVXOPT-GCV: convex optimization with CVXOPT backend with GCV; CVXOPT-CV: convex optimization with CVXOPT backend with 10-fold-CV. The spectrometer performance is compared with the focus on **g** resolution-footprint product (RFP) and bandwidth-footprint ratio (BFR), **h** wavelength-normalized-power (WNP) and footprint.

After calibration, we validated the spectrometer’s reconstruction performance across various input spectra. To solve Eq. (3) and compute $\hat{{{{\bf{s}}}}}$, we employ the IRtools, a MATLAB package for solving inverse problems^45^. Figure 5a–e compare the reconstructed spectra $\hat{{{{\bf{s}}}}}$ (blue curves) with reference spectra ${{{\bf{s}}}}$ measured by the OSA (orange curves). Here we use relative error $\varepsilon=\frac{||\hat{{{{\bf{s}}}}}-{{{\bf{s}}}}|{|}_{2}}{||{{{\bf{s}}}}|{|}_{2}}$ to evaluate the accuracy of reconstruction. Figure 5a shows the reconstruction of a single peak laser (2004 nm) spectrum, highlighting the system’s 0.5 nm resolution. In addition, we also retrieved the dual-peak spectrum (shown in Fig. 5b), with an additional peak at 2003.5 nm adjacent to 2004 nm. Compared to Fig. 5a, b, the reconstruction results demonstrate that our spectrometer is sensitive to the additional peak and can achieve successful reconstruction at a 0.5 nm resolution. We also reconstructed the laser spectrum in the 1970–2015 nm range and compared it with the spectrum measured by the OSA with 0.5 nm resolution (Supplementary Note 11). In addition, the measurement results for the detection limit are presented in Supplementary Note 12. Figure 5c demonstrates the reconstruction of a broadband ASE spectrum, confirming a 100 nm operational bandwidth. Additionally, the reconstruction result of a hybrid spectrum generated from ASE and a laser source is shown in Fig. 5d, which validates the performance of the alternating optimization method for the spectrum with both smooth and sparse features. In addition, we constructed a prototype 1‑m‑long carbon dioxide gas cell. Following the alignment of two collimated fibers, the 2 μm ASE light source was coupled into the gas cell to acquire the absorption spectrum. Due to the lack of precise flow control in the carbon dioxide delivery system, the gas concentration fluctuated during the sampling process during the sampling process, leading to a degradation in reconstruction fidelity. The carbon dioxide absorption envelope remains clearly discernible in the reconstructed spectrum in Fig. 5e. Reconstruction noise of the laser source (AdValue Photonics) itself is detailed in Supplementary Note 13. To verify the consistency of the MPCW spectrometer, five independent sampling trials were conducted for each scenario, and the mean and standard deviation of the reconstruction errors were calculated under different numbers of sampling channels. The results are presented in Supplementary Note 14. These results also confirm the effectiveness of our reconstruction algorithm, even under moderate noise levels.

Additionally, we compared the average reconstruction speed of different methods (Fig. 5f) using the test cases presented in Fig. 5a–e. Furthermore, we also examined the computation time of these methods for larger-scale calibration matrices. The time shown in Fig. 5f includes the parameter search duration. These methods include the direct solution [Eq. (3)] via convex optimization (using CVXOPT and MOSEK solvers), which searches for regularization parameters through K-fold CV and GCV methods (*K* = 10, see Supplementary Note 15 for details on CV and GCV), and the alternating optimization method with automatic parameter selection. For convex optimization, it is worth noting that parameter searching in CV is time-consuming. Reducing search time may lead to failing to identify the optimal regularization parameters, resulting in larger reconstruction errors than with the alternating optimization method. The GCV methods in convex optimization perform well when the matrix size is small. However, as the size of the calibration matrix increases, the optimization overhead and the computational cost of GCV function calculation increase significantly, resulting in a higher overall computation time. For alternating optimization, since the problem is solved within the projected subspace, the computation time does not increase significantly. Moreover, since the GCV value is computed within a subspace, the computational burden is significantly reduced.

## Discussion

By integrating a group-index-engineered taper and a microcavity into the PCW, we effectively establish a short-wavelength pass-band edge filter in the MIR. The resulting high optical power contrast and non-uniform resonances enhance sampling performance, contributing to the high resolution observed in spectral reconstruction. While most previous on-chip spectrometers target the 1.55 µm near-infrared band, our device uniquely achieves high resolution and broad bandwidth within a small footprint in MIR. Notably, the inherent wavelength scaling for 2 µm MIR is approximately 1.29 times that of 1.55 µm, and this necessitates proportionally larger waveguide dimensions, which in turn leads to increased thermal power requirements for effective refractive index modulation. However, leveraging the slow-light effect near the PC band edge dramatically enhances thermal tuning efficiency, surpassing that of conventional strip waveguide devices. The combination of non-uniform FSR microcavities, PC band-edge filtering, and efficient thermal tuning enables the capability to resolve spectral features at a resolution of 0.5 nm across a 100 nm wavelength range centered at 2 μm.

Figure 5g, h compare our MPCW spectrometer with prior works, showing better performance in resolution-footprint product (RFP) and bandwidth-footprint ratio (BFR). The wavelength-normalized power (WNP), defined as ${P}_{{{{\rm{working}}}}}\times \frac{{P}_{\pi }\left(1550\right)}{{P}_{\pi }\left({{{\rm{center\; wavelength}}}}\right)}$, also demonstrates the enhancement enabled by the slow-light effect. The detailed performance comparison is summarized in Table S1 (Supplementary Note 16). We further evaluated computational efficiency by varying the dimensionality of the calibration matrix. The results in Fig. 5f highlight the potential of the alternating optimization method for larger-scale computational reconstruction spectrometer applications. The computational time for convex optimization increases rapidly with matrix dimension and becomes intractable during parameter searching, whereas the provided alternating iteration method shows no significant increase. Additionally, the system is readily scalable by adding more MPCW channels with varied lattice constants. Figure 2h and S3 confirm that the scalability of our MPCWs offers a straightforward approach to extend the operational range. Collectively, these results establish our MPCW platform as a promising candidate for high-resolution, broadband, and energy-efficient on-chip spectrometry in the MIR region, opening expanded possibilities for integrated MIR photonics.

## Methods

### Alternating optimization reconstruction algorithm

We provide an algorithm to solve the estimated spectral solution $\hat{{{{\bf{s}}}}}$. The algorithm follows an alternating iterative approach:

**Table:** 

Solving Eq. (3) using alternating optimization
1: Initialize ${{{{\bf{s}}}}}_{1}^{(0)}=0,\,k=1$ 2: While the stopping criteria are not satisfied, do 3: Solve ${{{{\bf{s}}}}}_{2}^{(k)}=\arg{\min}_{{\bf{s}}_{2}}||{{{\bf{T}}}}{{{{\bf{s}}}}}_{2}-({{{\bf{d}}}}-{{{\bf{T}}}}{{{{\bf{s}}}}}_{1}^{(k-1)})|{|}^{2}+{\beta }^{2}||{{{\bf{D}}}}{{{{\bf{s}}}}}_{2}|{|}_{2}^{2}$ 4: Solve ${{{{\bf{s}}}}}_{1}^{(k)}=\arg{\min}_{{{\bf{s}}}_{1}}||{{{\bf{T}}}}{{{{\bf{s}}}}}_{1}-({{{\bf{d}}}}-{{{\bf{T}}}}{{{{\bf{s}}}}}_{2}^{(k)})|{|}^{2}+{\alpha }^{2}||{{{{\bf{s}}}}}_{1}|{|}_{1}$ 5: Update: $k=k+1$ 6: End while 7: Return $\hat{{{{\rm{s}}}}}={{{{\rm{s}}}}}_{1}^{(k)}{{{\boldsymbol{+}}}}{{{{\rm{s}}}}}_{2}^{(k)}$

### Device fabrication method

The fabrication process begins by spin-coating ZEP-520A electron beam resist onto a commercial SOI wafer with a 340 nm device silicon layer. The device patterns are defined using high-resolution electron beam lithography (EBL). After development, the exposed silicon is etched using inductively coupled plasma reactive ion etching (ICP-RIE) to form the photonic structures, including waveguides and PC components. Following the silicon etching, a 1 μm-thick SiO_2_ cladding layer is deposited across the entire device via plasma-enhanced chemical vapor deposition (PECVD) to provide optical isolation and structural protection. A positive photoresist (AZ5214) is then spin-coated on top of the SiO_2_ layer, and the heater pattern is defined using a maskless aligner (MLA), which enables rapid and flexible patterning. Subsequently, the metal layer is deposited using sputtering. The heater electrodes are first formed by depositing a Ti layer and defining the pattern through a standard lift-off process. A second metal deposition and lift-off step is then performed to deposit Al wiring, which electrically connects the microheaters to external control circuitry. This two-step metal process ensures independent optimization of the heater and interconnect materials.

### Packaging method

The fabricated photonic chip is mounted onto a PCB, which is itself affixed to thin copper heat sinks using thermally conductive epoxy to enhance thermal stability. Electrical connections between the on-chip electrodes and the PCB are established through Al wire bonding. For optical interfacing, a small droplet of UV-curable adhesive is applied to the chip's grating coupler array region. A motorized alignment stage is used to precisely position a fiber array above the grating couplers. Once aligned, the adhesive is cured by exposing the assembly to UV light from four directions for 10 seconds. To further reinforce mechanical stability and maintain coupling efficiency, an additional layer of UV-curing adhesive is applied around the perimeter of the fiber array.

## Supplementary information

Supplementary Information

Transparent Peer Review file

## Data Availability

All the data supporting this study are available in the article and the Supplementary Note. Additional data related to this article are available from the corresponding authors upon request.

## Code availability

The codes supporting the results of this study are available from the corresponding authors upon request. Custom MATLAB code for spectral reconstruction via alternating optimization is available upon request.
