# Supplementary material for: An efficient mid-infrared computational spectrometer based on synergistic microcavity-coupled photonic crystal waveguides
Source: Nat Commun. 2026 Jun 3;17:7143. doi: 10.1038/s41467-026-73934-z (PMC13396208; doi:10.1038/s41467-026-73934-z)
Supplement: Supplementary file 1 — Supplementary Information [file 41467_2026_73934_MOESM1_ESM.pdf]

Supplementary Note

An efficient mid-infrared computational spectrometer based on synergistic microcavity-coupled photonic crystal waveguides

Lipeng Xia,[1,2,3,#] Yuhan Sun,[1,#] Jiahua Jiang,[4] Hong Zhang,[1] Weixiong Huang,[1] Yuheng Liu,[1] Chang Chang,[1,2,3] Yixiang Zhang,[1] Chaofeng Ye,[1] Yiming Ma,[5,*] Xiaochuan Xu,[6,*] Chengkuo Lee,[7,*] Yi Zou[1,*]

[1]School of Information Science and Technology, ShanghaiTech University, Shanghai, China

[2]Shanghai Institute of Microsystem and Information Technology, Chinese Academy of Sciences, Shanghai, China

[3]University of Chinese Academy of Sciences, Beijing, China

[4]Department of Mathematics, University of Birmingham, Birmingham, UK

[5]School of Microelectronics, Shanghai University, Shanghai, China

[6]State Key Laboratory on Tunable Laser Technology, Harbin Institute of Technology, Xili University Town, Harbin Institute of Technology campus, Shenzhen, Guangdong, China

[7]Department of Electrical and Computer Engineering, National University of Singapore, Singapore, Singapore

[#]These authors contributed equally.

[*]Corresponding authors: Yiming Ma (yimingma@shu.edu.cn), Xiaochuan Xu (xuxiaochuan@hit.edu.cn), Chengkuo Lee (elelc@nus.edu.sg), and Yi Zou (zouyi@shanghaitech.edu.cn)

1. Parameter Selection of Alternating Optimization

Our alternating optimization is based on the subspace iterative method. The regularization parameter can be selected during the iterative process by minimizing the weighted general cross-validation (wGCV). This function in the i-th iteration can be expressed as follows:

$$G_i(\gamma) = \frac{\|\mathbf{r}_i(\gamma)\|_2^2}{\left(trace\left(\mathbf{I}_i - \omega \mathbf{M}_i \mathbf{M}_i^{\#}(\gamma)\right)\right)^2},$$

where $\mathbf{r}_i(\gamma)$ denotes the projected residual of i-th iteration at the condition of the regularization parameter γ (can be α or β), ω is the weighting coefficient and $\mathbf{I}_i$ indicate the identity matrix with the dimension of $i \times i$. $\mathbf{M}_i$ is the lower bidiagonal matrix generated from the iterative process. $\mathbf{M}_i^{\#}(\gamma)$ is a "fictive" matrix that defines the regularized solution. For example, the $\mathbf{M}_i^{\#}(\gamma)$ of the L_2 regularization solution with the second-order derivative matrix can be expressed as:

$$\mathbf{M}_i^{\#}(\gamma) = \left(\mathbf{M}_i^{\dagger}\mathbf{M}_i + \gamma^2\, \mathbf{D}^{\dagger}\, \mathbf{D}\right)^{-1} \mathbf{M}_i^{\dagger},$$

† denotes the transpose of a real matrix. The second-order derivative matrix $\mathbf{D}$ is defined as:

$$\mathbf{D} = \begin{bmatrix} \ddots & \ddots & & & \\ \ddots & -2 & 1 & 0 & \\ & 1 & -2 & 1 & \\ & 0 & 1 & -2 & \ddots \\ & & & \ddots & \ddots \end{bmatrix}.$$

To get a good reconstruction result, the value of ω in our experiment is set to 0.01 for L_1. Thanks to the subspace iterative method, the value of the wGCV function can be calculated in each iteration, allowing for the optimization of regularization parameters instead of searching for the optimal ones after completing the whole solving process. The alternating optimization method enables the independent estimation of L_1 and L_2 regularization parameters within their respective subproblems. During the subproblem solution, the L_2 section is solved using the LSQR algorithm, while the L_1 section can be solved by either the flexible-LSQR (FLSQR) or the modified residual norm steepest descent method (only for non-negative) solver.

2. Microcavities and Scalability Analysis

Fig. **S1** shows the simulated transmission spectra of H1, L21, and L61 MPCWs. The number of resonant dips in H1 MPCW is insufficient. The L61 MPCW generates more resonances with higher Q-factors but also reduces the roll-off of the band edge.

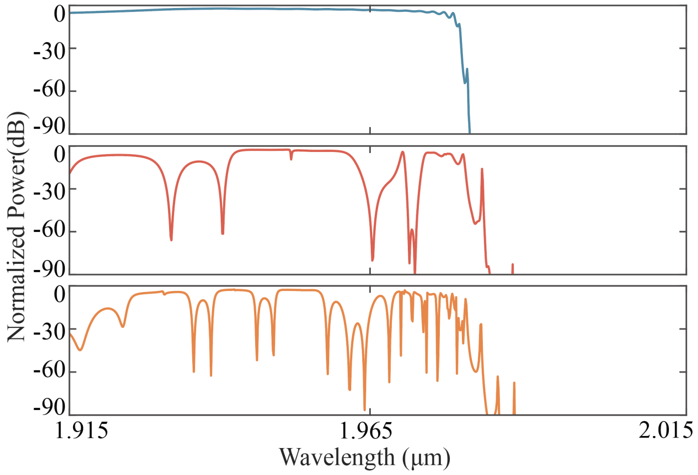

Fig. **S1**. Simulated H1, L21, and L61 MPCWs transmission spectra. Blue, red, and orange lines correspond to H1, L21, and L61, respectively.

Fig. **S2a** illustrates the relationship between band edge wavelengths (ranging from 1.5 μm to 3.5 μm) and lattice constant a, further demonstrating the scalability of our MPCW spectrometer. In addition, a PCW operating at the 3.5 μm waveband with a 1.02 μm lattice constant and a 50% duty cycle was fabricated on a 340 nm SOI wafer. The experiment was conducted using a Daylight Solutions Sidekick laser (with a wavelength range of 3.3–3.7 μm) and a Yokogawa AQ6377 spectrometer to measure the response spectrum of the PCW (Fig. **S2b**, normalized to a reference grating), achieving an insertion loss of 3.1 dB, which enables operation in the 3.5 μm waveband. The response spectrum in the figure can verify the scalability, which can be realized by shifting the bandgap and band edges to different wavelengths.

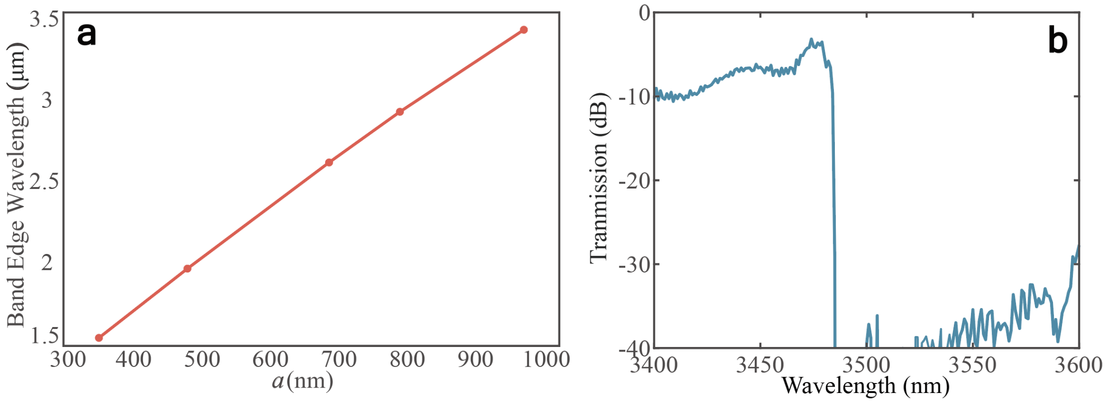

Fig. **S2 a** The relationship between band edge wavelength and a. **b** Experimental verification of wavelength scalability near 3.5 μm region.

3. Thermal Efficiency Analysis and π-Shift Power (P_π) Calculation

Figs. **S3** and **S4** depict the simulated thermal distributions of the Ti microheater of the PC device (top view) and the PC device (cross-sectional view) at a heating power of 60 mW.

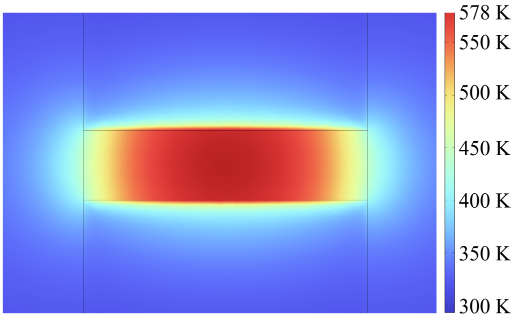

Fig. **S3**. Simulated thermal distribution in the top view of Ti microheaters under a heating power of 60 mW.

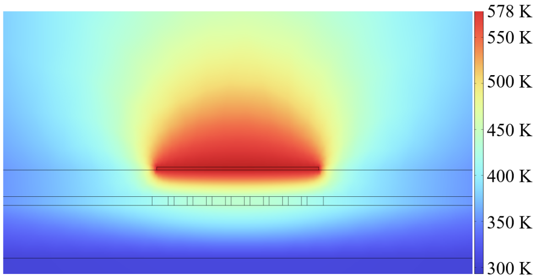

Fig. **S4**. Simulated cross-sectional thermal distribution of our PC devices at 60 mW heating power.

Strip Waveguide P_π Calculation procedure:

1. A 2D thermal simulation is conducted to determine the steady-state temperature distribution within the waveguide cross-section.
2. The extracted temperature profile is applied as a perturbation to the optical mode solver.
3. The change in effective refractive index (Δ_{neff}) is calculated.
4. P_π is computed based on the phase shift induced in a Mach-Zehnder interferometer (MZI) with a defined arm length difference.

PCW P_π Calculation procedure:

1. Due to a non-uniform profile along the propagating direction, a 3D thermal simulation is conducted to determine the volumetric temperature distribution across the PCW with a group index taper.
2. The thermal profile is incorporated into the FDTD simulation to capture the temperature-dependent shift in the PCW's S-parameters.

3. The modified PCW is embedded into the arms of a Mach-Zehnder interferometer, with a differential arm length of 100 μm.
4. Transmission characteristics are extracted to determine the required power for a π phase shift.
5. Fig. **S5** illustrates the simulated slow-light enhancement near the band edge, which significantly reduces P_π due to the increased thermo-optic interaction time.

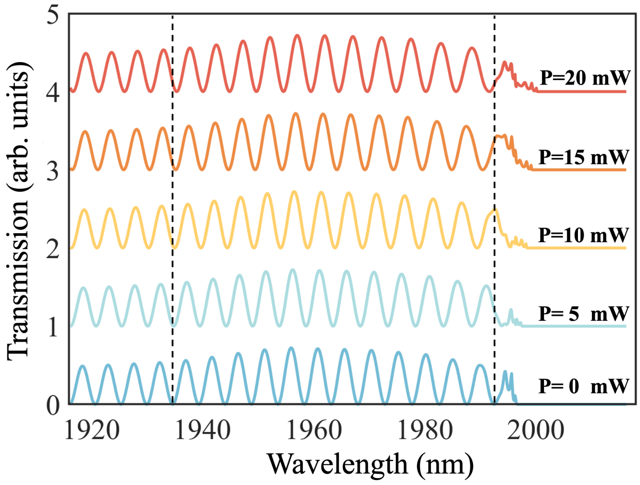

Fig. **S5**. Simulated transmission spectra of an MZI incorporating thermally modulated PCWs.

4. Reconstruction Performance Comparison

We simulate the sampling transmission matrices for both conventional PCWs (Fig. **S6**) and our optimized MPCW (Fig. **S7**) using the FDTD method. These transmission matrices are then used to model the spectrum sampling and reconstruction process.

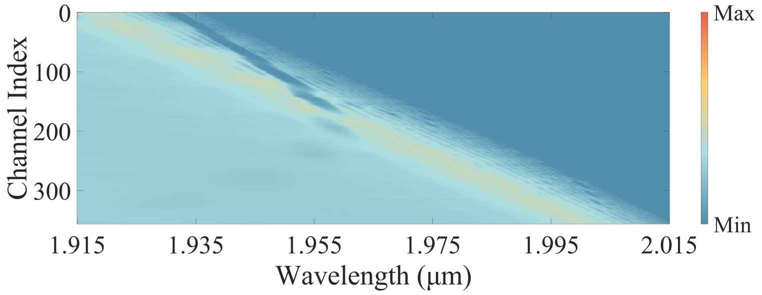

Fig. **S6**. Simulated sampling transmission matrix of a conventional PCW.

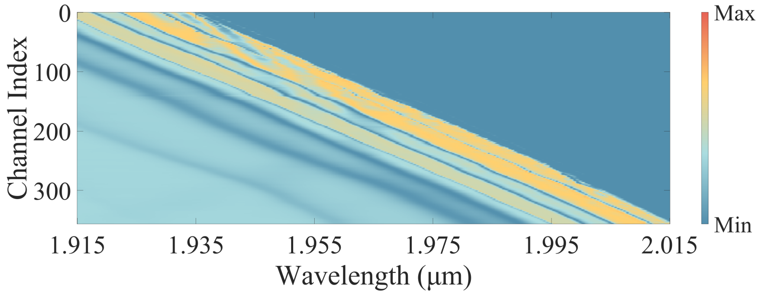

Fig. **S7**. Simulated sampling transmission matrix of the optimized MPCW.

To emulate realistic experimental conditions, a 7% random noise is introduced into the sampling process. Fig. **3e** shows the retrieved peak response under noisy conditions. The MPCW-based spectrometer exhibits superior spectral reconstruction accuracy and robustness compared to the conventional PCW, validating the effectiveness of our design optimization.

5. Fabrication Tolerance

During fabrication, the positioning error of electron-beam lithography is minimal, but the size of the fabricated holes is difficult to control precisely. Therefore, we simulated the influence of diameter deviations on the transmission characteristics of the filter. The simulation results are shown in Fig. **S8**. The results indicate that when the hole diameter error is within ±10 nm, the band-edge wavelength of the filter exhibits a red-shift or blue-shift of approximately 15 nm due to the variation in hole size.

In practical mass production, photolithography is typically used for the exposure process, and the hole size can be kept relatively stable across different batches. In addition, the impact of fabrication errors on the spectrometer can be reduced by adding two redundant MPCW units to compensate for spectral shifts due to response shifts caused by process tolerances.

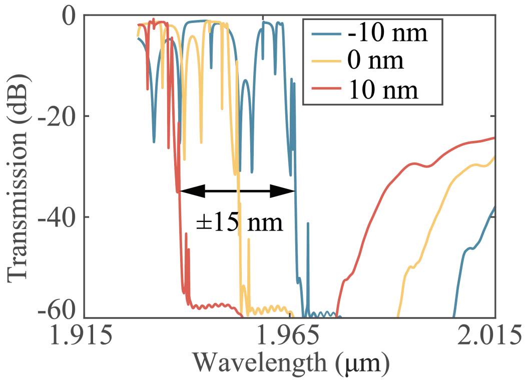

Fig. **S8**. Simulation results of response spectrum variation caused by fabrication errors in photonic crystal hole diameters.

6. Insertion Loss Characterization

Insertion loss in our system is primarily attributed to the MEMS optical switch, which is optimized for the C-band and therefore shows increased loss at the 2 µm wavelength range. Fig. **S9** presents the measured insertion loss, normalized to the amplified spontaneous emission (ASE) source spectrum.

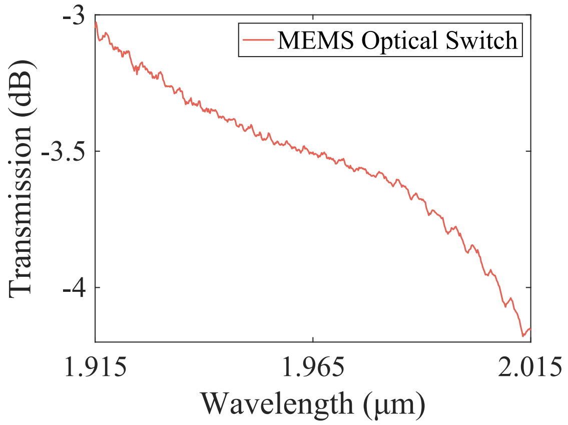

Fig. **S9**. Measured insertion loss of the MEMS optical switch around 2 µm, normalized to the ASE source.

In addition, we implement 1×2 and 1×4 MMIs for optical power splitting. Their measured transmission spectra, normalized to a reference grating coupler, are presented in Figs. **S10** and **S11**, respectively.

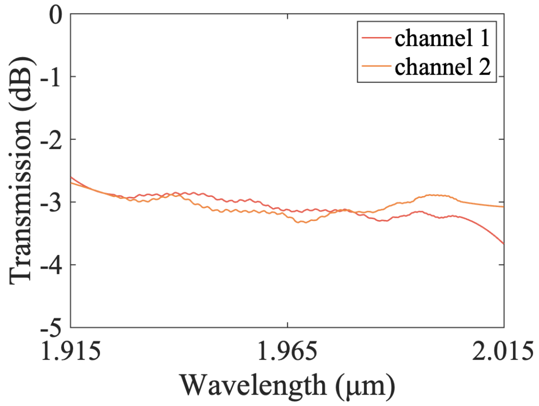

Fig. **S10**. Measured transmission spectra of the 1×2 MMI splitter, normalized to a reference grating coupler.

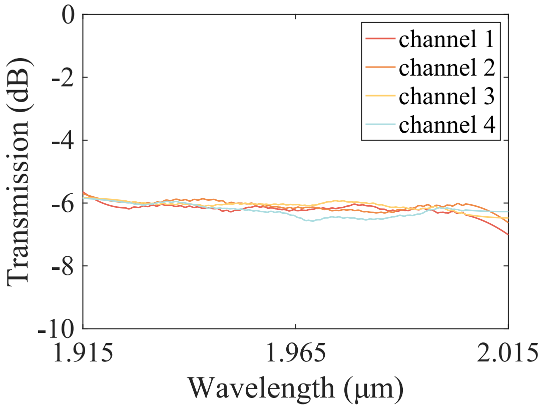

Fig. **S11**. Measured transmission spectra of the 1×4 MMI splitter, normalized to a reference grating coupler.

To isolate the contribution of the MPCW, we subtract the cumulative transmission spectra of all other passive components. The resulting normalized transmission spectrum of the MPCW is presented in Fig. **S12**.

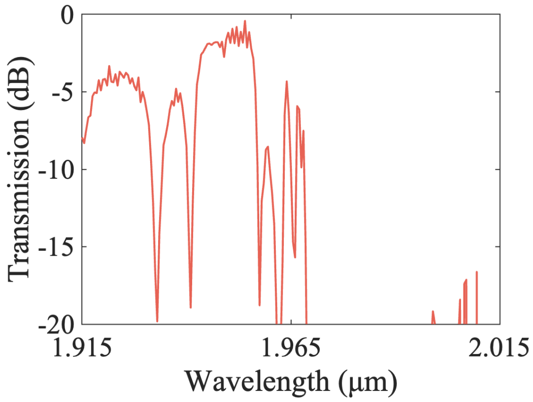

Fig. **S12**. Measured transmission spectrum of the MPCW device after normalization by subtracting the response of upstream optical elements.

7. Polarization Stability

During the experiment, we also used a polarization controller to adjust the polarization state of the input light, ensuring that the polarization of light passing through the grating coupler always matched the designed polarization direction of the grating. We separately recorded the laser output power, as well as the output power when TE-polarized and TM-polarized light was injected into the reference waveguide. Our grating coupler is a TE-polarized coupler, and the measurement results are shown in Fig. **S13**.

The laser output power is around 9.81 dBm (OELTS-300), with a power fluctuation of 3.11%. When the input light polarization matches the grating (TE polarization input), the optical power is stable at −5.82 dBm, with an output power fluctuation of 4.23%. When the input polarization is perpendicular to the selected polarization direction of the grating (TM polarization input), the output optical power is −37.5 dBm. Therefore, our grating coupler exhibits high polarization selectivity, with a polarization contrast ratio higher than 32 dB, and TM polarization does not influence our experiment. In addition, our MPCW operates only in the TE mode.

To ensure the power stability of the MPCW spectrometer, we fixed the transmission fiber using tape during the experiment to avoid excessive bending and torsion of the fiber, which would induce polarization changes and affect the response and output optical power of the MPCW.

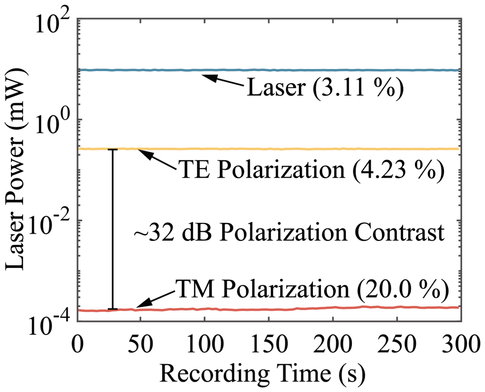

Fig. **S13**. Stability of the laser output power (blue) and the corresponding output optical power after inputting TE polarization (yellow) and TM polarization (yellow) into the reference grating. The polarization contrast of the grating is > 32 dB.

8. Temperature Perturbation

In our experiments, the spectrometer operates at room temperature, and we set the operating temperature to 23 °C. To evaluate the influence of temperature on the spectrometer, we measured the variation in the transmission characteristics of the filter over the temperature range from 20 °C to 26 °C (20.0, 21.0, 22.5, 22.9, 23.0, 23.1, 23.5, 24.0, 25.0, and 26.0 °C). The measurement results are shown in Fig. **S14a** and Fig. **S14b**. The transmission spectrum of the filter exhibits a red shift as the temperature increases. This phenomenon is attributed to the refractive index of silicon increasing with rising temperature, which shifts the band-edge position of the photonic crystal and consequently leads to the spectral drift of the filter.

To quantify the specific impact of temperature perturbation on the transmission spectrum of the filter, we used the relative variation $v(T)$ to characterize the change in its transmission behavior, defined explicitly as:

$$v(T) = \frac{\|R(T) - R(T_0)\|_2}{\|R(T_0)\|_2}$$

where $R(T)$ denotes the filter response spectrum at temperature T, and T_0 is the reference temperature (fixed at 23 °C here). Calculating the relative variation at different temperatures allows a clearer observation of the temperature effect on the filter transmission. The calculated results are displayed in Fig. **S14c**. It can be seen that the transmission characteristics of the filter change noticeably with increasing temperature, especially near the operating wavelength of the filter, where the variation is more significant. This indicates that ambient temperature fluctuations in non-laboratory environments can considerably affect the filter performance and highlights the necessity of integrating a TEC for precise thermal stabilization in practical applications.

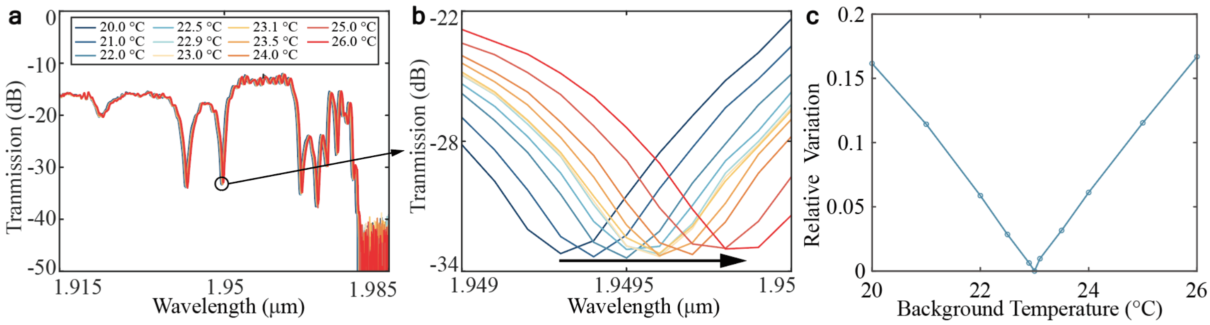

Fig. **S14**. **a** Response of the 6th MPCW channel as a function of temperature. **b** Magnified view of the transmission response **c** Relative spectral variation with reference to 23 °C.

9. Thermal Crosstalk

As the MPCW spectrometer contains multiple filter channels closely spaced, applying thermal power to a channel can affect the transmission characteristics of adjacent channels. Although our design is intended for single-channel operation, the measurements were conducted by sequentially heating and reading individual channels. The simultaneous excitation of multiple channels with multiple detectors would introduce additional thermal crosstalk.

To evaluate the effect of thermal crosstalk, we heated one channel and measured the transmission characteristics of its neighboring MPCW channels. The experimental results show that heating one channel alters the transmission spectrum of adjacent channels, with a relative spectral deviation of 11.1% compared with the unheated condition. Such thermal perturbation can be mitigated by introducing the air isolation region (air trench), as illustrated by our simulation results (Fig. **S15**). Without an isolation region, heat conducts directly through the silicon slab to neighboring channels, raising their temperature by approximately 1.72 K and perturbing their response spectra (10.1%), in good agreement with our experimental observations (Fig. **S15a**). With air isolation regions, heat conduction is effectively suppressed, reducing the temperature variation of adjacent channels to only 0.04 K (Fig. **S15b**), corresponding to a relative crosstalk of 0.4% (Fig. **S16**). This significantly alleviates thermal crosstalk.

Therefore, in multi-channel detection applications, deeply etched air isolation trenches can be adopted during the design and fabrication process to suppress thermal crosstalk and improve the stability and reliability of multi-channel spectral measurements.

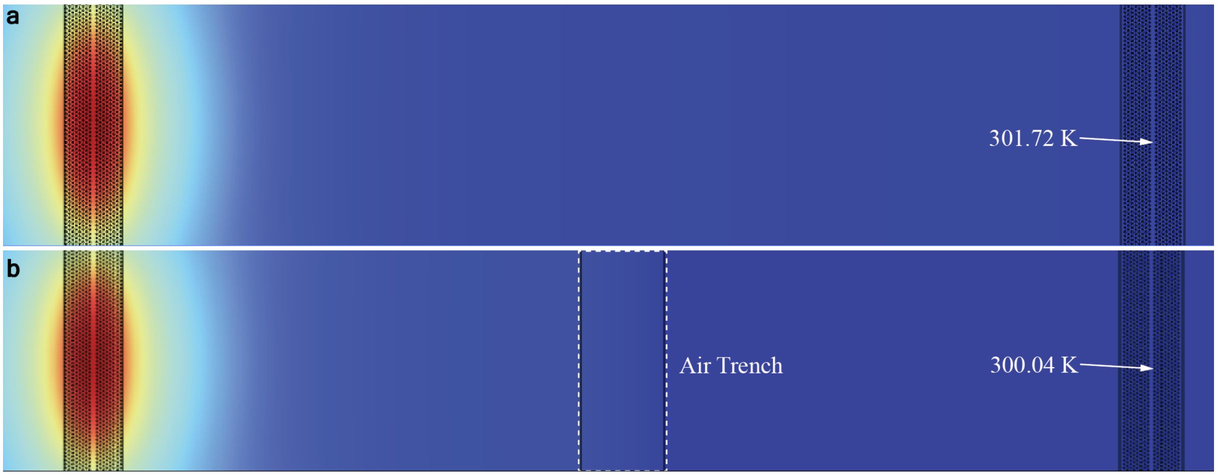

Fig. **S15**. Simulation results of heating perturbation **a** with and **b** without air trenches.

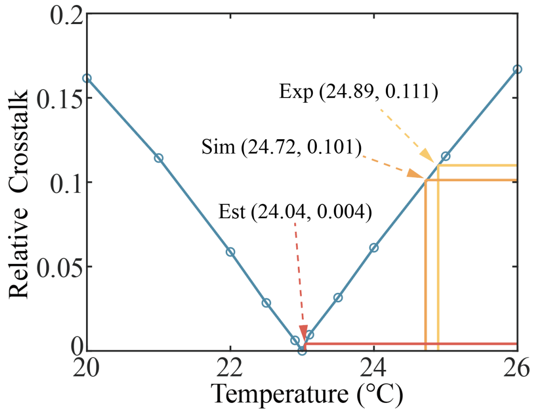

Fig. **S16**. Estimation of temperature perturbation after using air trenches based on simulation and experimental results.

10. Operation Repeatability

For the operational repeatability, we first measured the average relative fluctuation of the spectra of the MPCW element at 0 mW and 60 mW (with the TEC base temperature set to 23 °C). Similar to temperature-induced variations, the average spectrum fluctuation is defined as:

$$f(t) = \frac{\left\| R(t) - R_{average} \right\|_2}{\left\| R_{average} \right\|_2}$$

where $R(t)$ is the response spectrum acquired at time t, and $R_{average}$ is the average response spectrum taken at different times. As shown in Fig. **S17a**, the average relative fluctuation of the spectrum is approximately 0.9%, with the maximum fluctuation not exceeding 1.6%. These fluctuations are primarily attributed to the precision limit of the TEC controller.

Furthermore, we performed four complete measurements of the response matrix at 30-minute intervals. In each measurement cycle, the filters underwent a full heating and cooling cycle. We calculated the relative variation of the average transmission characteristics for each channel across different measurement rounds. As shown in Fig. **S17b**, the average relative fluctuation of the entire response matrix is roughly 0.7%. This demonstrates that the designed thermo-optically controlled photonic crystal filters maintain high operational repeatability across multiple thermal tuning cycles.

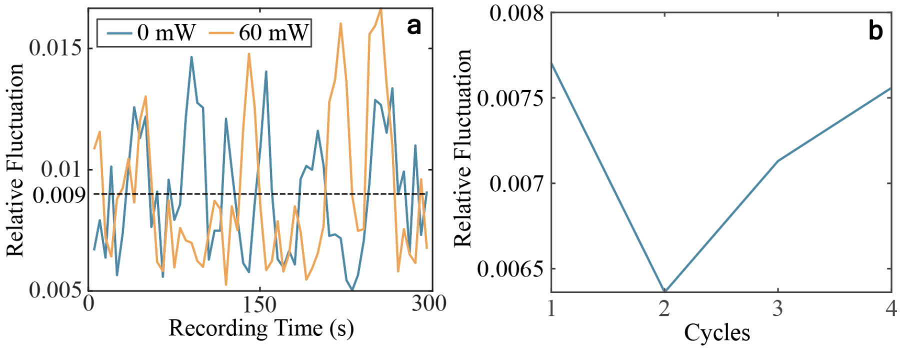

Fig. **S17**. **a** Fluctuations in the response spectra during long-term measurements at a specific heating power. **b** Fluctuations in the calibration matrix across multiple measurement cycles.

11. Spectral Reconstruction of Lasers at Different Wavelengths

Since our tunable laser (OELTS-300) operates in the range of 1970–2070 nm, which does not fully cover the operating range of our spectrometer. Thus, we reconstructed the laser spectrum within the range of 1970–2015 nm. The reconstruction results are shown in Fig. **S18**.

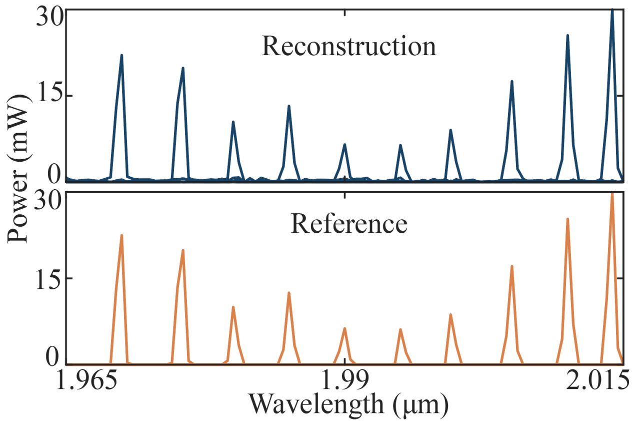

Fig. **S18**. Reconstructed spectrum of the tunable laser in the range of 1970–2015 nm. The blue curve represents the reconstruction result and the orange curve denotes the reference spectrum.

12. Detection Limit

A manual attenuator was utilized to regulate the output power of the laser. The output spectrum of the laser is shown in Fig. **S19**. The detection dark noise of our system is approximately −62 dBm. Notably, the reconstructed optical power exhibits significant deviations when the input power falls below 1 μW. To lower the detection threshold, the beam splitter in the MPCW spectrometer could be substituted with a thermo-optic switch, which can significantly improve the optical power budget.

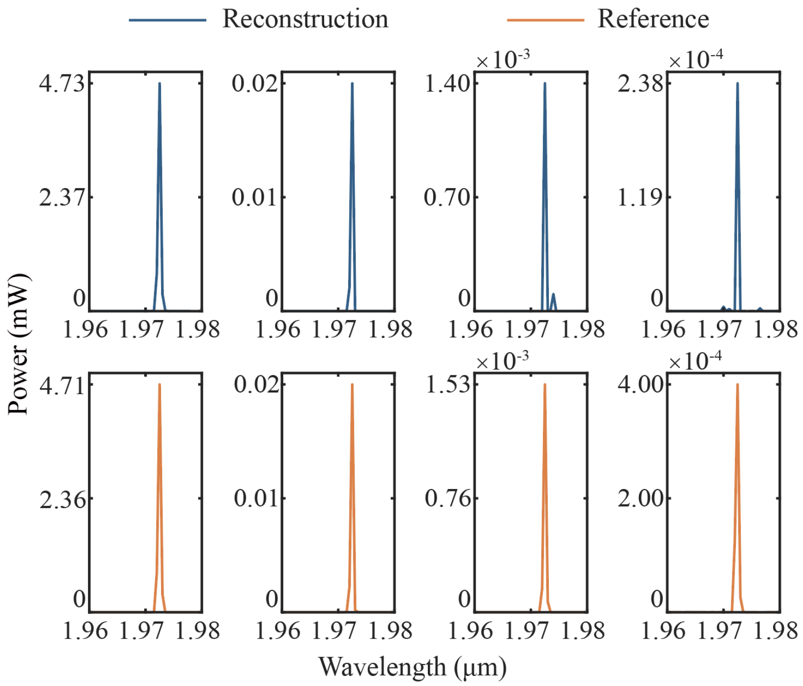

Fig. **S19**. Reconstruction results under different optical powers. The blue curve represents the reconstruction result, and the orange curve denotes the reference spectrum.

13. Laser Fluctuation Analysis

To quantify laser output stability, we monitor the laser (AdValue Photonics) power continuously for over 5 minutes with a sampling interval of 0.1 seconds, following a 30-minute warm-up to ensure thermal equilibrium. The temporal power fluctuations are illustrated in Fig. **S20**. The laser output power jitter is calculated as:

$$(P_{\mathrm{max}} - P_{\mathrm{min}})/P_{\mathrm{mean}} = 7.11\%.$$

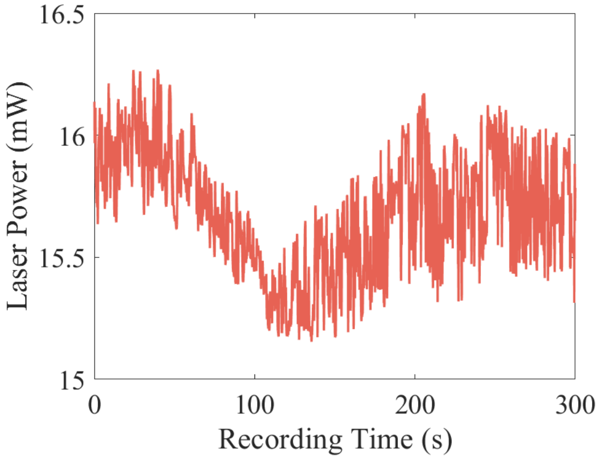

Fig. **S20**. Measured laser output power stability over 5 minutes following a 30-minute warm-up.

14. Reconstruction Consistency

To evaluate the reliability of the results, we performed an additional five sampling reconstructions for each case in the main text and calculated the mean ε_{mean} and standard deviation ε_{STD} of the reconstruction errors across these five independent reconstructions. In addition to our standard sampling (m = 328), we also adopted an under-sampling scheme for spectral reconstruction (m = 164). As illustrated in Fig. **S21**, ε_{mean} of narrow-band spectra (5a, 5b) are more sensitive to the number of channels, and finer sampling results in lower narrow-band reconstruction errors. In contrast, the reconstruction errors of broadband spectra (5c) are less sensitive to the number of sampling channels.

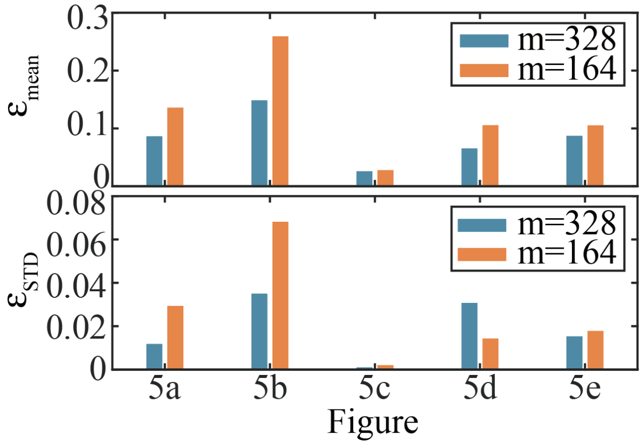

Fig. **S21**. The mean ε_{mean} and standard deviation ε_{STD} of reconstruction error based on Fig. 5 presented in the main text.

15. Selection of Regularization Parameters in Convex Optimization.

In the spectral reconstruction process of a computational spectrometer, cross-validation (CV) primarily partitions the detection channels of the calibration matrix (i.e., the rows of the matrix) into a reconstruction part (rp) and a validation part (vp). Spectral reconstruction is performed using the calibration results and detected power of the reconstruction part channels ($\mathbf{T}_{rp}$ and $\mathbf{d}_{rp}$) and evaluates the quality of regularization parameters by comparing the error between the predicted power ($\mathbf{T}_{vp}\hat{\mathbf{s}}_{rp}$), obtained by applying the reconstructed spectrum ($\hat{\mathbf{s}}_{rp}$) to the validation part channels, and the measured power $\mathbf{d}_{vp}$ of the validation part. This error can be expressed as:

$$\frac{1}{N_{vp}}\sum_{vp}\left(\mathbf{T}_{vp}\hat{\mathbf{s}}_{rp}-\mathbf{d}_{vp}\right)^2$$

N_{rp} and N_{vp} denote the number of channels in the reconstruction part and validation part, respectively.

The indices of all channels are shuffled, then partitioned into ten groups, each of which serves as the validation part in turn. For each validation group, the indices not included in the validation part form the reconstruction part. For each regularization parameter, the CV error of each group is calculated, and the optimal parameters are selected as those corresponding to the minimum CV error.

In practical implementation, we noticed that the computational time required for K-fold CV is excessively long. Additionally, we introduce a generalized cross-validation (GCV) function for selecting regularization parameters, which references the GCV function used in the subspace iteration method, though this GCV function is approximate for L_1 regularization parameter selection. The GCV function is defined as follows:

$$G(\alpha,\beta)=\frac{\|\mathbf{r}(\alpha,\beta)\|_2^2}{\left(trace(\mathbf{I}-\mathbf{T}(\mathbf{T}^{\dagger}\mathbf{T}+\alpha^2\,\mathbf{D}^{\dagger}\,\mathbf{D}+\beta^2\,\mathbf{F}^{\dagger}\,\mathbf{F})^{-1}\mathbf{T}^{\dagger})\right)^2}$$

where $\mathbf{r}(\alpha,\beta)$ denotes the residual at regularization parameters α and β. The matrix $\mathbf{F}$ can be expressed as:

$$\mathbf{F}=\mathrm{diag}\left(\left[2\sqrt{s_i^2+\epsilon}\right]^{-\frac{1}{2}}\right)_{i=1}^{n},$$

where s_i is the i-th element of the estimated vector $\hat{\mathbf{s}}_1$, and ϵ denotes a small value to avoid division by zero. The optimization procedure for the GCV method is as follows: initialize α and β, perform one round of solution using convex optimization tools, minimize the GCV

function with the obtained solution, update α and β, and repeat this process until the GCV values reach a plateau.

16. Comparison of On-Chip Spectrometers

The comparison of on-chip spectrometers is summarized in Table S1:

Table S1. Comparison of On-Chip Spectrometers

Structure	Footprint (μm^2)	WB (μm)	RES (nm)	BW (nm)	CH	Active?	WNP (mW)	RFP ($nm\cdot\mu m^2$)	BFR ($nm\cdot\mu m^{-2}$)
**Random disorder scattering**[1]	1250	NIR (1.51)	0.75	25	25	N	/	5625	0.02
**Stratified waveguide filters**[2]	9100	NIR (1.55)	0.45	180	32	N	/	4095	0.0198
**Cascaded nanobeam**[3]	4500	NIR (1.55)	0.16	16	3	Y	30	720	3.6×10^{-3}
**Grating-assisted F-P cavity**[4]	3.6×10^{4}	NIR (1.50)	0.43	73.2	5	Y	121	1.84×10^{4}	2.9×10^{-3}
**Photonic molecule**[5]	3600	NIR (1.55)	0.04	100	1	Y	75	144	0.028
**Programmable photonic circuits**[6]	7.03×10^{6}	NIR (1.55)	0.01	200	1	Y	350	7.03×10^{4}	1.42×10^{-5}
**Digital FTS**[7]	1.78×10^{6}	NIR (1.55)	0.2	20	1	Y	99	3.55×10^{5}	1.13×10^{-5}
**Ring-assisted FTS**[8]	NA	NIR (1.60)	0.47	90	1	Y	1835	/	/
**High-Q Ring**[9]	3.5×10^{5}	NIR (1.55)	0.005	10	10	Y	50.4	1.75×10^{3}	2.86×10^{-5}
**Micro-disk**[10]	1.25×10^{5}	NIR (1.55)	0.2	20	1	Y	160	2.51×10^{4}	1.59×10^{-4}
**Arrayed Waveguide Grating and Ring**[11]	9×10^{6}	NIR (1.55)	0.1	25.4	9	Y	35	9×10^{5}	2.82×10^{-6}
**FTS Array**[12]	NA	MIR (3.30)	12	60	12	N	/	/	/
**FTS Array**[13]	9.5×10^{7}	MIR (3.75)	2.7	51	42	N	/	2.57×10^{8}	5.37×10^{-7}
**Waveguide Coupler**[14]	2.7×10^{6}	MIR (3.85)	3	350	1	Y	NA	8.1×10^{6}	1.29×10^{-4}
**MPCW (This work)**	**~1680**	**MIR (2.00)**	**0.5**	**100**	**8**	**Y**	**45.6**	**840**	**0.06**

WB: waveband, RES: resolution, BW: bandwidth, CH: channel, WNP: wavelength-normalized power, RFP: resolution-footprint product, BFR: bandwidth-footprint ratio.

The wavelength-normalized power (WNP) is defined as:

$$\mathrm{WNP} = P_{\mathrm{working}} \times \frac{P_\pi(1550)}{P_\pi(\text{center wavelength})}.$$

Reference

1. Redding, B., Liew, S. F., Sarma, R. & Cao, H. Compact spectrometer based on a disordered photonic chip. *Nature Photon* **7**, 746–751 (2013).
2. Li, A. & Fainman, Y. On-chip spectrometers using stratified waveguide filters. *Nat Commun* **12**, 2704 (2021).
3. Zhang, J., Cheng, Z., Dong, J. & Zhang, X. Cascaded nanobeam spectrometer with high resolution and scalability. *Optica* **9**, 517 (2022).
4. Sun, C. *et al.* Broadband and High-Resolution Integrated Spectrometer Based on a Tunable FSR-Free Optical Filter Array. *ACS Photonics* **9**, 2973–2980 (2022).
5. Xu, H., Qin, Y., Hu, G. & Tsang, H. K. Breaking the resolution-bandwidth limit of chip-scale spectrometry by harnessing a dispersion-engineered photonic molecule. *Light Sci Appl* **12**, 64 (2023).
6. Yao, C. *et al.* Integrated reconstructive spectrometer with programmable photonic circuits. *Nat Commun* **14**, 6376 (2023).
7. Kita, D. M. *et al.* High-performance and scalable on-chip digital Fourier transform spectroscopy. *Nat Commun* **9**, 4405 (2018).
8. Zheng, S. N. *et al.* Microring resonator-assisted Fourier transform spectrometer with enhanced resolution and large bandwidth in single chip solution. *Nat Commun* **10**, 2349 (2019).
9. Zhang, L. *et al.* Ultrahigh-resolution on-chip spectrometer with silicon photonic resonators. *OEA* **5**, 210100–210100 (2022).
10. Sun, C. *et al.* Scalable On-Chip Microdisk Resonator Spectrometer. *Laser & Photonics Reviews* **17**, 2200792 (2023).
11. Zheng, S. *et al.* A Single-Chip Integrated Spectrometer via Tunable Microring Resonator Array. *IEEE Photonics J.* **11**, 1–9 (2019).

12. Heidari, E., Xu, X., Chung, C.-J. & Chen, R. T. On-chip Fourier transform spectrometer on silicon-on-sapphire. *Opt. Lett.* **44**, 2883 (2019).
13. Nedeljkovic, M. *et al.* Mid-Infrared Silicon-on-Insulator Fourier-Transform Spectrometer Chip. *IEEE Photon. Technol. Lett.* **28**, 528–531 (2016).
14. Qiao, Q. *et al.* MEMS-Enabled On-Chip Computational Mid-Infrared Spectrometer Using Silicon Photonics. *ACS Photonics* **9**, 2367–2377 (2022).
