# Supplementary material for: An efficient mid-infrared computational spectrometer based on synergistic microcavity-coupled photonic crystal waveguides
Source: Nat Commun. 2026 Jun 3;17:7143. doi: 10.1038/s41467-026-73934-z (PMC13396208; doi:10.1038/s41467-026-73934-z)
Supplement: Supplementary file 2 — Transparent Peer Review file [file 41467_2026_73934_MOESM2_ESM.pdf]

nature portfolio

Peer Review File

An efficient mid-infrared computational spectrometer based on synergistic microcavity-coupled photonic crystal waveguides

Corresponding Author: Professor Chengkuo Lee

This file contains all reviewer reports in order by version, followed by all author rebuttals in order by version.

Attachments originally included by the reviewers as part of their assessment can be found at the end of this file.

Version 0:

Reviewer comments:

Reviewer #1

(Remarks to the Author)
This manuscript reports an MIR on-chip spectrometer based on microcavity-coupled photonic crystal waveguides. The design leverages slow-light-enhanced thermo-optic tuning and an alternating optimization reconstruction algorithm to improve spectral fidelity and computational efficiency. The work is technically solid, experimentally validated, and well-situated in the context of integrated MIR photonics. The results represent a meaningful advance toward high-resolution, broadband, and compact MIR spectrometers. However, several issues require clarification or additional data before publication.

1) The performance comparison in Figs. 5f–5g is primarily qualitative. Please include quantitative metrics (resolution, bandwidth, footprint, power consumption) in a comparison table against representative state-of-the-art devices, citing specific references.

2) Since each MPCW unit is driven up to 60 mW, potential thermal crosstalk between adjacent units could affect sampling independence. Please provide either simulation or experimental quantification of the temperature field overlap or channel drift during tuning.

3) Discuss the long-term stability of the calibration matrix. How reproducible is the calibration after repeated heating cycles or under ambient temperature variations?

4) The reconstruction error is reported for single measurements (Figs. 5a–d). Please include statistical data (e.g., mean standard deviation) from multiple runs or different spectra to demonstrate reconstruction consistency and repeatability.

5) Please elaborate on how the optimization algorithm's computational complexity scales with matrix dimension or sampling density.

Reviewer #2

(Remarks to the Author)
Please see uploaded review document for comments.

Reviewer #3

(Remarks to the Author)
The authors present a mid-infrared on-chip computational spectrometer based on microcavity-coupled photonic crystal waveguides (MPCWs), featuring a tailored reconstruction algorithm and thermo-optic tuning. While the work demonstrates careful engineering and achieves competitive specifications (0.5 nm resolution over 100 nm bandwidth around 2 µm), I find that the study does not meet the high bar of novelty, broad interest, or conceptual advancement required for publication in

Nature Communications. I therefore recommend rejection in its current form. Below are my major concerns:
1.The core architecture—combining photonic crystal waveguides with side-coupled cavities and thermal tuning—has been extensively explored in the near-infrared regime. While the translation to the mid-infrared (MIR) is valuable, the manuscript does not introduce a fundamentally new operating principle or physical mechanism. The improvements (e.g., non-uniform FSR, slow-light enhanced tuning) appear as logical extensions of existing photonic crystal concepts rather than a transformative step. For Nature Communications, the work would need to demonstrate a more paradigm-shifting innovation or a breakthrough in performance far beyond the state-of-the-art.
2.The manuscript focuses heavily on device design and spectral reconstruction under controlled conditions but lacks a compelling application demonstration. MIR spectroscopy is motivated by strong molecular absorption for sensing and diagnostics; however, the authors only test the system with simple laser lines and an ASE source. There is no validation using actual MIR-relevant samples (e.g., chemical vapors, biomolecules) to showcase the claimed advantages for gas sensing, environmental monitoring, or biomedical diagnostics. Without such evidence, the practical impact remains speculative.
3.The performance comparison (Figs. 5f–5g) selectively highlights metrics where the device excels (e.g., footprint-related ratios) but overlooks other critical parameters such as absolute accuracy, dynamic range, or power consumption relative to prior MIR on-chip spectrometers. The claimed 0.5 nm resolution is demonstrated with a single laser peak but not rigorously validated across the entire band or under realistic noisy conditions (Fig. 3e only shows simulation). Additionally, the algorithm’s speed advantage, while notable, is not benchmarked against other recent advanced reconstruction methods beyond basic convex optimization.
4.The manuscript briefly mentions scalability to other wavelengths but does not address practical barriers to larger channel counts or broader bandwidths. The current design uses only 8 MPCW units and requires 328 calibration measurements—a process that may become cumbersome for mass fabrication or field deployment. Furthermore, the impact of fabrication variability on cavity resonances and band-edge uniformity is not quantified, nor is the long-term stability of the thermo-optic tuning under repeated cycling discussed. These aspects are critical for assessing the technology’s readiness for real-world use.
5.The spetra for reconstruction are relatively simple, only laser peaks and broad ASE spectrum.
6.The term “benchmarking” in the title is misleading, as the study does not provide a systematic comparison against a range of existing MIR spectrometer technologies.

Version 1:

Reviewer comments:

Reviewer #1

(Remarks to the Author)
My comments have been properly addressed and I don't have any further suggestions.

Reviewer #3

(Remarks to the Author)
I thank the authors for the additional discussions and experiments to validate the novelty and value of their work. It can be accepted.

Open Access This Peer Review File is licensed under a Creative Commons Attribution 4.0 International License, which permits use, sharing, adaptation, distribution and reproduction in any medium or format, as long as you give appropriate credit to the original author(s) and the source, provide a link to the Creative Commons license, and indicate if changes were made.
In cases where reviewers are anonymous, credit should be given to 'Anonymous Referee' and the source.
The images or other third party material in this Peer Review File are included in the article’s Creative Commons license, unless indicated otherwise in a credit line to the material. If material is not included in the article’s Creative Commons license and your intended use is not permitted by statutory regulation or exceeds the permitted use, you will need to obtain permission directly from the copyright holder.
To view a copy of this license, visit https://creativecommons.org/licenses/by/4.0/

The authors appreciate the insightful comments from the reviewers. We have addressed the reviewers' concerns by point-to-point reply below. The reviewers' questions are presented in black. Our responses are in blue, and changes made to the manuscript in response to the reviewers' comments are highlighted in red.

Reviewer 1:

This manuscript reports an MIR on-chip spectrometer based on microcavity-coupled photonic crystal waveguides. The design leverages slow-light-enhanced thermo-optic tuning and an alternating optimization reconstruction algorithm to improve spectral fidelity and computational efficiency. The work is technically solid, experimentally validated, and well-situated in the context of integrated MIR photonics. The results represent a meaningful advance toward high-resolution, broadband, and compact MIR spectrometers. However, several issues require clarification or additional data before publication.

We appreciate the reviewer's comments and have addressed the concern raised below.

Q1: The performance comparison in Figs. 5f-5g is primarily qualitative. Please include quantitative metrics (resolution, bandwidth, footprint, power consumption) in a comparison table against representative state-of-the-art devices, citing specific references.

Reply: The authors appreciate the reviewer's comments. The authors concluded the quantitative metrics (footprint, working band, resolution, bandwidth, channels, wavelength-normalized power, resolution-footprint product, and bandwidth-footprint ratio) in Supplementary Note 16.

Structure	Footprint (μm^2)	WB (μm)	RES (nm)	BW (nm)	CH	Active?	WNP (mW)	RFP ($nm{\cdot}\mu m^2$)	BFR ($nm{\cdot}\mu m^{-2}$)
**Random disorder scattering[1]**	1250	NIR (1.51)	0.75	25	25	N	/	5625	0.02
**Stratified waveguide filters[2]**	9100	NIR (1.55)	0.45	180	32	N	/	4095	0.0198
**Cascaded nanobeam[3]**	4500	NIR (1.55)	0.16	16	3	Y	30	720	3.6×10^{-3}
**Grating-assisted F-P cavity[4]**	3.6×10^4	NIR (1.50)	0.43	73.2	5	Y	121	1.84×10^4	2.9×10^{-3}
**Photonic molecule[5]**	3600	NIR (1.55)	0.04	100	1	Y	75	144	0.028
**Programmable photonic circuits[6]**	7.03×10^6	NIR (1.55)	0.01	200	1	Y	350	7.03×10^4	1.42×10^{-5}
**Digital FTS[7]**	1.78×10^6	NIR (1.55)	0.2	20	1	Y	99	3.55×10^5	1.13×10^{-5}
**Ring-assisted FTS[8]**	NA	NIR (1.60)	0.47	90	1	Y	1835	/	/
**High-Q Ring[9]**	3.5×10^5	NIR (1.55)	0.005	10	10	Y	50.4	1.75×10^3	2.86×10^{-5}
**Micro-disk[10]**	1.25×10^5	NIR (1.55)	0.2	20	1	Y	160	2.51×10^4	1.59×10^{-4}

**Arrayed Waveguide Grating and Ring**[11]	9×10^6	NIR (1.55)	0.1	25.4	9	Y	35	9×10^5	2.82×10^{-6}
**FTS Array**[12]	NA	MIR (3.30)	12	60	12	N	/	/	/
**FTS Array**[13]	9.5×10^7	MIR (3.75)	2.7	51	42	N	/	2.57×10^8	5.37×10^{-7}
**Waveguide Coupler**[14]	2.7×10^6	MIR (3.85)	3	350	1	Y	NA	8.1×10^6	1.29×10^{-4}
**MPCW (This work)**	**1680**	**MIR (2.00)**	**0.5**	**100**	**8**	**Y**	**45.6**	**840**	**0.06**

WB: waveband, RES: resolution, BW: bandwidth, CH: channel, WNP: wavelength-normalized power, RFP: resolution-footprint product, BFR: bandwidth-footprint ratio.

Changes made to the manuscript:

Supplementary Note 16

16. Comparison of On-chip Spectrometers

Structure	Footprint (μm^2)	WB (μm)	RES (nm)	BW (nm)	CH	Active?	WNP (mW)	RFP ($nm\cdot\mu m^2$)	BFR ($nm\cdot\mu m^{-2}$)
**Random disorder scattering**[1]	1250	NIR (1.51)	0.75	25	25	N	/	5625	0.02
**Stratified waveguide filters**[2]	9100	NIR (1.55)	0.45	180	32	N	/	4095	0.0198
**Cascaded nanobeam**[3]	4500	NIR (1.55)	0.16	16	3	Y	30	720	3.6×10^{-3}
**Grating-assisted F-P cavity**[4]	3.6×10^4	NIR (1.50)	0.43	73.2	5	Y	121	1.84×10^4	2.9×10^{-3}
**Photonic molecule**[5]	3600	NIR (1.55)	0.04	100	1	Y	75	144	0.028
**Programmable photonic circuits**[6]	7.03×10^6	NIR (1.55)	0.01	200	1	Y	350	7.03×10^4	1.42×10^{-5}
**Digital FTS**[7]	1.78×10^6	NIR (1.55)	0.2	20	1	Y	99	3.55×10^5	1.13×10^{-5}
**Ring-assisted FTS**[8]	NA	NIR (1.60)	0.47	90	1	Y	1835	/	/
**High-Q Ring**[9]	3.5×10^5	NIR (1.55)	0.005	10	10	Y	50.4	1.75×10^3	2.86×10^{-5}
**Micro-disk**[10]	1.25×10^5	NIR (1.55)	0.2	20	1	Y	160	2.51×10^4	1.59×10^{-4}
**Arrayed Waveguide Grating and Ring**[11]	9×10^6	NIR (1.55)	0.1	25.4	9	Y	35	9×10^5	2.82×10^{-6}

**FTS Array[12]**	NA	MIR (3.30)	12	60	12	N	/	/	/
**FTS Array[13]**	9.5×10^{7}	MIR (3.75)	2.7	51	42	N	/	2.57×10^{8}	5.37×10^{-7}
**Waveguide Coupler[14]**	2.7×10^{6}	MIR (3.85)	3	350	1	Y	NA	8.1×10^{6}	1.29×10^{-4}
**MPCW (This work)**	**1680**	**MIR (2.00)**	**0.5**	**100**	**8**	**Y**	**45.6**	**840**	**0.06**

WB: waveband, RES: resolution, BW: bandwidth, CH: channel, WNP: wavelength-normalized power, RFP: resolution-footprint product, BFR: bandwidth-footprint ratio.

The wavelength-normalized power (WNP) is defined as:

$$\mathrm{WNP} = P_{\mathrm{working}} \times \frac{P_{\pi}(1550)}{P_{\pi}(\mathrm{center\ wavelength})}$$

Reference

1. Redding, B., Liew, S. F., Sarma, R. & Cao, H. Compact spectrometer based on a disordered photonic chip. *Nature Photon* **7**, 746–751 (2013).
2. Li, A. & Fainman, Y. On-chip spectrometers using stratified waveguide filters. *Nat Commun* **12**, 2704 (2021).
3. Zhang, J., Cheng, Z., Dong, J. & Zhang, X. Cascaded nanobeam spectrometer with high resolution and scalability. *Optica* **9**, 517 (2022).
4. Sun, C. *et al.* Broadband and High-Resolution Integrated Spectrometer Based on a Tunable FSR-Free Optical Filter Array. *ACS Photonics* **9**, 2973–2980 (2022).
5. Xu, H., Qin, Y., Hu, G. & Tsang, H. K. Breaking the resolution-bandwidth limit of chip-scale spectrometry by harnessing a dispersion-engineered photonic molecule. *Light Sci Appl* **12**, 64 (2023).
6. Yao, C. *et al.* Integrated reconstructive spectrometer with programmable photonic circuits. *Nat Commun* **14**, 6376 (2023).
7. Kita, D. M. *et al.* High-performance and scalable on-chip digital Fourier transform spectroscopy. *Nat Commun* **9**, 4405 (2018).
8. Zheng, S. N. *et al.* Microring resonator-assisted Fourier transform spectrometer with enhanced resolution and large bandwidth in single chip solution. *Nat Commun* **10**, 2349 (2019).
9. Zhang, L. *et al.* Ultrahigh-resolution on-chip spectrometer with silicon photonic resonators. *OEA* **5**, 210100–210100 (2022).
10. Sun, C. *et al.* Scalable On-Chip Microdisk Resonator Spectrometer. *Laser & Photonics Reviews* **17**, 2200792 (2023).
11. Zheng, S. *et al.* A Single-Chip Integrated Spectrometer via Tunable Microring Resonator Array. *IEEE Photonics J.* **11**, 1–9 (2019).
12. Heidari, E., Xu, X., Chung, C.-J. & Chen, R. T. On-chip Fourier transform spectrometer on silicon-on-sapphire. *Opt. Lett.* **44**, 2883 (2019).
13. Nedeljkovic, M. *et al.* Mid-Infrared Silicon-on-Insulator Fourier-Transform Spectrometer Chip. *IEEE Photon. Technol. Lett.* **28**, 528–531 (2016).
14. Qiao, Q. *et al.* MEMS-Enabled On-Chip Computational Mid-Infrared Spectrometer Using Silicon Photonics. *ACS Photonics* **9**, 2367–2377 (2022).

Q2: Since each MPCW unit is driven up to 60 mW, potential thermal crosstalk between adjacent units could affect sampling independence. Please provide either simulation or experimental quantification of the temperature field overlap or channel drift during tuning.

Reply: The authors appreciate the reviewer's comments. **We would like to clarify that the current experimental setup employs a single detector and only one MPCW channel is heated at a time during the experiment, each channel is not affected by thermal crosstalk from other channels during measurement.** However, the thermal crosstalk concerns raised by the reviewer would have an impact on potential multi-channel detection schemes. To address this concern, we evaluated the influence of inter-channel thermal crosstalk on the response spectra of our MPCW units. To this end, we performed both simulations and experiments.

In the experiments, we first used a TEC to regulate the chip at different background temperatures and measured the corresponding variations of the response spectra in Fig. **R1a** and **R1b**. Then, we calculated the spectral variation $v(T)$ caused by ambient temperature variations, as shown in Fig. **R1c**, which is defined as

$$v(T) = \frac{\|R(T) - R(T_0)\|_2}{\|R(T_0)\|_2},$$

where $R(T)$ denotes the filter response spectrum at temperature T, and T_0 is the reference temperature (fixed at 23 °C here). Subsequently, when applying 60 mW of heating power to the 6th MPCW channel, the 5th MPCW channel exhibited a measurable disturbance, with a calculated relative disturbance of approximately 11.1%. Combined with our temperature calibration (Fig. **R2**), this 11.1% relative disturbance corresponds to a temperature variation of 1.89 K, which is consistent with the 10.1% deviation caused by a 1.72 K temperature variation in our simulations (Fig. **R3a**). Therefore, for multi-channel measurement schemes, our proposed solution is to etch air trenches between adjacent channels (Fig. **R3b**). It can be confirmed that with air trenches, the induced temperature variation is only 0.04 K, corresponding to a relative deviation of 0.4%. Such a 0.4% relative deviation is acceptable in practice.

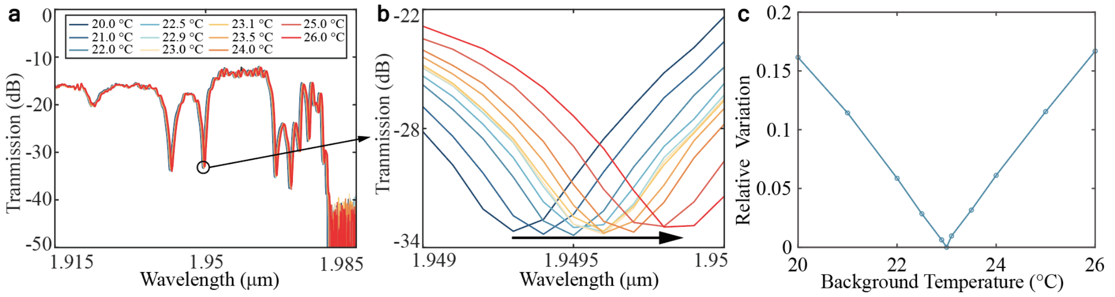

Fig. R1 | a Response of the 6th MPCW channel as a function of temperature **b** Magnified view of the transmission response **c** Relative spectral variation with reference to 23 °C.

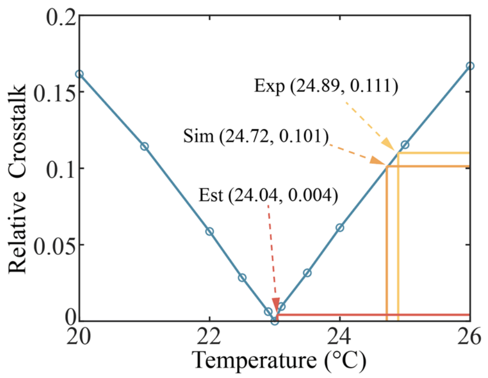

Fig. R2 | Estimation of temperature perturbation after using air trenches based on simulation and experimental results.

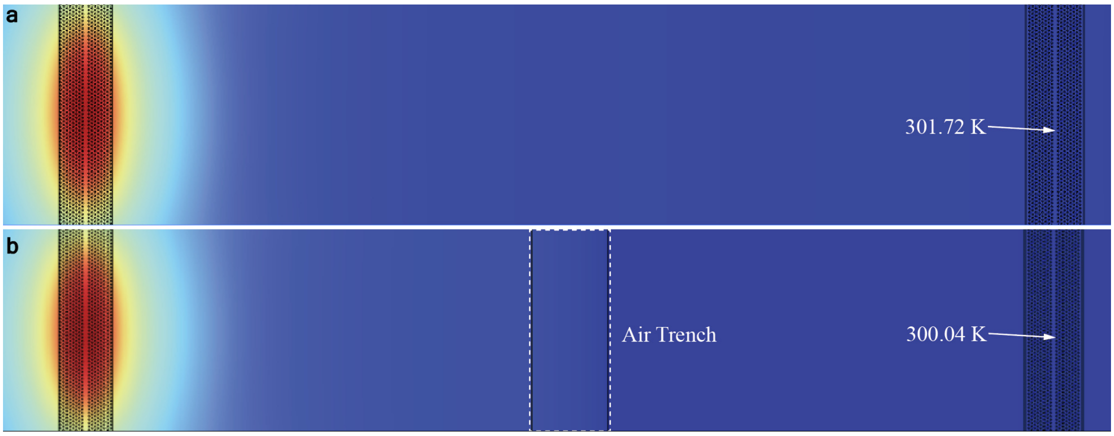

Fig. R3 | Simulation results of heating perturbation **a** with and **b** without air trenches.

Changes made to the manuscript:

Supplementary Note 8

8. Temperature Perturbation

In our experiments, the spectrometer operates at room temperature, and we set the operating temperature to 23 °C. To evaluate the influence of temperature on the spectrometer, we measured the variation in the transmission characteristics of the filter over the temperature range from 20 °C to 26 °C (20.0, 21.0, 22.5, 22.9, 23.0, 23.1, 23.5, 24.0, 25.0, and 26.0 °C). The measurement results are shown in Fig. **S14a** and Fig. **S14b**. The transmission spectrum of the filter exhibits a red shift as the temperature increases. This phenomenon is attributed to the refractive index of silicon increases with rising temperature, which shifts the band-edge position of the photonic crystal and consequently leads to the spectral drift of the filter.

To quantify the specific impact of temperature perturbation on the transmission spectrum of the filter, we used the relative variation $v(T)$ to characterize the change in its transmission behavior, defined explicitly as:

$$v(T) = \frac{\|R(T) - R(T_0)\|_2}{\|R(T_0)\|_2}$$

where $R(T)$ denotes the filter response spectrum at temperature T, and T_0 is the reference temperature (fixed at 23 °C here). Calculating the relative variation at different temperatures allows a clearer observation of the temperature effect on the filter transmission. The calculated results are displayed in Fig. **S14c**. It can be seen that the transmission characteristics of the filter change noticeably with increasing temperature, especially near the operating wavelength of the filter, where

the variation is more significant. This indicates that ambient temperature fluctuations in non-laboratory environments can considerably affect the filter performance and highlights the necessity of integrating a TEC for precise thermal stabilization in practical applications.

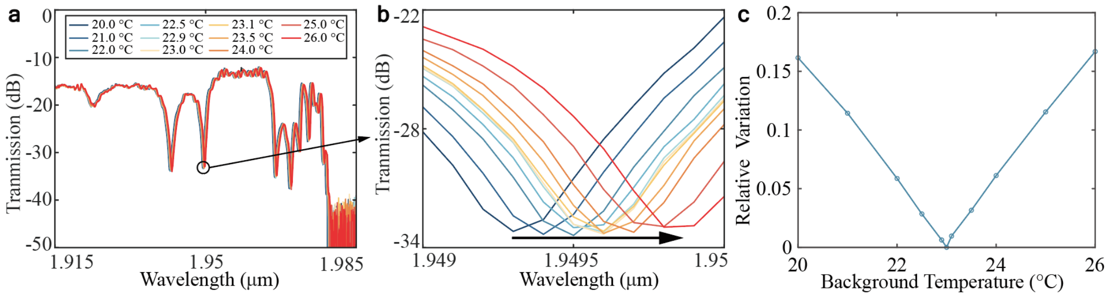

Fig. **S14**. **a** Response of the 6th MPCW channel as a function of temperature. **b** Magnified view of the transmission response **c** Relative spectral variation with reference to 23 °C.

Supplementary Note 9

9. Thermal Crosstalk

As the MPCW spectrometer contains multiple filter channels closely spaced, applying thermal power to a channel can affect the transmission characteristics of adjacent channels. Although our design is intended for single-channel operation, the measurements were conducted by sequentially heating and reading individual channels. The simultaneous excitation of multiple channels with multiple detectors would introduce additional thermal crosstalk.

To evaluate the effect of thermal crosstalk, we heated one channel and measured the transmission characteristics of its neighboring MPCW channels. The experimental results show that heating one channel alters the transmission spectrum of adjacent channels, with relative spectral deviation of 11.1% compared with the unheated condition. Such thermal perturbation can be mitigated by introducing air isolation region (air trench), as illustrated by our simulation results (Fig. **S15**). Without an isolation region, heat conducts directly through the silicon slab to neighboring channels, raising their temperature by approximately 1.72 K and perturbing their response spectra (10.1%), in good agreement with our experimental observations (Fig. **S15a**). With air isolation regions, heat conduction is effectively suppressed, reducing the temperature variation of adjacent channels to only 0.04 K (Fig. **S15b**), corresponding to a relative crosstalk of 0.4% (Fig. **S16**). This significantly alleviates thermal crosstalk.

Therefore, in multi-channel detection applications, deeply etched air isolation trenches can be adopted during the design and fabrication process to suppress thermal crosstalk and improve the stability and reliability of multi-channel spectral measurements.

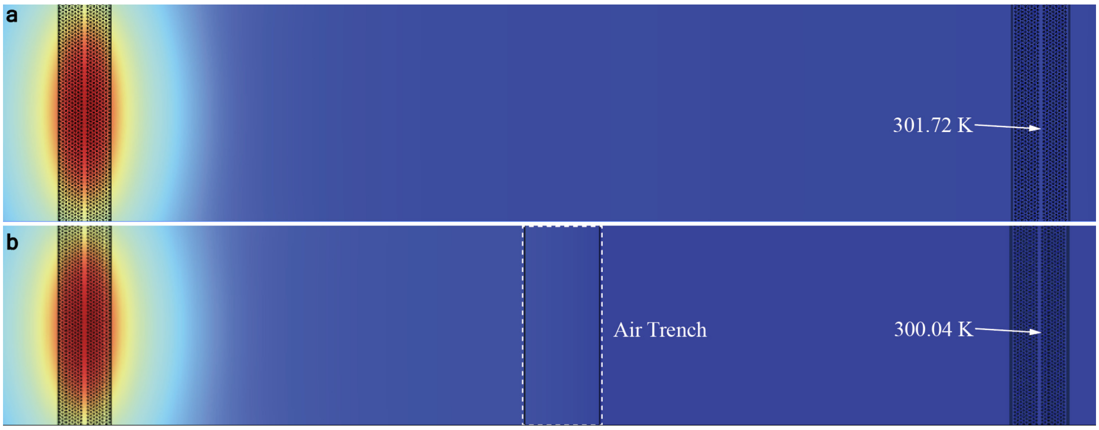

Fig. **S15**. Simulation results of heating perturbation **a** with and **b** without air trenches.

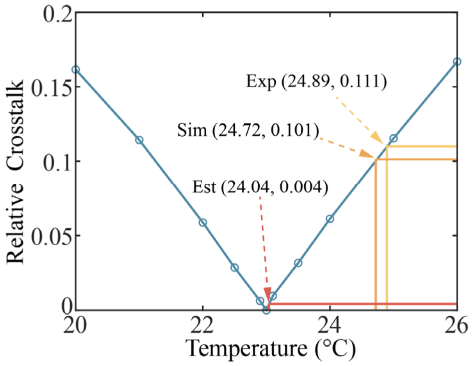

Fig. **S16**. Estimation of temperature perturbation after using air trenches based on simulation and experimental result

Q3: Discuss the long-term stability of the calibration matrix. How reproducible is the calibration after repeated heating cycles or under ambient temperature variations?

Reply: The authors appreciate the reviewer's comments. As requested, we evaluated the stability of the response spectrum under constant heating power (0 mW and 60 mW), yielding an average relative fluctuation of approximately 0.9% (Fig. **R4a**). Similar to temperature-induced variations, the average spectrum fluctuation is defined as:

$$f(t) = \frac{\left\| R(t) - R_{average} \right\|_2}{\left\| R_{average} \right\|_2}$$

where $R(t)$ is the response spectrum acquired at time t, and $R_{average}$ is the average response spectrum taken at different times. In addition, following your suggestion, we performed four cycles of calibration matrix measurements at 30-minute intervals. The results in Fig. **R4b** demonstrate that when the background temperature is stable, the average relative fluctuation of the calibration matrix is about 0.7%, which can be attributed to the precision limit of TEC controller.

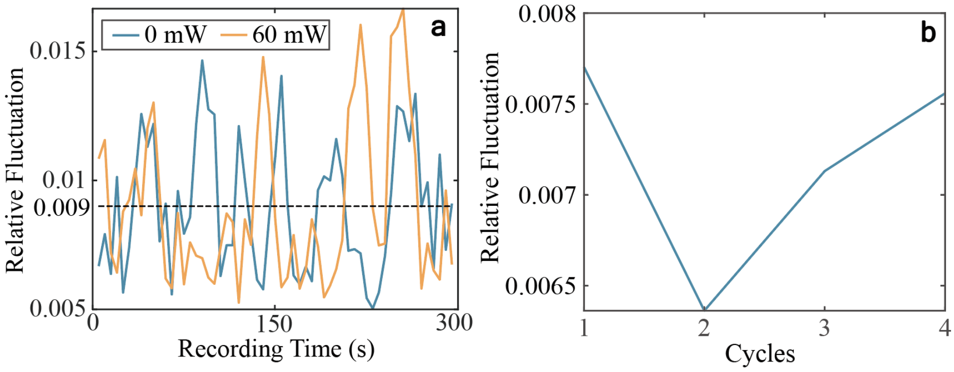

Fig. R4 | a Fluctuations in the response spectra during long-term measurements at a specific heating power **b** Fluctuations in the calibration matrix across multiple measurement cycles.

Changes made to the manuscript:

Supplementary Note 10

10. Operation Repeatability

For the operational repeatability, we first measured the average relative fluctuation of the spectra of the MPCW element at 0 mW and 60 mW (with the TEC base temperature set to 23 °C). Similar to temperature-induced variations, the average spectrum fluctuation is defined as:

$$f(t) = \frac{\|R(t) - R_{average}\|_2}{\|R_{average}\|_2}$$

where $R(t)$ is the response spectrum acquired at time t, and $R_{average}$ is the average response spectrum taken at different times. As shown in Fig. **S17a**, the average relative fluctuation of the spectrum is approximately 0.9%, with the maximum fluctuation not exceeding 1.6%. These fluctuations are primarily attributed to the precision limit of TEC controller.

Furthermore, we performed four complete measurements of the response matrix at 30-minute intervals. In each measurement cycle, the filters underwent a full heating and cooling cycle. We calculated the relative variation of the average transmission characteristics for each channel across different measurement rounds. As shown in Fig. **S17b**, the average relative fluctuation of the entire response matrix is roughly 0.7%. This demonstrates that the designed thermo-optically controlled photonic crystal filters maintain high operational repeatability across multiple thermal tuning cycles.

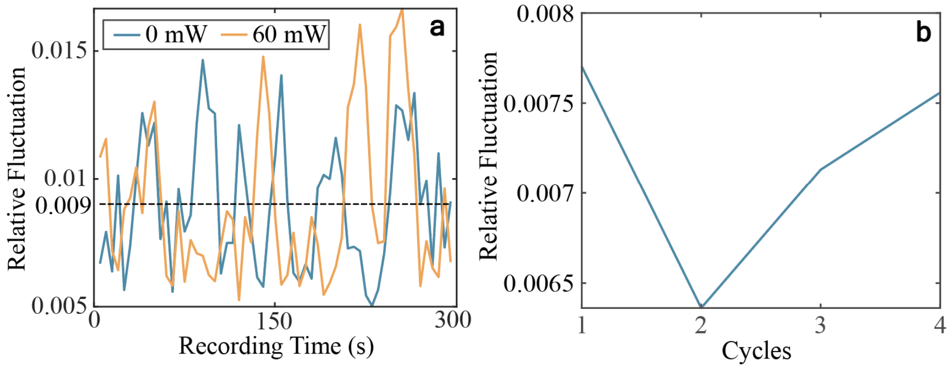

Fig. **S17**. **a** Fluctuations in the response spectra during long-term measurements at a specific heating power. **b** Fluctuations in the calibration matrix across multiple measurement cycles.

Q4: The reconstruction error is reported for single measurements (Figs. 5a–d). Please include statistical data (e.g., mean standard deviation) from multiple runs or different spectra to demonstrate

reconstruction consistency and repeatability.

Reply: The authors appreciate the reviewer's comments. Following your suggestion, we resampled each of the five original spectral cases five times, conducted spectral reconstructions based on the resampled data, and calculated the mean $\varepsilon_{\mathrm{mean}}$ and standard deviation $\varepsilon_{\mathrm{STD}}$ of the relative errors of the reconstructed outcomes (Fig. **R5**).

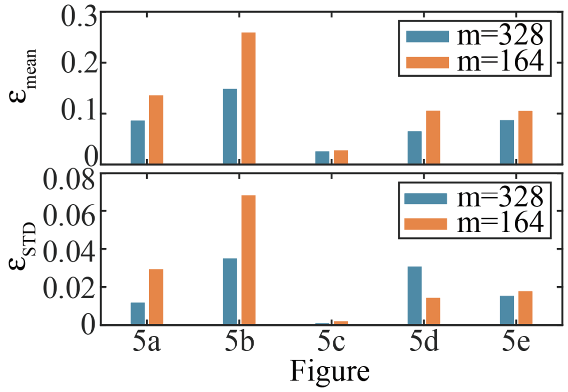

Fig. R5 | The mean $\varepsilon_{\mathrm{mean}}$ and standard deviation $\varepsilon_{\mathrm{STD}}$ of reconstruction error based on Fig. 5 presented in the main text.

Changes made to the manuscript:

Supplementary Note 14

14. Reconstruction Consistency

To evaluate the reliability of the results, we performed an additional five sampling reconstructions for each case in the main text and calculated the mean $\varepsilon_{\mathrm{mean}}$ and standard deviation $\varepsilon_{\mathrm{STD}}$ of the reconstruction errors across these five independent reconstructions. In addition to our standard sampling (m = 328), we also adopted an under-sampling scheme for spectral reconstruction (m = 164). As illustrated in Fig. **S21**, $\varepsilon_{\mathrm{mean}}$ of narrow-band spectra (5a, 5b) are more sensitive to the number of channels, and finer sampling results in lower narrow-band reconstruction errors. In contrast, the reconstruction errors of broadband spectra (5c) are less sensitive to the number of sampling channels.

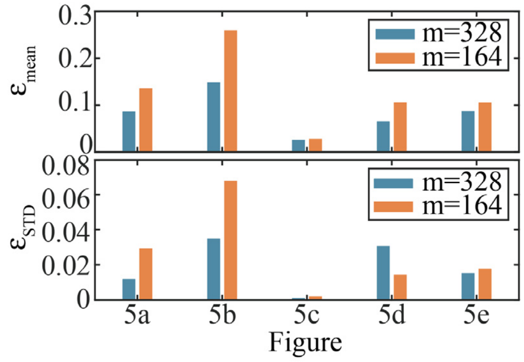

Fig. S21. The mean $\varepsilon_{\mathrm{mean}}$ and standard deviation $\varepsilon_{\mathrm{STD}}$ of reconstruction error based on Fig. 5 presented in the main text.

Q5: Please elaborate on how the optimization algorithm's computational complexity scales with matrix dimension or sampling density.

Reply: The authors appreciate the reviewer's comments. We thank you for pointing out a potentially confusing aspect of our algorithm, and we have restructured the relevant section accordingly. First,

regarding the computational complexity, we would like to clarify that the reconstruction time is influenced not only by the sampling density but also by the number of channels in the reconstructed spectrum. That is, when both the sampling density and the number of spectral channels increase, the computational cost of the optimization algorithm rises. We apologize for the confusion. The figure only displayed the product of the matrix dimensions (m*n), which may have led to a misunderstanding. We have revised the figure (Fig. **R6**) to include more granular details." Furthermore, in our optimization objective, the smoothness term and the sparsity term are decoupled, which means two variables need to be optimized simultaneously. Due to the non-smooth nature of L1 regularization, the optimization algorithm converts the L1 regularization into an equivalent formulation, leading to higher computational complexity and reduced computational efficiency in large-scale computations.

Concerning the comparison methodology, the initial version relied on a 10-fold cross-validation (CV) method, which is computationally intensive due to the repeated runs (about 1000 runs) required for parameter tuning. Moreover, grid-based search is inefficient for finding the optimal parameter. In contrast, our subspace iteration method adopts generalized cross-validation (GCV), which avoids repeated computations under different regularization parameters. While several studies have proposed using K-fold CV for parameter selection, comparing the conventional optimization method using K-fold CV against our subspace iteration method with GCV-based selection may not provide a consistent benchmark due to the vastly different computational requirements. To address this, we introduce a GCV function inspired by the subspace iteration method to estimate the optimal regularization parameter, significantly reducing the search time. Additionally, since CVXPY supports different solvers, the computational time varies across algorithms. We therefore re-compared two different solvers: CVXOPT and MOSEK. Comparatively, the MOSEK solver combined with the GCV method in the optimization framework yields better performance. However, the conventional optimization method for solving the minimization problem relies on gradient-based approaches. Consequently, the computational time increases significantly when both the sampling density and the number of spectral channels grow. In addition, as can be seen from the GCV function formula

$$G(\alpha,\beta) = \frac{\|\mathbf{r}(\alpha,\beta)\|_2^2}{\left(trace(\mathbf{I} - \mathbf{T}(\mathbf{T}^\dagger\mathbf{T} + \alpha^2\,\mathbf{D}^\dagger\,\mathbf{D} + \beta^2\,\mathbf{F}^\dagger\,\mathbf{F})^{-1}\mathbf{T}^\dagger)\right)^2},$$

the hat matrix $\mathbf{T}\left(\mathbf{T}^\dagger\mathbf{T} + \alpha^2\,\mathbf{D}^\dagger\,\mathbf{D} + \beta^2\,\mathbf{F}^\dagger\,\mathbf{F}\right)^{-1}\mathbf{T}^\dagger$ varies with different regularization parameters, so the computation time increases notably with matrix size, corresponding to a computational burden of minimizing the GCV function. By contrast, the subspace-based method for least-squares problems computes the projection solution within a low-dimensional subspace, resulting in much lower computational overhead than conventional optimization methods for large-scale problems. Moreover, the subspace method evaluates the GCV function in low dimensions during iteration, so the computational cost of the GCV value is also relatively low.

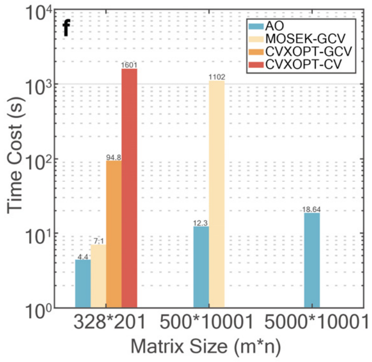

Fig. R6 | Reconstruction efficiency of different methods for different matrix dimensions. For larger-scale problems, the reconstruction time of our alternating optimization does not increase significantly. Direct solution via convex optimization was implemented using CVXPY (solver: CVXOPT and MOSEK), while the alternating optimization method was carried out with the IRtools. Calculations were all performed on a laptop with a 10-core Apple M1 Pro CPU and 16 GB of RAM. AO: alternating optimization; MOSEK-GCV: convex optimization with MOSEK backend with GCV; CVXOPT-GCV: convex optimization with CVXOPT backend with GCV; CVXOPT-CV: convex optimization with CVXOPT backend with 10-fold-CV.

Changes made to the manuscript:

Additionally, we compared the average reconstruction speed of different methods (Fig. 5f) using the test cases presented in Figs. 5a-5e. Furthermore, we also examined the computation time of these methods for larger-scale calibration matrices. The time shown in Fig. 5f includes the parameter search duration. These methods include the direct solution [Eq. (3)] via convex optimization (using CVXOPT and MOSEK solvers) which searches for regularization parameters through K-fold CV and GCV methods (K=10, see Supplementary Note 15 for details on CV and GCV), and the alternating optimization method with automatic parameter selection. For convex optimization, it is worth noting that parameter searching in CV is time-consuming. Reducing the search time may lead to failing to identify the optimal regularization parameters, which in turn results in larger reconstruction errors compared to the alternating optimization method. The GCV methods in convex optimization perform well when the matrix size is small. However, as the size of the calibration matrix increases, the overhead of the optimization process and the computational cost of the GCV function calculation increases significantly, leading to an increase in the overall computation time. For alternating optimization, since the problem is solved within the projected subspace, the computation time does not increase significantly. Moreover, since the GCV value is computed within subspace, the computational burden is significantly reduced.

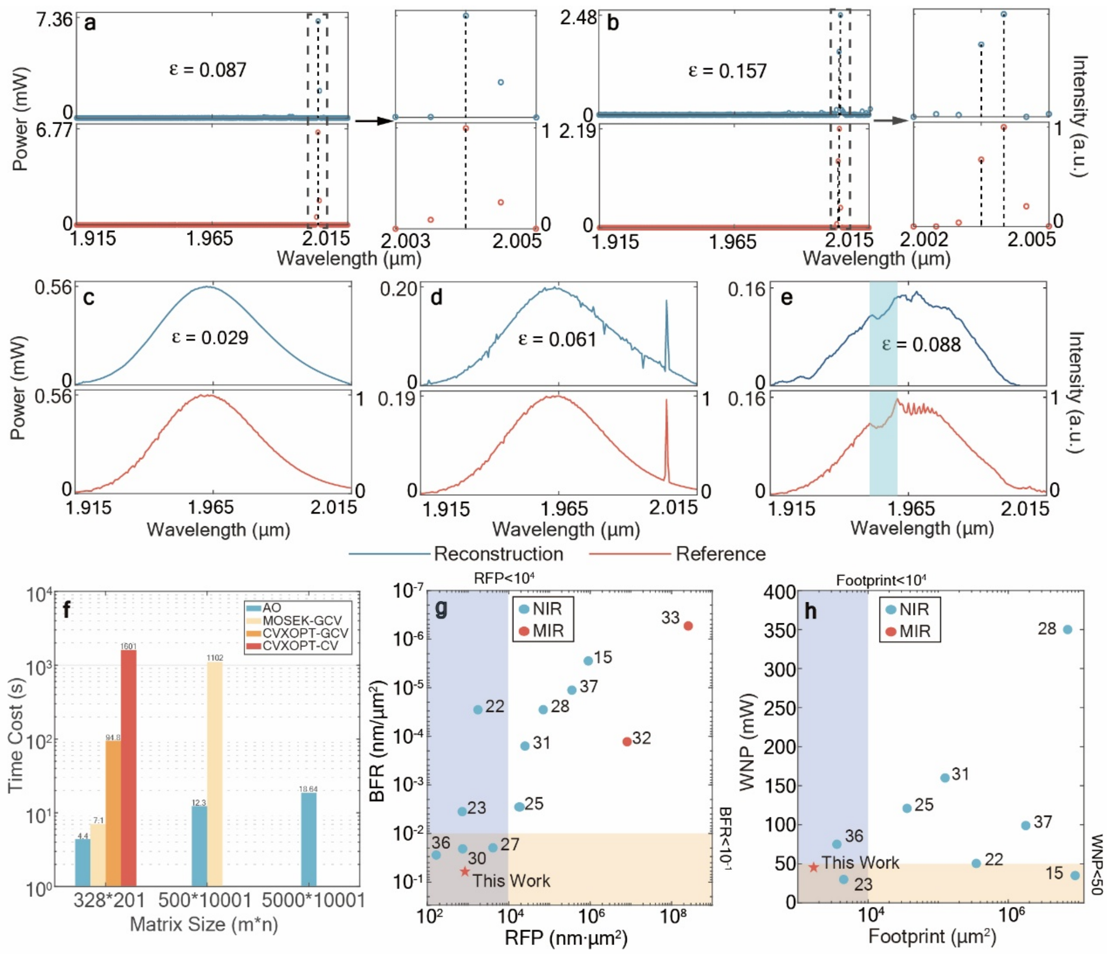

Fig. 5 | Reconstruction results and performance comparison. a-e The blue line is the algorithm reconstruction result, and the red line is the reference measured by the commercial spectrometer. **a** Single peak input spectrum and its reconstructed result. The peak is at 2004 nm. The right side shows an enlargement of the left side, which exhibits a full width at half maximum (FWHM) of 0.5 nm. **b** Dual peaks reconstructed result. An additional peak is set at 2003.5 nm adjacent to the 2004 nm peak. The right side displays a zoomed-in view of the left side. Compared to the **a**, the reconstruction result displays the additional peak line. **c** Broadband ASE input spectrum and its reconstructed result. **d** The input hybrid spectrum, which combines the ASE and a laser at 2004 nm. **e** Spectrum of ASE after absorption by carbon dioxide and its reconstruction result. The blue region represents the reconstructed absorption envelope. **f** Reconstruction efficiency of different methods for different matrix dimensions. For larger-scale problems, the reconstruction time of our alternating optimization does not increase significantly. Direct solution via convex optimization was implemented using CVXPY (solver: CVXOPT and MOSEK), while the alternating optimization method was carried out with the IRtools. Calculations were all performed on a laptop with a 10-core Apple M1 Pro CPU and 16 GB of RAM. AO: alternating optimization; MOSEK-GCV: convex optimization with MOSEK backend with GCV; CVXOPT-GCV: convex optimization with CVXOPT backend with GCV; CVXOPT-CV: convex optimization with CVXOPT backend with 10-fold-CV.The spectrometer performance is compared with the focus on **g** resolution-footprint product (RFP) and bandwidth-footprint ratio (BFR), **h** wavelength-normalized-power (WNP) and footprint.

Supplementary Note 15

15. Selection of Regularization Parameters in Convex Optimization.

In the spectral reconstruction process of a computational spectrometer, cross-validation (CV) primarily partitions the detection channels of the calibration matrix (i.e., the rows of the matrix) into a reconstruction part (rp) and a validation part (vp). Spectral reconstruction is performed using the calibration results and detected power of the reconstruction part channels ($\mathbf{T}_{rp}$ and $\mathbf{d}_{rp}$) and evaluates the quality of regularization parameters by comparing the error between the predicted power ($\mathbf{T}_{vp}\hat{\mathbf{s}}_{rp}$), obtained by applying the reconstructed spectrum ($\hat{\mathbf{s}}_{rp}$) to the validation part channels, and the measured power $\mathbf{d}_{vp}$ of the validation part. This error can be expressed as:

$$\frac{1}{N_{vp}}\sum_{vp}\left(\mathbf{T}_{vp}\hat{\mathbf{s}}_{rp}-\mathbf{d}_{vp}\right)^2$$

N_{rp} and N_{vp} denote the number of channels in the reconstruction part and validation part, respectively.

The indices of all channels are shuffled, then partitioned into ten groups, each of which serves as the validation part in turn. For each validation group, the indices not included in the validation part form the reconstruction part. For each regularization parameter, the CV error of each group is calculated, and the optimal parameters are selected as those corresponding to the minimum CV error. In practical implementation, we noticed that the computational time required for K-fold CV is excessively long. Additionally, we introduce a generalized cross-validation (GCV) function for selecting regularization parameters, which references the GCV function used in the subspace iteration method, though this GCV function is approximate for L1 regularization parameter selection. The GCV function is defined as follows:

$$G(\alpha,\beta)=\frac{\|\mathbf{r}(\alpha,\beta)\|_2^2}{\left(trace(\mathbf{I}-\mathbf{T}(\mathbf{T}^{\dagger}\mathbf{T}+\alpha^2\,\mathbf{D}^{\dagger}\,\mathbf{D}+\beta^2\,\mathbf{F}^{\dagger}\,\mathbf{F})^{-1}\mathbf{T}^{\dagger})\right)^2}$$

where $\mathbf{r}(\alpha,\beta)$ denotes the residual at regularization parameters α and β. The matrix $\mathbf{F}$ can be expressed as:

$$\mathbf{F}=\mathrm{diag}\left(\left[2\sqrt{s_i^2+\varepsilon}\right]^{-\frac{1}{2}}\right)_{i=1}^{n}$$

The optimization procedure for the GCV method is as follows: initialize α and β, perform one round of solution using convex optimization tools, minimize the GCV function with the obtained solution, update α and β, and repeat this process until the GCV values reach a plateau.

Reviewer 2:

The mid-infrared (MIR) waveband contains a vast number of vibrational absorption signatures for a range of applications. MIR spectroscopy is an important technique to identify these signatures. However, there is a growing demand to shrink the existing bulky and expensive systems (i.e. FTIR /otherwise). Integrated photonic approaches to realise miniaturized MIR spectroscopy are promising, but numerous roadblocks exist including materials transparency limitations, sensitivity (sensing architectures), optical throughputs, necessity for long optical path lengths (i.e. mimicking FTIR), narrow bandwidths etc. etc. In this work, the authors present quite a novel miniaturized MIR computational spectrometer prototype based on microcavity-coupled photonic crystal waveguide (MPCW) architecture. The metrics are a 100 nm bandwidth with 0.5 nm resolution (at approx. 2 um), spectrum acquisition of 4 seconds and footprint. This is achieved using an array of tapered PCWs, thermal tuning and computational reconstruction techniques. The architecture is novel, and performance is impressive compared to the state-of-the-art in on-chip spectrometers. Comparison to commercial systems is lacking, calibration and stability requirements are demanding, susceptibility to error is large, bandwidth is small (and debatable as to how scalable this is) and there are other numerous drawbacks (mentioned below). Nonetheless, I would recommend to accept the work after addressing the changes / suggestions below. The points and questions I would like to addressed are as follows, and are listed in no particular order:

We appreciate the reviewer's comments and have addressed the concern raised below.

Q1: Authors state: "scalability of our MPCWs enables easy extension of the operational range to other wavelength bands". Si-SiO2 is opaque beyond approx 4um (specifically the SiO2), therefore how is it expected to scale this to longer wavelengths? Are you fundamentally limited by your design to shorter wavebands? Also, to evidence this, the authors (in Fig S.2), have a band edge wavelength v lattice constant plot, however this doesn't indicate the power loss associated with moving towards 3.5 um due to the increased extinction of the materials. Further, on the topic of scalability: how would this system be practically translated to real world applications? As in it's current form it is a world away from realization in any demanding environment.

Reply: The authors appreciate the reviewer's comments. The issue you raised is crucial: silica exhibits extremely strong absorption beyond a wavelength of 4 μm. Regarding the first issue, the primary solution is to replace the chip platform. One feasible solution is to utilize the silicon-on-sapphire (SOS) wafer. Since sapphire's absorption occurs above 5 μm, according to our previous experimental results [*Opt. Express,* **23**, 6965–6975 (2015)] and prior study [*Appl. Phys. Lett.* **97**, 213501 (2010)], this platform can readily satisfy the propagation requirements within the 1–5 μm range; For photonic crystal structures, when supported by an appropriate material platform, their spectral characteristics is determined by the lattice constant and hole size. For shorter wavelengths, the lattice constant can generally be scaled accordingly for the target waveband, and the structure operates properly at 1310 nm. For even shorter wavelengths, the performance is limited by the absorption of short-wave light in silicon.

Regarding the second issue, we have existing measured data to support our conclusions. We previously fabricated photonic crystal waveguides on a 340 nm SOI wafer. With a 50% duty cycle, the lattice constant of photonic crystal waveguide supporting propagation near 3.5 μm is approximately 1.02 μm. Here, we present our measured results normalized to a reference grating (Fig. **R7b**). The experiment was conducted using a Daylight Solutions Sidekick laser (with a

wavelength range of 3.3–3.7 μm) and a Yokogawa AQ6377 spectrometer to measure the response spectrum of the photonic crystal waveguide, achieving an insertion loss of 3.1 dB, which enables operation in the 3 μm waveband. The response spectrum in Fig. **R7b** demonstrates the scalability, which can be achieved by shifting the bandgap and band edges to different wavelengths.

In addition, a power splitter was used in our experiment. For practical applications, on-chip optical switch arrays and wavelength division multiplexing (WDM) can be designed to route light into different MPCW units to achieve the desired functionality.

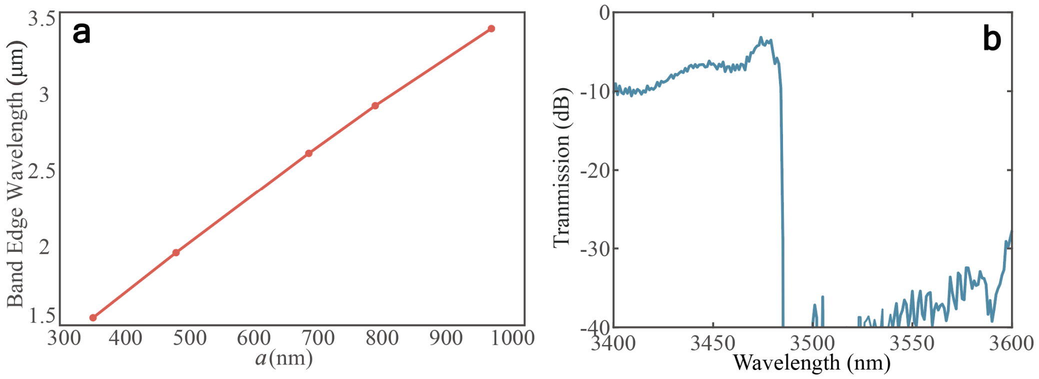

Fig. R7 | a The relationship between band edge wavelength and a. **b** Experimental verification of wavelength scalability near 3.5 μm region**.**

Changes made to the manuscript:

Supplementary Note 2

2. Microcavities and Scalability Analysis

Fig. **S2a** illustrates the relationship between band edge wavelengths (ranging from 1.5 μm to 3.5 μm) and lattice constant a, further demonstrating the scalability of our MPCW spectrometer. In addition, a PCW operating at the 3.5 μm waveband with a 1.02 μm lattice constant and a 50% duty cycle was fabricated on a 340 nm SOI wafer. The experiment was conducted using a Daylight Solutions Sidekick laser (with a wavelength range of 3.3–3.7 μm) and a Yokogawa AQ6377 spectrometer to measure the response spectrum of the PCW (Fig. **S2b**, normalized to a reference grating), achieving an insertion loss of 3.1 dB, which enables operation in the 3.5 μm waveband. The response spectrum in the figure can verify the scalability, which can be realized by shifting the bandgap and band edges to different wavelengths.

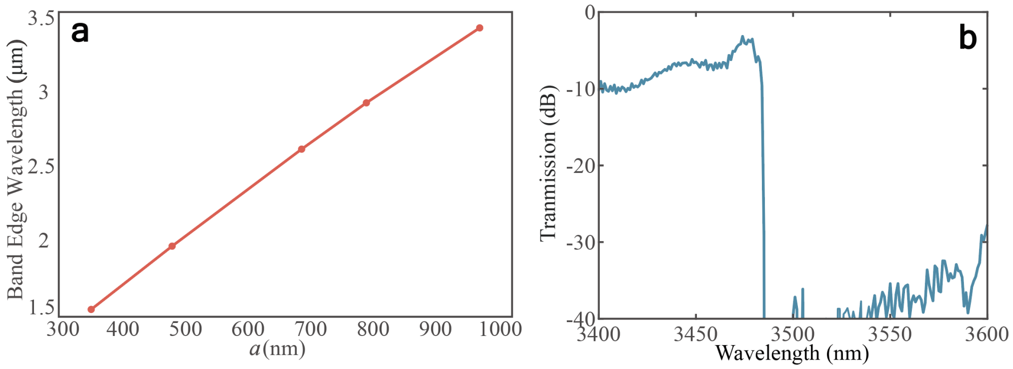

Fig. **S2**. **a** The relationship between band edge wavelength and a. **b** Experimental verification of wavelength scalability near 3.5 μm region.

Q2: Bandwidth limitations: is there still a fundamental limitation to scaling beyond the 100 nm

bandwidth presented? If so, this would mean again spectrometers for specific gases / analytes and not a broadband spectrometer (i.e. FTIR)

Reply: The authors appreciate the reviewer's comments. **Regarding the broadband application requirements you mentioned, it should be clarified that achieving an operating bandwidth on the order of 1 µm directly for a single set of silicon photonic devices is challenging**. The core challenging stems from two aspects: first, the Si material itself has a high refractive index, which induces significant material dispersion; second, the modal dispersion exists in the on-chip waveguide structure. Together, these two factors restrict the wavelength coverage of a single set of devices. **However, these do not represent fundamental limitations. To achieve an ultra-wide operating bandwidth (e.g., the 1.4 µm scale mentioned by the reviewer), it is necessary to integrate multiple sets of functional devices, including optical couplers and beam splitters adapted to different wavebands, and expand the bandwidth through multi-band splicing**. The operating bandwidth achieved by current on-chip spectrometers based on photonic devices is on the order of several hundred nanometers. Therefore, the "broadband" mentioned in this paper refers to a performance advantage relative to other reported on-chip spectrometers. Compared with the bandwidth of tens of nanometers for most on-chip spectrometers, the 100 nm operating range achieved by the scheme in this paper already has significant broadband detection capabilities. However, it should be clarified that its bandwidth magnitude is still different from that of conventional broadband spectrometers such as Fourier transform infrared spectrometers (FTIR), and the two are suitable for different application scenarios.

The title of the paper and the SI are different. The title of this paper should not have the word benchmarking in, as it doesn't make sense here. Also, please clarify the actual paper title.

Reply: The authors thank the reviewer's correction. In light of the reviewer's insightful suggestions and the core contributions of this research, we have revised the title of our manuscript to: "**An efficient mid-infrared computational spectrometer based on synergistic microcavity-coupled photonic crystal waveguides**".

Nevertheless, we wish to clarify that the field of on-chip MIR spectroscopy is still in its nascent stages, with a limited number of published works specifically targeting this regime—most of which rely on traditional passive architectures. To provide a more robust evaluation, we have extended our benchmarking to include state-of-the-art computational spectrometers from the NIR domain. This cross-spectral comparison offers a more comprehensive view of our device's performance within the broader landscape of integrated photonics. We have summarized the quantitative metrics (footprint, working band, resolution, bandwidth, channels, wavelength-normalized power, resolution-footprint product, and bandwidth-footprint ratio) in Supplementary Note 16.

Structure	Footprint (μm^2)	WB (µm)	RES (nm)	BW (nm)	CH	Active?	WNP (mW)	RFP ($nm \cdot \mu m^2$)	BFR ($nm \cdot \mu m^{-2}$)
**Random disorder scattering[1]**	1250	NIR (1.51)	0.75	25	25	N	/	5625	0.02
**Stratified waveguide filters[2]**	9100	NIR (1.55)	0.45	180	32	N	/	4095	0.0198
**Cascaded nanobeam[3]**	4500	NIR (1.55)	0.16	16	3	Y	30	720	3.6×10^{-3}

**Grating-assisted F-P cavity**[4]	3.6×10^{4}	NIR (1.50)	0.43	73.2	5	Y	121	1.84×10^{4}	2.9×10^{-3}
**Photonic molecule**[5]	3600	NIR (1.55)	0.04	100	1	Y	75	144	0.028
**Programmable photonic circuits**[6]	7.03×10^{6}	NIR (1.55)	0.01	200	1	Y	350	7.03×10^{4}	1.42×10^{-5}
**Digital FTS**[7]	1.78×10^{6}	NIR (1.55)	0.2	20	1	Y	99	3.55×10^{5}	1.13×10^{-5}
**Ring-assisted FTS**[8]	NA	NIR (1.60)	0.47	90	1	Y	1835	/	/
**High-Q Ring**[9]	3.5×10^{5}	NIR (1.55)	0.005	10	10	Y	50.4	1.75×10^{3}	2.86×10^{-5}
**Micro-disk**[10]	1.25×10^{5}	NIR (1.55)	0.2	20	1	Y	160	2.51×10^{4}	1.59×10^{-4}
**Arrayed Waveguide Grating and Ring**[11]	9×10^{6}	NIR (1.55)	0.1	25.4	9	Y	35	9×10^{5}	2.82×10^{-6}
**FTS Array**[12]	NA	MIR (3.30)	12	60	12	N	/	/	/
**FTS Array**[13]	9.5×10^{7}	MIR (3.75)	2.7	51	42	N	/	2.57×10^{8}	5.37×10^{-7}
**Waveguide Coupler**[14]	2.7×10^{6}	MIR (3.85)	3	350	1	Y	NA	8.1×10^{6}	1.29×10^{-4}
**MPCW (This work)**	**1680**	**MIR (2.00)**	**0.5**	**100**	**8**	**Y**	**45.6**	**840**	**0.06**

WB: waveband, RES: resolution, BW: bandwidth, CH: channel, WNP: wavelength-normalized power, RFP: resolution-footprint product, BFR: bandwidth-footprint ratio.

Changes made to the manuscript:

Supplementary Note 16

16. Comparison of On-chip Spectrometers

Structure	Footprint (μm^2)	WB (μm)	RES (nm)	BW (nm)	CH	Active?	WNP (mW)	RFP (nm·μm^2)	BFR (nm·μm^{-2})
**Random disorder scattering**[1]	1250	NIR (1.51)	0.75	25	25	N	/	5625	0.02
**Stratified waveguide filters**[2]	9100	NIR (1.55)	0.45	180	32	N	/	4095	0.0198
**Cascaded nanobeam**[3]	4500	NIR (1.55)	0.16	16	3	Y	30	720	3.6×10^{-3}
**Grating-assisted F-P cavity**[4]	3.6×10^{4}	NIR (1.50)	0.43	73.2	5	Y	121	1.84×10^{4}	2.9×10^{-3}
**Photonic molecule**[5]	3600	NIR (1.55)	0.04	100	1	Y	75	144	0.028

**Programmable photonic circuits**[6]	7.03×10^{6}	NIR (1.55)	0.01	200	1	Y	350	7.03×10^{4}	1.42×10^{-5}
**Digital FTS**[7]	1.78×10^{6}	NIR (1.55)	0.2	20	1	Y	99	3.55×10^{5}	1.13×10^{-5}
**Ring-assisted FTS**[8]	NA	NIR (1.60)	0.47	90	1	Y	1835	/	/
**High-Q Ring**[9]	3.5×10^{5}	NIR (1.55)	0.005	10	10	Y	50.4	1.75×10^{3}	2.86×10^{-5}
**Micro-disk**[10]	1.25×10^{5}	NIR (1.55)	0.2	20	1	Y	160	2.51×10^{4}	1.59×10^{-4}
**Arrayed Waveguide Grating and Ring**[11]	9×10^{6}	NIR (1.55)	0.1	25.4	9	Y	35	9×10^{5}	2.82×10^{-6}
**FTS Array**[12]	NA	MIR (3.30)	12	60	12	N	/	/	/
**FTS Array**[13]	9.5×10^{7}	MIR (3.75)	2.7	51	42	N	/	2.57×10^{8}	5.37×10^{-7}
**Waveguide Coupler**[14]	2.7×10^{6}	MIR (3.85)	3	350	1	Y	NA	8.1×10^{6}	1.29×10^{-4}
**MPCW (This work)**	**1680**	**MIR (2.00)**	**0.5**	**100**	**8**	**Y**	**45.6**	**840**	**0.06**

WB: waveband, RES: resolution, BW: bandwidth, CH: channel, WNP: wavelength-normalized power, RFP: resolution-footprint product, BFR: bandwidth-footprint ratio.

The wavelength-normalized power (WNP) is defined as:

$$\mathrm{WNP} = P_{\mathrm{working}} \times \frac{P_{\pi}(1550)}{P_{\pi}(\text{center wavelength})}$$

Q3: Sensitivity and optical throughputs: typically, coupling into integrated photonic devices, then in-plane losses (splitters, etc result in very low throughputs and thus combined with fabrication errors etc. I would suspect the sensitivity of this device is low. Can you provide a quantitative measure please? The impact on the performance or detection limits is not discussed.

Reply: The authors appreciate the reviewer's comments. The issue raised by the reviewer is critical. According to your request, we tested the detection limit of our spectrometer. With a dark noise of -62 dBm, we injected a single-wavelength laser with different attenuation values into our spectrometer using a manual attenuator and reconstructed the laser spectrum based on different incident powers. The results are shown in Fig. **R8**. The reconstruction fidelity remains high at sufficient incident power levels. However, when the incident optical power is below 1 μW, the reconstruction fidelity deteriorates. Therefore, the detection limit of incident optical power in our system is approximately 1 μW. On-chip multi-channel splitting does reduce the optical power throughput, but this issue can be mitigated by designing on-chip optical switches. Specifically, utilizing thermo-optic switches for optical routing, rather than static power splitting, allows the signal to be directed to a single channel at a time, thereby maintaining a significantly higher optical power budget.

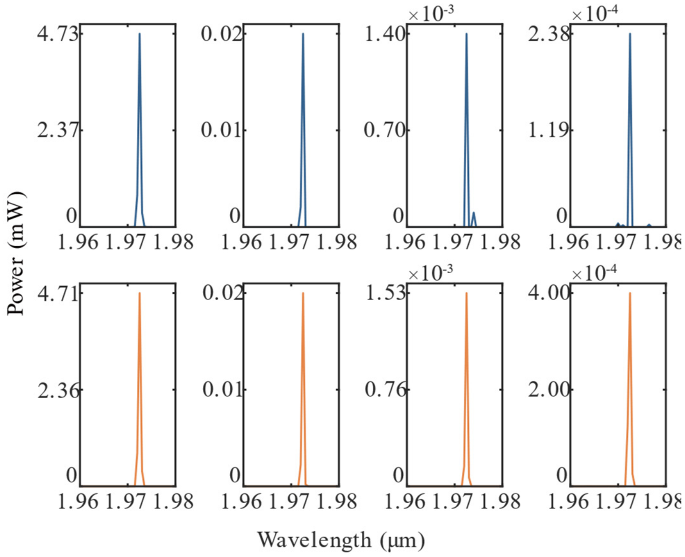

Fig. R8 | Reconstructed spectra under different incident optical powers of lasers. Blue is the reconstruction result and orange is the ground truth.

Further, any slow-light device (or PCW device) is highly susceptible to fabrication tolerances (ie. Resonant linewidth / band edge deviations) and even small deviations degrade performance significantly. Do you have simulation / otherwise results to indicate the tolerances and how you could get around them? i.e. computationally / otherwise.

Reply: The authors appreciate the reviewer's comments. Lithography and fabrication of photonic crystals can typically ensure stable lattice constants, so process deviations are mainly caused by variations in hole diameter. Here, we simulate the variation in spectral response when there is a process error of ±10 nm in hole diameter (Fig. **R9**). One potential solution is to design an approximately additional two MPCW units during manufacturing and compensate for process variations by adjusting the lattice constant. Process deviations within the operating range will be recorded in the calibration matrix during spectrometer calibration. Therefore, the critical requirement is to ensure that the longest wavelength at the band edge can reach the maximum measurement range.

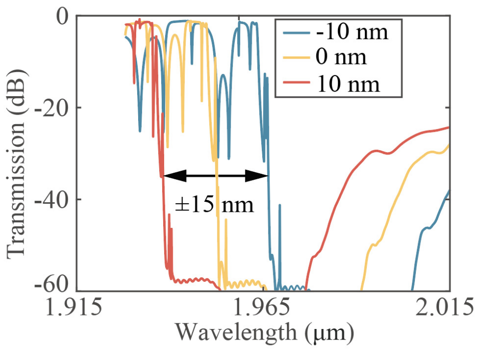

Fig. R9 | Simulation results of response spectrum variation caused by fabrication errors in photonic crystal hole diameters.

Changes made to the manuscript:

Supplementary Note 12

12. Detection Limit

A manual attenuator was utilized to regulate the output power of the laser. The output spectrum of the laser is shown in Fig. **S19**. The detection dark noise of our system is approximately −62 dBm. Notably, the reconstructed optical power exhibits significant deviations when the input power falls below 1 μW. To lower the detection threshold, the beam splitter in the MPCW spectrometer could be substituted with a thermo-optic switch, which can significantly improve the optical power budget.

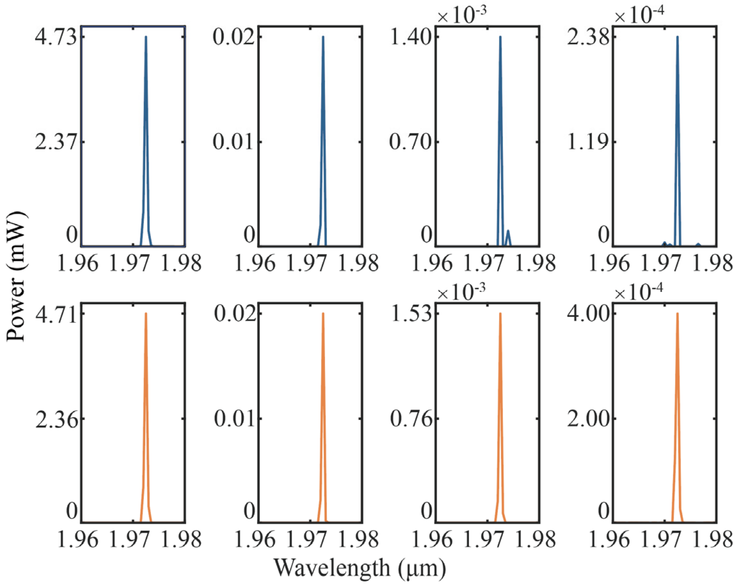

Fig. **S19**. Reconstruction results under different optical powers. The orange curve denotes the reference spectrum, and the blue curve represents the reconstruction result.

Supplementary Note 5

5. Fabrication Tolerance

During fabrication, the positioning error of electron-beam lithography is minimal, but the size of the fabricated holes is difficult to control precisely. Therefore, we simulated the influence of diameter deviations on the transmission characteristics of the filter. The simulation results are shown in Fig. **S8**. The results indicate that when the hole diameter error is within ±10 nm, the band-edge wavelength of the filter exhibits a red-shift or blue-shift of approximately 15 nm due to the variation in hole size.

In practical mass production, photolithography is typically used for the exposure process, and the hole size can be kept relatively stable across different batches. In addition, the impact of fabrication errors on the spectrometer can be reduced by adding two redundant MPCW units to compensate for spectral shifts due to response shifts caused by process tolerances.

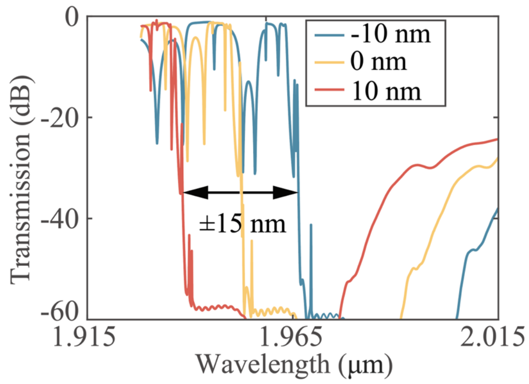

Fig. **S8**. Simulation results of response spectrum variation caused by fabrication errors in photonic crystal hole diameters.

Q4: Cavity design choice: could the authors please discuss why the cavity design was chosen? Was this informed from simulations not shown in the paper? For example, one could imagine using non-uniform cavity designs which may offer 'better' performance.

Reply: The authors appreciate the reviewer's comments. The reviewer has indeed pointed out a significant optimization direction. Admittedly, the non-uniform cavity design by the reviewer may indeed offer superior performance, particularly in enhancing spectral diversity when scaling the system to a larger number of sampling channels. This forward-looking suggestion regarding the scalability of our MPCW platform is invaluable, as it provides a critical long-term optimization path for our technology. Regarding the current implementation, the decision to utilize uniform microcavities was primarily dictated by the limited number of physical channels (eight) in this proof-of-concept study. Therefore, we focused on obtaining new wavelength information via band-edge engineering rather than introducing excessive complexity to the microcavity. However, we believe that introducing non-uniformities into the microcavities would be more efficacious and necessary in future iterations with significantly higher channel densities.

Q5: Thermal tuning and crosstalk: there must be some coupling between channels, especially if this is operated repeatedly. Does this result in performance degradation with time or longer-term thermal drift?

Reply: The authors appreciate the reviewer's comments. In our experiments, the spectrometer operates at room temperature, and we set the operating temperature to 23 °C. To evaluate the influence of temperature on the spectrometer, we measured the variation in the transmission characteristics of the filter over the temperature range from 20 °C to 26 °C (with temperature values: 20, 21, 22.5, 22.9, 23, 23.1, 23.5, 24, 25, 26 °C). The measurement results are shown in Figs. **R10a** and **R10b**. It can be observed that the transmission spectrum of the filter exhibits a redshift as the temperature increases. This is attributed to the refractive index of silicon increases with rising temperature, which shifts the band-edge position of the photonic crystal and consequently leads to a wavelength shift in the operating wavelength of the filter.

To quantify the specific effect of temperature fluctuations on the transmission spectrum of the filter, we used the relative variation (v) to characterize the change in its transmission performance, defined specifically as:

$$v(T) = \frac{\|R(T)-R(T_0)\|_2}{\|R(T_0)\|_2}$$

where $R(T)$ denotes the filter response at temperature T, and T_0 is the reference temperature (set to 23 °C in this work). By calculating the v at different temperatures, the influence of temperature on the transmission characteristics of the filter can be observed more clearly. The calculated results are displayed in Fig. **R10c**. It can be seen that the transmission characteristics of the filter change significantly with increasing temperature, especially near the operating wavelength of the filter, where the variation is more pronounced. This indicates that ambient temperature fluctuations in non-laboratory environments will considerably affect the filter performance, and additional TEC integration is required for compensation in practical applications.

Additionally, since the photonic crystal spectrometer contains multiple filter channels that are

spatially close to each other, heating one channel can affect the transmission characteristics of adjacent channels. **We would like to clarify that we performed single-channel heating and measurement at a time in the experiment.** However, thermal crosstalk would arise if multiple detectors were used and different channels were modulated simultaneously. To evaluate the effect of thermal crosstalk, we heated a single channel and measured the transmission characteristics of its neighboring photonic crystal filters. According to our experimental results, when one channel is heated, the transmission spectra of adjacent channels also change compared with the unheated condition, with an average relative spectral deviation of 11.1%. We attribute this to the absence of additional etched air isolation regions for photonic crystal during fabrication. We present the simulation results in Fig. **R11** for illustration. As shown in the figure, without isolation regions, heat conducts directly through the silicon slab to adjacent channels, raising their temperature by 1.72 K and thus affecting their transmission characteristics, in good agreement with the experimental observations. When isolation regions are added, heat conduction is effectively suppressed, and the temperature variation of neighboring channels is significantly reduced to approximately 0.04 K, thereby greatly mitigating thermal crosstalk. According to Fig. **R12**, a temperature perturbation of 0.04 K corresponds to a relative crosstalk of 0.4%, which is within the acceptable tolerance for practical applications. Therefore, in applications involving multiple detection channels, to reduce the impact of thermal crosstalk on spectrometer performance, it is recommended to incorporate isolation regions in the design and fabrication process to improve device stability and reliability.

To evaluate performance degradation, we measured the temperature fluctuations of the MPCW unit under specific thermal powers (without heating and at maximum heating power), as well as the relative fluctuations of the calibration matrix after multiple heating cycles (Fig. **R13**). The mean relative fluctuations of the two experiments are 0.9% and 0.7%, respectively, indicating that our MPCW unit exhibits excellent repeatability.

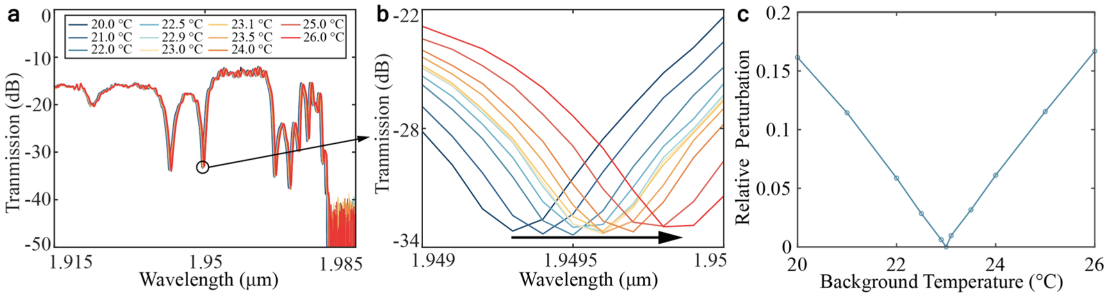

Fig. R10 | a The response spectrum variation of different background temperatures. **b** The enlargement spectrum variation in **a**. **c** The relative perturbation of the response spectrum induced by temperature variation.

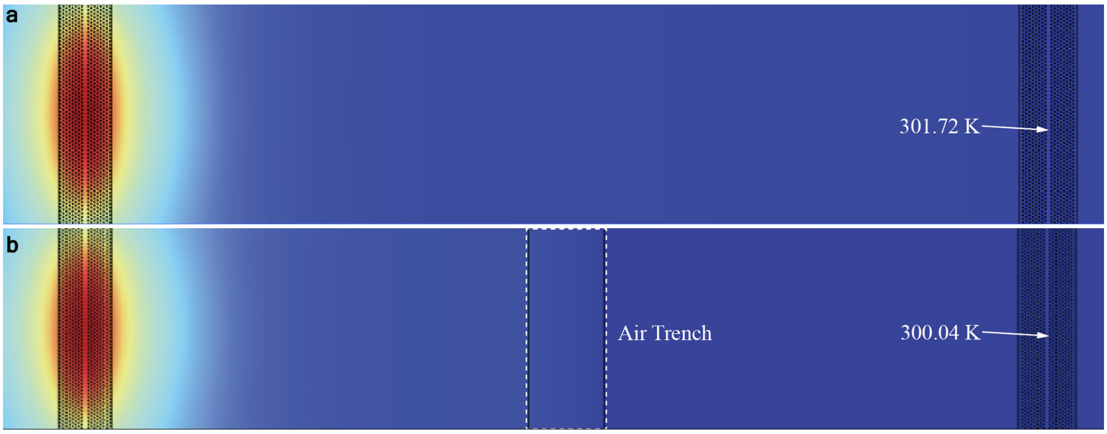

Fig. R11 | Simulation results of heating perturbation **a** with and **b** without air trenches**.**

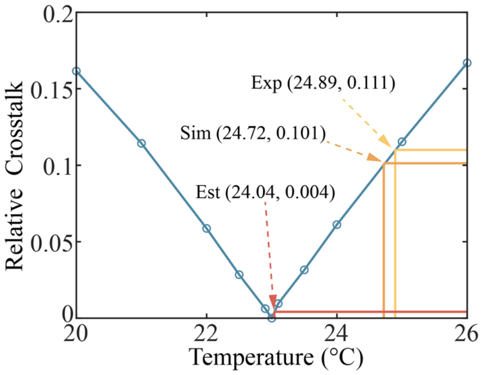

Fig. R12 | Estimation of temperature perturbation after using air trenches based on simulation and experimental results.

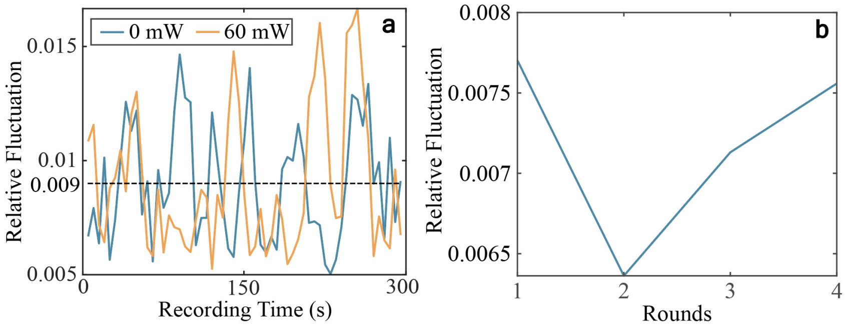

Fig. R13 | a Fluctuations in the response spectra during long-term measurements at a specific heating power. **b** Fluctuations in the calibration matrix across multiple measurement cycles.

Changes made to the manuscript:

Supplementary Note 8

8. Temperature Perturbation

In our experiments, the spectrometer operates at room temperature, and we set the operating temperature to 23 °C. To evaluate the influence of temperature on the spectrometer, we measured the variation in the transmission characteristics of the filter over the temperature range from 20 °C to 26 °C (20.0, 21.0, 22.5, 22.9, 23.0, 23.1, 23.5, 24.0, 25.0, and 26.0 °C). The measurement results are shown in Fig. **S14a** and Fig. **S14b**. The transmission spectrum of the filter exhibits a red shift as the temperature increases. This phenomenon is attributed to the refractive index of silicon increases with rising temperature, which shifts the band-edge position of the photonic crystal and consequently leads to the spectral drift of the filter.

To quantify the specific impact of temperature perturbation on the transmission spectrum of the filter, we used the relative variation $v(T)$ to characterize the change in its transmission behavior, defined explicitly as:

$$v(T) = \frac{\|R(T) - R(T_0)\|_2}{\|R(T_0)\|_2}$$

where $R(T)$ denotes the filter response spectrum at temperature T, and T_0 is the reference temperature (fixed at 23 °C here). Calculating the relative variation at different temperatures allows a clearer observation of the temperature effect on the filter transmission. The calculated results are displayed in Fig. **S14c**. It can be seen that the transmission characteristics of the filter change noticeably with increasing temperature, especially near the operating wavelength of the filter, where the variation is more significant. This indicates that ambient temperature fluctuations in non-laboratory environments can considerably affect the filter performance and highlights the necessity of integrating a TEC for precise thermal stabilization in practical applications.

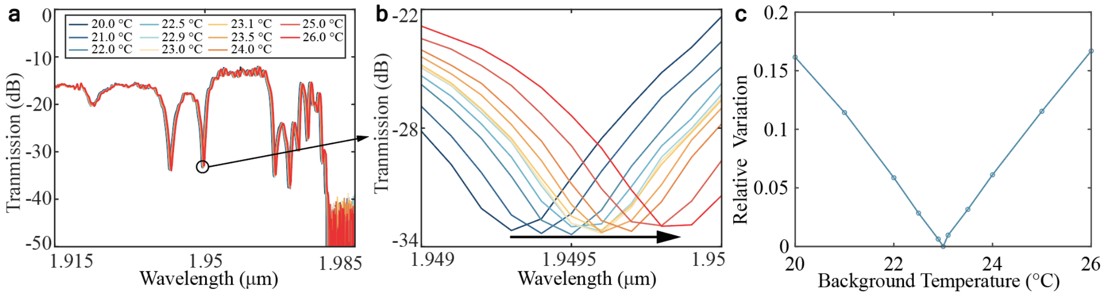

Fig. **S14**. **a** Response of the 6th MPCW channel as a function of temperature. **b** Magnified view of the transmission response **c** Relative spectral variation with reference to 23 °C.

Supplementary Note 9

9. Thermal Crosstalk

As the MPCW spectrometer contains multiple filter channels closely spaced, applying thermal power to a channel can affect the transmission characteristics of adjacent channels. Although our design is intended for single-channel operation, the measurements were conducted by sequentially heating and reading individual channels. The simultaneous excitation of multiple channels with multiple detectors would introduce additional thermal crosstalk.

To evaluate the effect of thermal crosstalk, we heated one channel and measured the transmission characteristics of its neighboring MPCW channels. The experimental results show that heating one channel alters the transmission spectrum of adjacent channels, with relative spectral deviation of 11.1% compared with the unheated condition. Such thermal perturbation can be mitigated by introducing air isolation region (air trench), as illustrated by our simulation results (Fig. **S15**). Without an isolation region, heat conducts directly through the silicon slab to neighboring channels, raising their temperature by approximately 1.72 K and perturbing their response spectra (10.1%), in good agreement with our experimental observations (Fig. **S15a**). With air isolation regions, heat conduction is effectively suppressed, reducing the temperature variation of adjacent channels to only 0.04 K (Fig. **S15b**), corresponding to a relative crosstalk of 0.4% (Fig. **S16**). This significantly alleviates thermal crosstalk.

Therefore, in multi-channel detection applications, deeply etched air isolation trenches can be adopted during the design and fabrication process to suppress thermal crosstalk and improve the

stability and reliability of multi-channel spectral measurements.

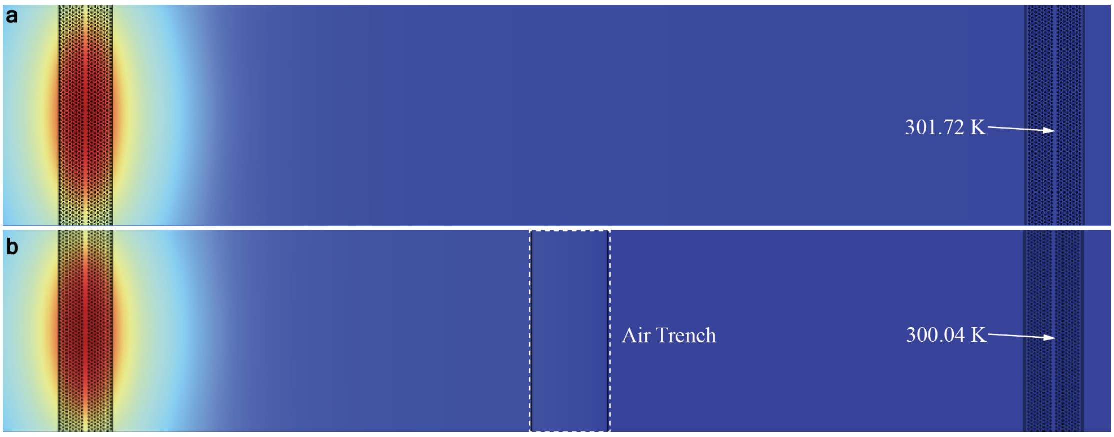

Fig. **S15**. Simulation results of heating perturbation **a** with and **b** without air trenches.

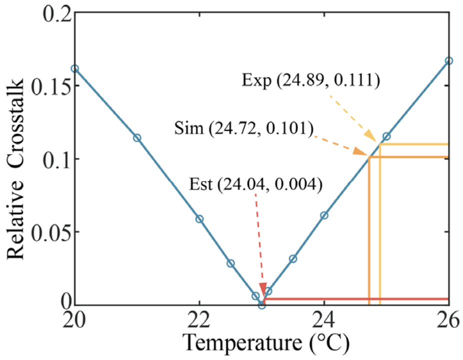

Fig. **S16**. Estimation of temperature perturbation after using air trenches based on simulation and experimental results.

Supplementary Note 10

10. Operation Stability

For the operational repeatability, we first measured the average relative fluctuation of the spectra of the MPCW element at 0 mW and 60 mW (with the TEC base temperature set to 23 °C). Similar to temperature-induced variations, the average spectrum fluctuation is defined as:

$$f(t) = \frac{\left\| R(t) - R_{average} \right\|_2}{\left\| R_{average} \right\|_2}$$

where $R(t)$ is the response spectrum acquired at time t, and $R_{average}$ is the average response spectrum taken at different times. As shown in Fig. **S17a**, the average relative fluctuation of the spectrum is approximately 0.9%, with the maximum fluctuation not exceeding 1.6%. These fluctuations are primarily attributed to the precision limit of TEC controller.

Furthermore, we performed four complete measurements of the response matrix at 30-minute intervals. In each measurement cycle, the filters underwent a full heating and cooling cycle. We calculated the relative variation of the average transmission characteristics for each channel across different measurement rounds. As shown in Fig. **S17b**, the average relative fluctuation of the entire response matrix is roughly 0.7%. This demonstrates that the designed thermo-optically controlled photonic crystal filters maintain high operational repeatability across multiple thermal tuning cycles.

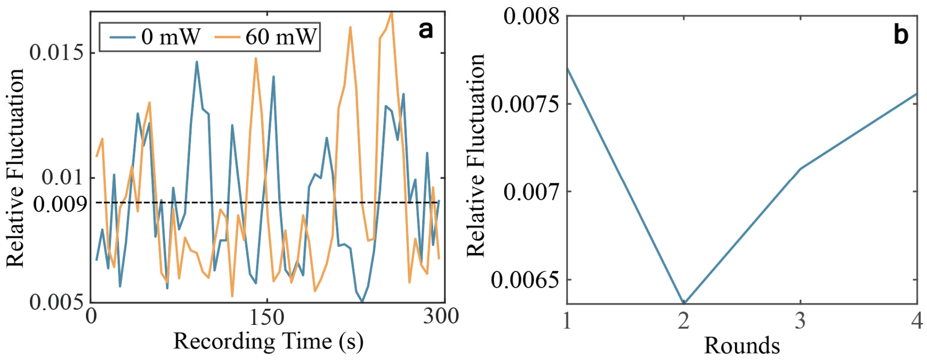

Fig. **S17**. **a** Fluctuations in the response spectra during long-term measurements at a specific heating power. **b** Fluctuations in the calibration matrix across multiple measurement cycles.

Q6: Clarification of "… due to the limitations of hardware sampling and computational resources, obtaining a continuous spectrum is impractical."

Reply: The authors appreciate the reviewer's comments. We apologize for the ambiguity in our original statement, which has caused confusion. We would like to clarify that our core intention is to illustrate the discretization of the spectral integral equation in practical computations—i.e., converting the theoretical integral form (Eq. (1)):

$$\mathrm{d}_{(a_i,P_j)} = \int_{\lambda_\mathrm{I}}^{\lambda_\mathrm{F}} \mathrm{T}_{(a_i,P_j)}(\lambda)\mathrm{s}(\lambda)\mathrm{d}\lambda,$$

into a discrete matrix form (Eq. (2)):

$$\mathbf{d} = \mathbf{Ts},$$

to facilitate numerical implementation, rather than focusing solely on hardware or resource constraints. To avoid ambiguity, we have rewritten this sentence.

Changes made to the manuscript:

The sampling process of our MPCW units is governed by the following integral equation:

$$\mathrm{d}_{(a_i,P_j)} = \int_{\lambda_\mathrm{I}}^{\lambda_\mathrm{F}} \mathrm{T}_{(a_i,P_j)}(\lambda)\mathrm{s}(\lambda)\mathrm{d}\lambda, \quad (1)$$

where λ_I and λ_F indicate the initial and final wavelengths of the spectral range to be reconstructed. $\mathrm{T}_{(a_i,P_j)}(\lambda)$ defines the power transmission function of the sampling step (a_i, P_j), and $\mathrm{s}(\lambda)$ denotes the input spectrum. The output power is represented by $\mathrm{d}_{(a_i,P_j)}$. For spectral integration problems, the continuous integral equation must be discretized for numerical solution. Specifically, by aggregating the responses of all channels, the transmission matrix $\mathbf{T}$ (or calibration matrix, as depicted in Fig. 1c) is constructed, and this matrix is then used to approximate Eq. (1) in matrix form:

$$\mathbf{d} = \mathbf{Ts} \quad (2)$$

Q7: Computational reconstruction: The combined L1-L2 based reconstruction approach: These approaches are quite susceptible to noise and the actual reconstruction quality (i.e. some types of signals are poorly reconstructed).

Can you please quantify noise metrics and reconstruction quality to ground truth spectra of additional signal types (not just a laser or ASE), something more akin to sensing (molecular analyte)? I'm concerned that the chosen inputs are selected due to the reconstruction 'ease' / architecture being poor at reconstructing say more natural signals or more complex signals. Please alleviate my

concerns. Also, normally one enforces the idea of sparsity or smooth signals, and thus real-world spectra may not 'adhere' to these constraints.

Reply: The authors appreciate the reviewer's comments. We constructed a prototype CO_2 gas cell with a length of approximately 1 m and used two collimated fibers to facilitate the coupling and propagation of 2 μm light within the cell. The reference spectrum was measured using a conventional spectrometer. At a spectrometer resolution of 0.5 nm, fine absorption features remained unresolved, yielding a broader absorption envelope relative to the results obtained at a higher resolution of 0.1 nm. Furthermore, the instability of the CO_2 source led to inconsistent gas flow, which introduced pronounced power fluctuations during the sampling phase. Consequently, the reconstruction error was significantly larger than that of a typical ASE spectrum. Nevertheless, the CO_2 absorption envelope can still be clearly distinguished from the reconstructed spectrum shown in Fig. **R14**.

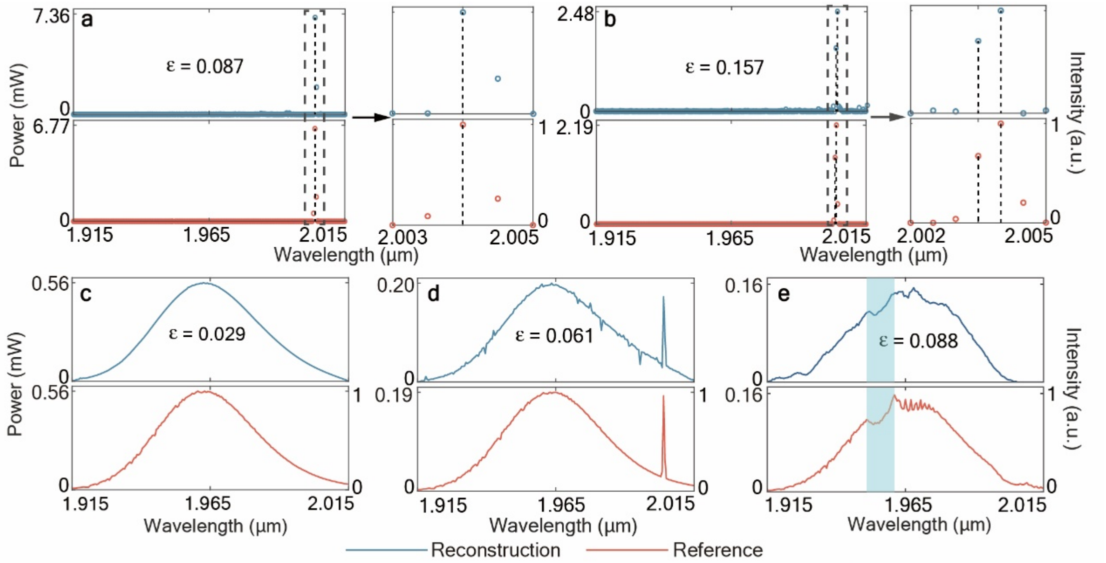

Fig. R14 | a-e The blue line is the algorithm reconstruction result, and the red line is the reference measured by the commercial spectrometer. **a** Single peak input spectrum and its reconstructed result. The peak is at 2004 nm. The right side shows an enlargement of the left side, which exhibits a full width at half maximum (FWHM) of 0.5 nm. **b** Dual peaks reconstructed result. An additional peak is set at 2003.5 nm adjacent to the 2004 nm peak. The right side displays a zoomed-in view of the left side. Compared to the a, the reconstruction result displays the additional peak line. **c** Broadband ASE input spectrum and its reconstructed result. **d** The input hybrid spectrum, which combines the ASE and a laser at 2004 nm. **e** Spectrum of ASE after absorption by carbon dioxide and its reconstruction result. The blue region represents the reconstructed absorption envelope.

Changes made to the manuscript:

In addition, we constructed a prototype 1-m-long carbon dioxide gas cell. Following the alignment of two collimated fibers, the 2 μm ASE light source was coupled into the gas cell to acquire the absorption spectrum. Due to the lack of precise flow control in the carbon dioxide delivery system, the gas concentration fluctuated during the sampling process, leading to a degradation in reconstruction fidelity. The carbon dioxide absorption envelope remains clearly discernible in the reconstructed spectrum in Fig. 5e. Reconstruction noise of the laser source (AdValue Photonics) itself is detailed in Supplementary Note 13. To verify the consistency of the MPCW spectrometer, five independent sampling trials were conducted for each scenario and the mean and standard deviation of the reconstruction errors were computed under different numbers of sampling channels. The results are presented in Supplementary Note 14. These results also confirm the effectiveness of

our reconstruction algorithm, even under moderate noise levels.

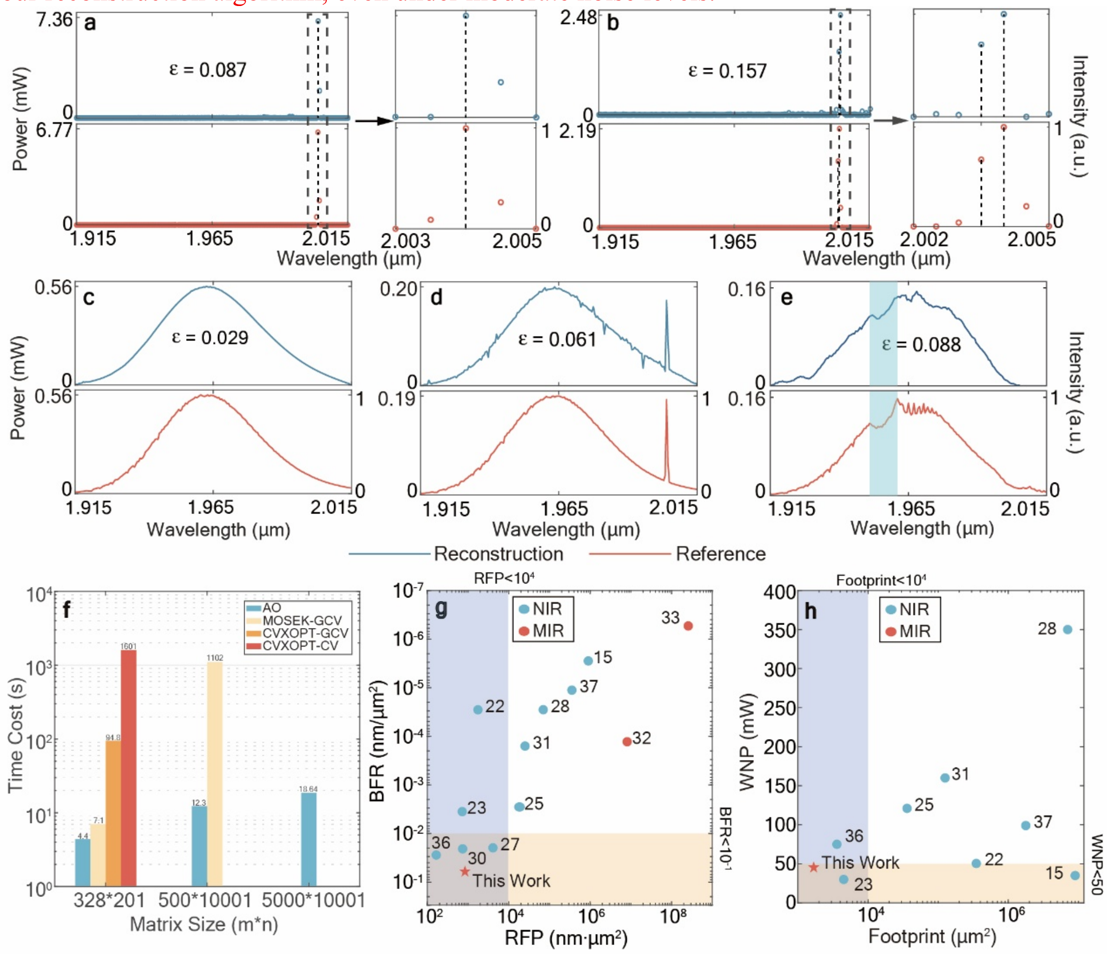

Fig. 5 | Reconstruction results and performance comparison. a-e The blue line is the algorithm reconstruction result, and the red line is the reference measured by the commercial spectrometer. **a** Single peak input spectrum and its reconstructed result. The peak is at 2004 nm. The right side shows an enlargement of the left side, which exhibits a full width at half maximum (FWHM) of 0.5 nm. **b** Dual peaks reconstructed result. An additional peak is set at 2003.5 nm adjacent to the 2004 nm peak. The right side displays a zoomed-in view of the left side. Compared to the **a**, the reconstruction result displays the additional peak line. **c** Broadband ASE input spectrum and its reconstructed result. **d** The input hybrid spectrum, which combines the ASE and a laser at 2004 nm. **e** Spectrum of ASE after absorption by carbon dioxide and its reconstruction result. The blue region represents the reconstructed absorption envelope. **f** Reconstruction efficiency of different methods for different matrix dimensions. For larger-scale problems, the reconstruction time of our alternating optimization does not increase significantly. Direct solution via convex optimization was implemented using CVXPY (solver: CVXOPT and MOSEK), while the alternating optimization method was carried out with the IRtools. Calculations were all performed on a laptop with a 10-core Apple M1 Pro CPU and 16 GB of RAM. AO: alternating optimization; MOSEK-GCV: convex optimization with MOSEK backend with GCV; CVXOPT-GCV: convex optimization with CVXOPT backend with GCV; CVXOPT-CV: convex optimization with CVXOPT backend with 10-fold-CV.The spectrometer performance is compared with the focus on **g** resolution-footprint product (RFP) and bandwidth-footprint ratio (BFR), **h** wavelength-normalized-power (WNP) and footprint.

Q8: Figure 4e (and data). Thermally tuned transmission spectra: The variation in the spectral function as a function of temperature (power) is quite minimal. For computational reconstruction, maximal variation in the encoder functions is normally preferred, therefore why is this PCW structure chosen (ie ordered) and not something more random / quasi-random? How can you increase the variation of the spectra rather than just band edge spectral shift?

Reply: The authors appreciate the reviewer's comments. The reviewer's question is insightful. We have considered using disordered photonic crystal structures; however, the random spectra resulting from such designs require increased number of independent spectral channels. Thermo-optic effects predominantly manifest as spectral shifts. Our focus is on sampling via a combination of band-edge filtering and microcavity assistance, with the primary goal of continuously sampling new wavelength information through band-edge movement.

In addition, more random structures introduce higher scattering loss, and fabrication errors lead to inconsistencies between simulations and experimental measurements, making the transmission spectra difficult to predict and compensate for. Furthermore, we aim to utilize the band-edge movement of photonic crystals to provide relatively detailed spectral information.

Certainly, the approach suggested by the reviewer merits further consideration. We recognize that implementing more randomized or disordered structures while maintaining system stability and performance robustness is a sophisticated process that warrants in-depth investigation. Accordingly, we intend to explore the role of such architecture in integrated spectrometers in our future work.

Q9: Subspeciality to error: How susceptible is your architecture to sources and magnitude of error? There is no mention of matrix condition number or reconstruction error bounds.

Reply: The authors appreciate the reviewer's comments. We resampled each of the five reconstructed spectral cases in the manuscript five times, performed reconstruction using these sampled results, and calculated the mean ε_{mean} and standard deviation ε_{STD} of the relative errors of the reconstructed outcomes (Fig. **R15**). In addition, the conditions number for our calibration matrix is 9793.4.

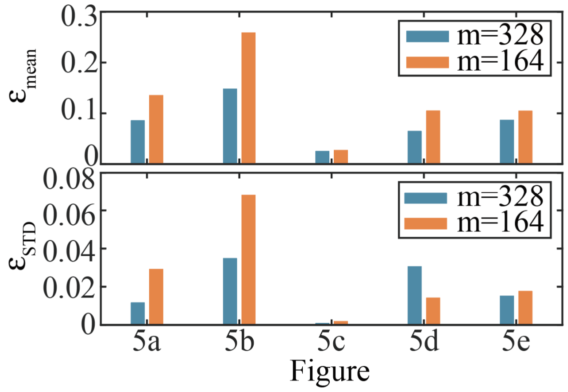

Fig. R15 | The mean ε_{mean} and standard deviation ε_{STD} of reconstruction error based on Fig. 5 presented in the main text**.**

Changes made to the manuscript:

Supplementary Note 14

14. Reconstruction Consistency

To evaluate the reliability of the results, we performed an additional five sampling reconstructions for each case in the main text and calculated the mean ε_{mean} and standard deviation ε_{STD} of the

reconstruction errors across these five independent reconstructions. In addition to our standard sampling (m = 328), we also adopted an under-sampling scheme for spectral reconstruction (m = 164). As illustrated in Fig. **S21**, ε_{mean} of narrow-band spectra (5a, 5b) are more sensitive to the number of channels, and finer sampling results in lower narrow-band reconstruction errors. In contrast, the reconstruction errors of broadband spectra (5c) are less sensitive to the number of sampling channels.

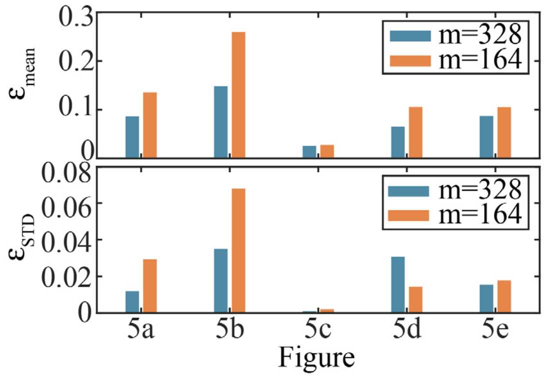

Fig. **S21**. The mean ε_{mean} and standard deviation ε_{STD} of reconstruction error based on Fig. 5 presented in the main text.

Why is 7% random noise chosen? Also, could you please indicate actual power too rather than normalized power (i.e. in Fig 3e)? this can be in the text if needed. Building on this, it is normal to include an idea of minimum noise floor for spectrometer systems (dark noise conventionally), what is this for this system?

Reply: The authors appreciate the reviewer's comments. Given that the output power of our 2-μm laser (AdValue Photonics) is not stable, with a power fluctuation of approximately 7% (Fig. **R16**, Supplementary Notes 13), we used this 7% noise as the noise level in the simulation. Fig. 3e is used to verify the stability of the device design and algorithm in the simulation under 7% random noise level; therefore, we only need to use normalized power as the simulation power. Regarding the minimum noise, according to the content in Q3, the system is relatively stable when the incident optical power is above approximately 1 μW. Concerning the absolute optical power issue, we present the real reconstructed power in addition to the normalized power in the experimental results, which is labeled on the left y-axis of the figure (Fig. **R17**).

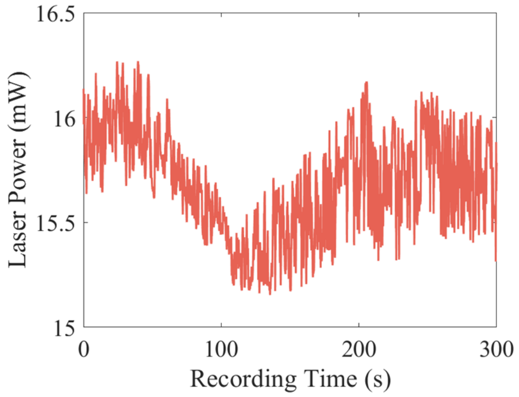

Fig. **R16**. Measured laser output power stability over 5 minutes following a 30-minute warm-up.

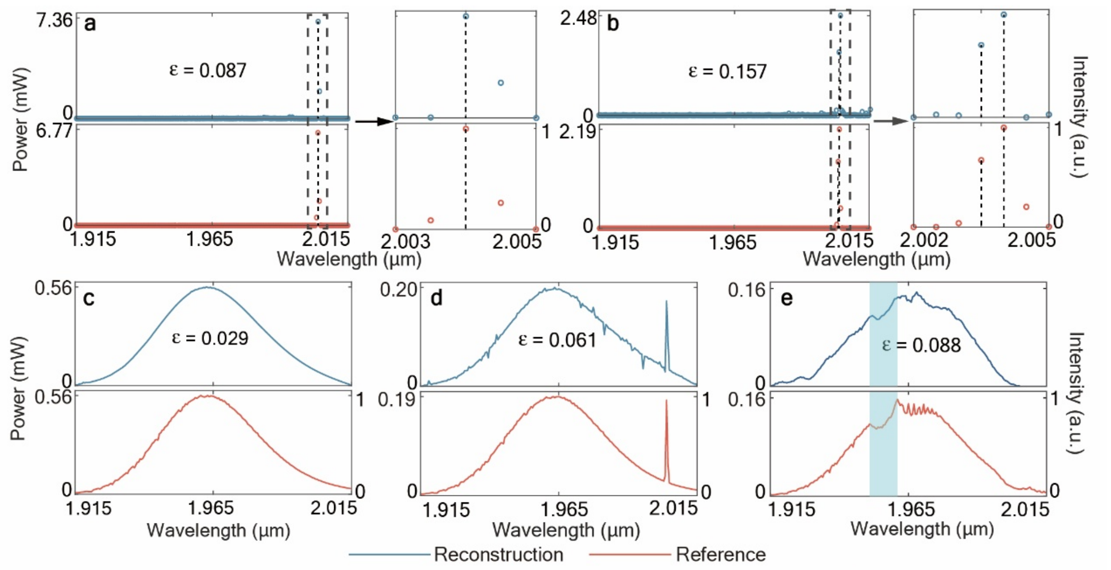

Fig. R17 | a-e The blue line is the algorithm reconstruction result, and the red line is the reference measured by the commercial spectrometer. **a** Single peak input spectrum and its reconstructed result. The peak is at 2004 nm. The right side shows an enlargement of the left side, which exhibits a full width at half maximum (FWHM) of 0.5 nm. **b** Dual peaks reconstructed result. An additional peak is set at 2003.5 nm adjacent to the 2004 nm peak. The right side displays a zoomed-in view of the left side. Compared to the **a**, the reconstruction result displays the additional peak line. **c** Broadband ASE input spectrum and its reconstructed result. **d** The input hybrid spectrum, which combines the ASE and a laser at 2004 nm. **e** Spectrum of ASE after absorption by carbon dioxide and its reconstruction result. The blue region represents the reconstructed absorption envelope.

Changes made to the manuscript:

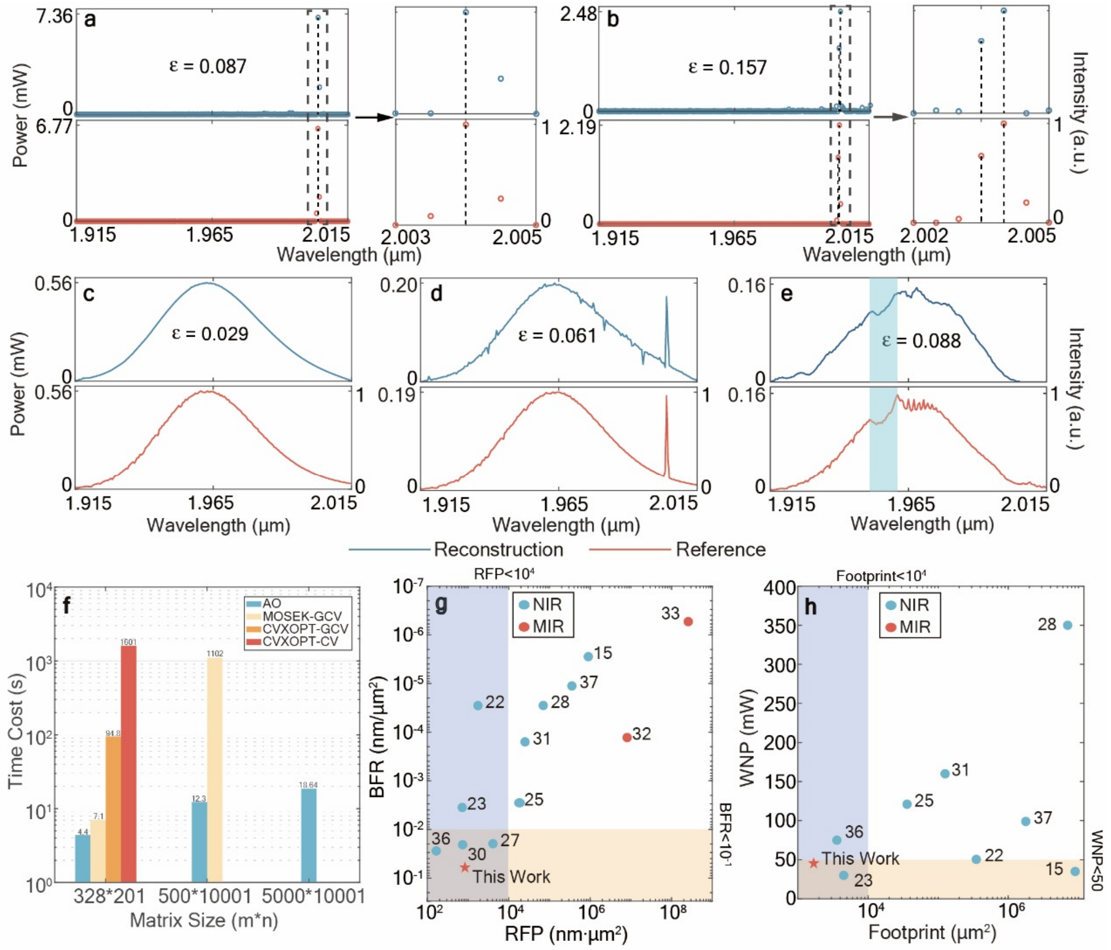

Fig. 5 | Reconstruction results and performance comparison. a-e The blue line is the algorithm reconstruction result, and the red line is the reference measured by the commercial spectrometer. **a** Single peak input spectrum and its reconstructed result. The peak is at 2004 nm. The right side shows an enlargement of the left side, which exhibits a full width at half maximum (FWHM) of 0.5 nm. **b** Dual peaks reconstructed result. An additional peak is set at 2003.5 nm adjacent to the 2004 nm peak. The right side displays a zoomed-in view of the left side. Compared to the **a**, the reconstruction result displays the additional peak line. **c** Broadband ASE input spectrum and its reconstructed result. **d** The input hybrid spectrum, which combines the ASE and a laser at 2004 nm. **e** Spectrum of ASE after absorption by carbon dioxide and its reconstruction result. The blue region represents the reconstructed absorption envelope. **f** Reconstruction efficiency of different methods for different matrix dimensions. For larger-scale problems, the reconstruction time of our alternating optimization does not increase significantly. Direct solution via convex optimization was implemented using CVXPY (solver: CVXOPT and MOSEK), while the alternating optimization method was carried out with the IRtools. Calculations were all performed on a laptop with a 10-core Apple M1 Pro CPU and 16 GB of RAM. AO: alternating optimization; MOSEK-GCV: convex optimization with MOSEK backend with GCV; CVXOPT-GCV: convex optimization with CVXOPT backend with GCV; CVXOPT-CV: convex optimization with CVXOPT backend with 10-fold-CV.The spectrometer performance is compared with the focus on **g** resolution-footprint product (RFP) and bandwidth-footprint ratio (BFR), **h** wavelength-normalized-power (WNP) and footprint.

Q10: Figure 3b and 3c need scale bars. 3b needs identification of what each part of the micrograph is. Fig 4c needs a scale bar and identification of what each part is.

Reply: The authors appreciate the reviewer's comments. Here, we optimized Figs. 3b and 3c (corresponding to Figs. **R18 b** and **c**), and 4c (corresponding to Figs. **R19 c**) in the manuscripts.

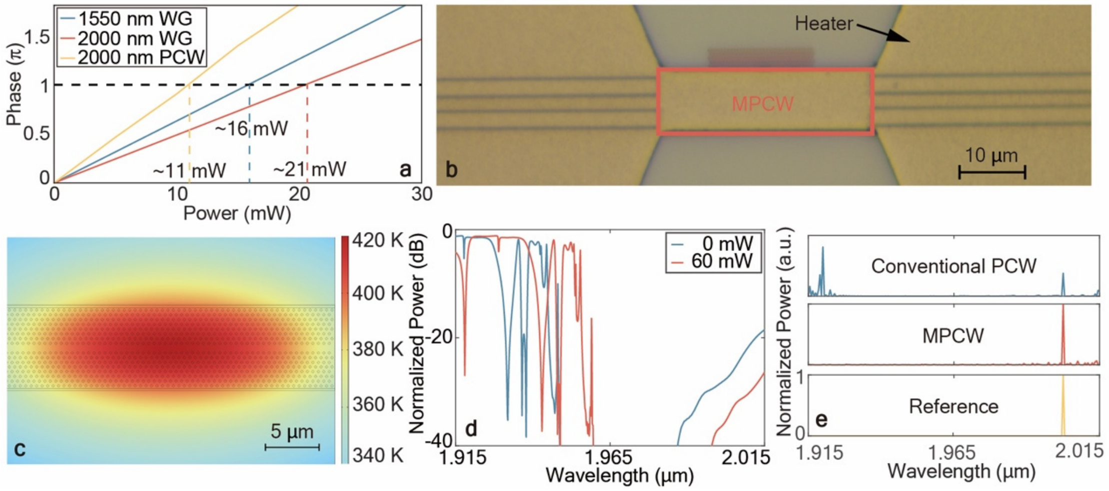

Fig. R18 | Design of the active section of our MPCW. a Comparison of simulated TO tuning efficiency for conventional strip waveguides (1550 nm and 2000 nm) and the PCW operating at the wavelength of 2000 nm. **b** Optical image of the MPCW covered by a Ti heater. **c** Top view of the simulated steady-state thermal distribution in the PC device at 60 mW heating power. **d** Simulated transmission spectra of the MPCW under heating powers of 0 mW and 60 mW. **e** Comparison of reconstruction performance under 7% random detection noise. The yellow line represents the original input spectrum, the blue line corresponds to the spectrum reconstructed using a conventional PCW, and the red line denotes the spectrum reconstructed using the MPCW.

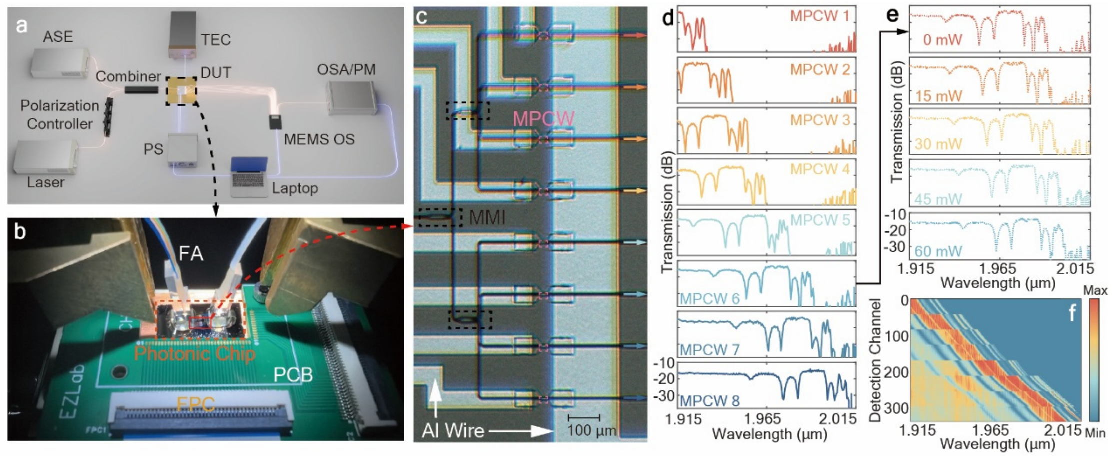

Fig. R19 | Experimental setup, devices characterization and calibration. a Schematic of the experimental setup. Red lines indicate optical paths (fiber), and blue lines indicate electrical control and data acquisition. ASE: amplified spontaneous emission source; PS: power supply; DUT: device under test; TEC: thermoelectric cooler; MEMS OS: micro-electromechanical systems optical switch; OSA: optical spectrum analyzer; PM: power meter. **b** Image of the packaged chip mounted on a PCB. **c** Optical microscope image of the MPCW spectrometer. The pink dashed box denotes the MPCW unit, and the black dashed box represents the MMI. White wires represent Al electrical connections, while black lines correspond to photonic waveguides. The passive measurement results of each channel are depicted in **d,** with corresponding colors. **d** Measured transmission spectra of each MPCW channel, illustrating the shift of band edge and resonance wavelengths with increasing

lattice constant. **e** Thermally tuned transmission spectra of the 6th MPCW channel under heating powers of 0, 15, 30, 45, and 60 mW. In **d** and **e**, the increased spectral noise in the longer-wavelength range is attributed to reduced source power at these wavelengths. **f** Measured transmission matrix **T** of the MPCW spectrometer, constructed through calibration.

Changes made to the manuscript:

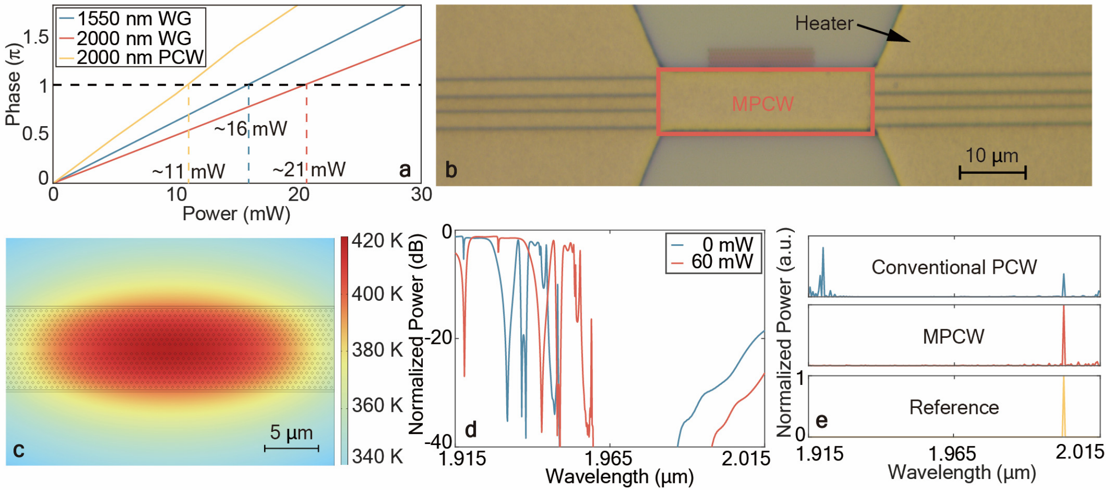

Fig. 3 | Design of the active section of our MPCW. a Comparison of simulated TO tuning efficiency for conventional strip waveguides (1550 nm and 2000 nm) and the PCW operating at the wavelength of 2000 nm. **b** Optical image of the MPCW covered by a Ti heater. **c** Top view of the simulated steady-state thermal distribution in the PC device at 60 mW heating power. **d** Simulated transmission spectra of the MPCW under heating powers of 0 mW and 60 mW. **e** Comparison of reconstruction performance under 7% random detection noise. The yellow line represents the original input spectrum, the blue line corresponds to the spectrum reconstructed using a conventional PCW, and the red line denotes the spectrum reconstructed using the MPCW.

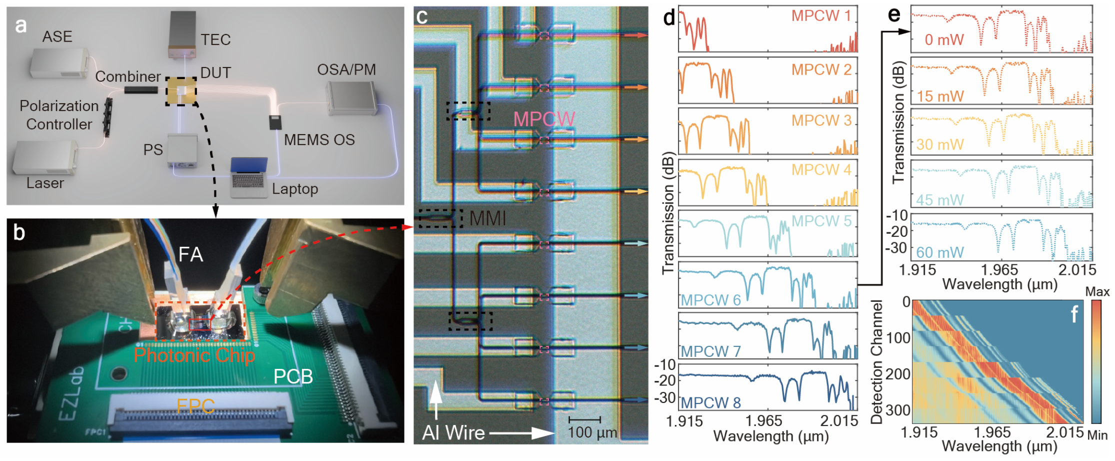

Fig. 4 | Experimental setup, devices characterization and calibration. a Schematic of the experimental setup. Red lines indicate optical paths (fiber), and blue lines indicate electrical control and data acquisition. ASE: amplified spontaneous emission source; PS: power supply; DUT: device under test; TEC: thermoelectric cooler; MEMS OS: micro-electromechanical systems optical switch; OSA: optical spectrum analyzer; PM: power meter. **b** Image of the packaged chip mounted on a PCB. **c** Optical microscope image of the MPCW spectrometer. The pink dashed box denotes the MPCW unit, and the black dashed box represents the MMI. White wires represent Al electrical

connections, while black lines correspond to photonic waveguides. The passive measurement results of each channel are depicted in **d,** with corresponding colors. **d** Measured transmission spectra of each MPCW channel, illustrating the shift of band edge and resonance wavelengths with increasing lattice constant. **e** Thermally tuned transmission spectra of the 6th MPCW channel under heating powers of 0, 15, 30, 45, and 60 mW. In **d** and **e**, the increased spectral noise in the longer-wavelength range is attributed to reduced source power at these wavelengths. **f** Measured transmission matrix **T** of the MPCW spectrometer, constructed through calibration.

Q11: The thermally tuned architecture appears relatively susceptible to ambient (or unwanted local) temperature fluctuations. The authors state the packaged chip is integrated with a TEC, however this is power hungry and bulky, is this a limitation for this approach? For example, even if the PIC is small, the cooler + MEMS optical switch + other elements etc. will always limit the system.

Reply: The authors appreciate the reviewer's comments. In our experiments, the TEC is primarily utilized for thermal stabilization. Since the power consumption of our micro-heater is relatively low and the device operates at room temperature (23 °C as used in our tests), a low-power semiconductor TEC with passive cooling is sufficient to meet the requirements. The demonstrated system is a prototype, and the TEC employed in the experiments is designed for compatibility with general coupling platforms. A TEC similar to those used in standard butterfly-packaged laser modules can achieve lower power demand. In addition, the function of optical switching can be directly implemented on-chip using Mach-Zehnder interferometer (MZI) instead of discrete MEMS optical switches. The electrical power consumption of a single MZI is approximately 21 mW, and the estimated total power for an MZI-based optical switch array is around 130 mW, which remains well within the system's power budget.

Q12: Fig 5 f g, can you please indicate what the shaded blue and yellow regions represent.

Reply: The authors appreciate the reviewer's comments. We have marked specific values at the regional boundaries indicated in Fig. **R20**, aiming to present the results more clearly.

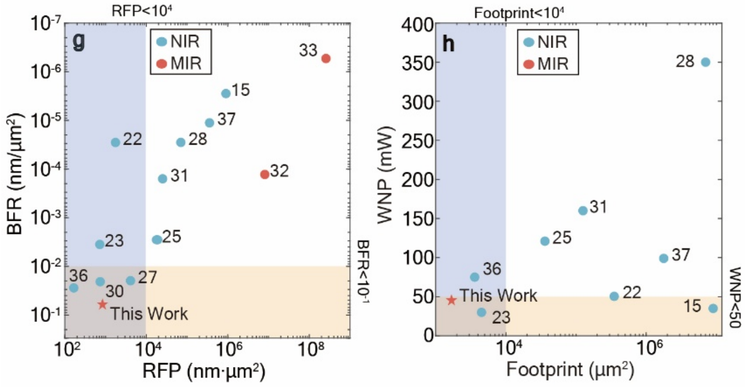

Fig. R20 | g resolution-footprint product (RFP) and bandwidth-footprint ratio (BFR), **h** wavelength-normalized-power (WNP) and footprint.

Changes made to the manuscript:

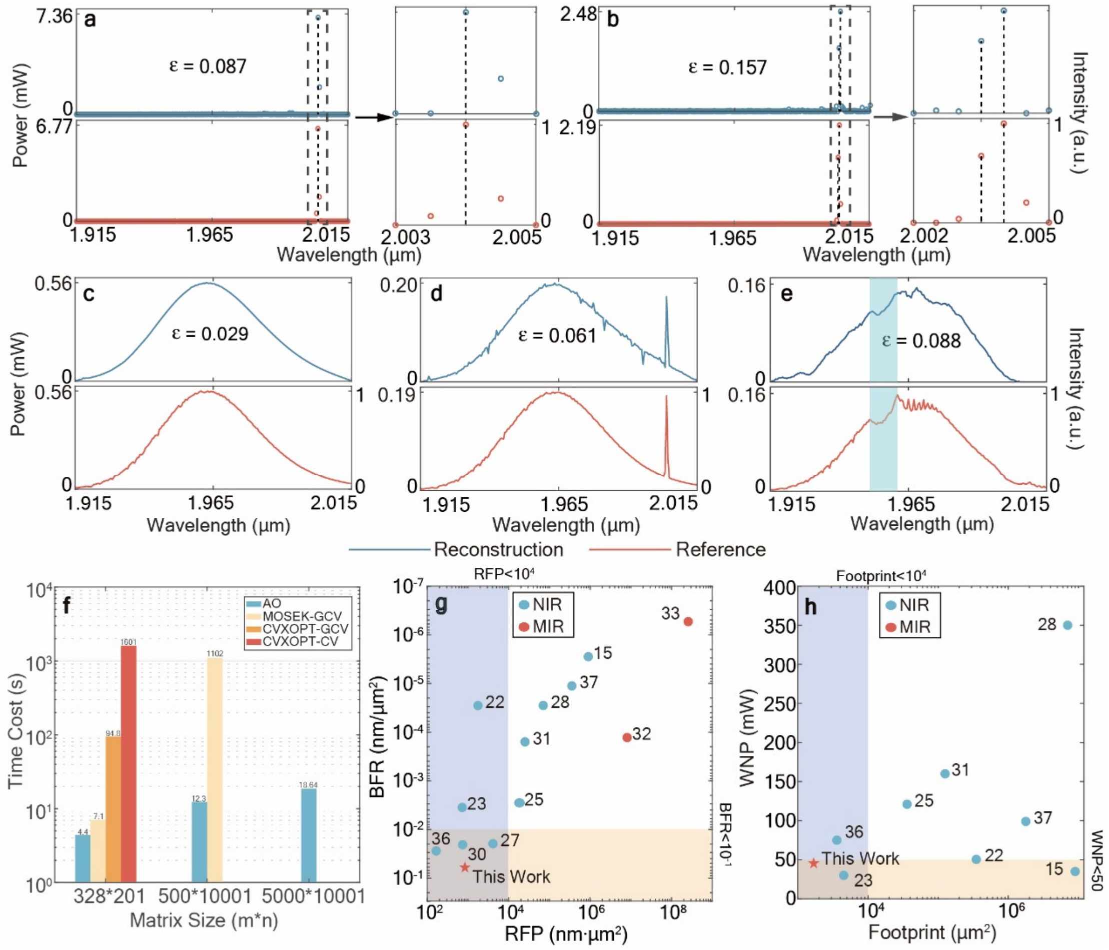

Fig. 5 | Reconstruction results and performance comparison. a-e The blue line is the algorithm reconstruction result, and the red line is the reference measured by the commercial spectrometer. **a** Single peak input spectrum and its reconstructed result. The peak is at 2004 nm. The right side shows an enlargement of the left side, which exhibits a full width at half maximum (FWHM) of 0.5 nm. **b** Dual peaks reconstructed result. An additional peak is set at 2003.5 nm adjacent to the 2004 nm peak. The right side displays a zoomed-in view of the left side. Compared to the **a**, the reconstruction result displays the additional peak line. **c** Broadband ASE input spectrum and its reconstructed result. **d** The input hybrid spectrum, which combines the ASE and a laser at 2004 nm. **e** Spectrum of ASE after absorption by carbon dioxide and its reconstruction result. The blue region represents the reconstructed absorption envelope. **f** Reconstruction efficiency of different methods for different matrix dimensions. For larger-scale problems, the reconstruction time of our alternating optimization does not increase significantly. Direct solution via convex optimization was implemented using CVXPY (solver: CVXOPT and MOSEK), while the alternating optimization method was carried out with the IRtools. Calculations were all performed on a laptop with a 10-core Apple M1 Pro CPU and 16 GB of RAM. AO: alternating optimization; MOSEK-GCV: convex optimization with MOSEK backend with GCV; CVXOPT-GCV: convex optimization with CVXOPT backend with GCV; CVXOPT-CV: convex optimization with CVXOPT backend with 10-fold-CV.The spectrometer performance is compared with the focus on **g** resolution-footprint product (RFP) and bandwidth-footprint ratio (BFR), **h** wavelength-normalized-power (WNP) and footprint.

Q13: Throughout the paper (especially the figures), normalized power / transmission is used. It’s

unclear as to what these are normalized against (e.g. themselves, or some other source, etc. etc.). When comparing to other approaches (which are normally higher optical throughput / efficiency) it would be beneficial to know the actual numerical values.

Reply: We sincerely appreciate your valuable comments, which have helped us further improve the data presentation of our manuscript. To facilitate the intuitive comparison of the spectral profiles across different spectra, all normalized power and transmission spectra in the manuscript have been normalized to their respective peaks. We agree that providing only normalized data can limit the interpretability of the results. To address this issue, we have revised all the figures involving normalized spectra in the manuscript: an additional axis of light intensity (unit: mW) has been added on the left side of each figure (Fig. **R21**), with the actual light intensity values of the corresponding spectra marked synchronously. This allows readers to obtain both the morphological characteristics and absolute power information of the spectra at the same time, improving the comparative value of the data.

We would like to thank you again for your suggestions. This revision has significantly enhanced the rigor and readability of the results presented in the paper.

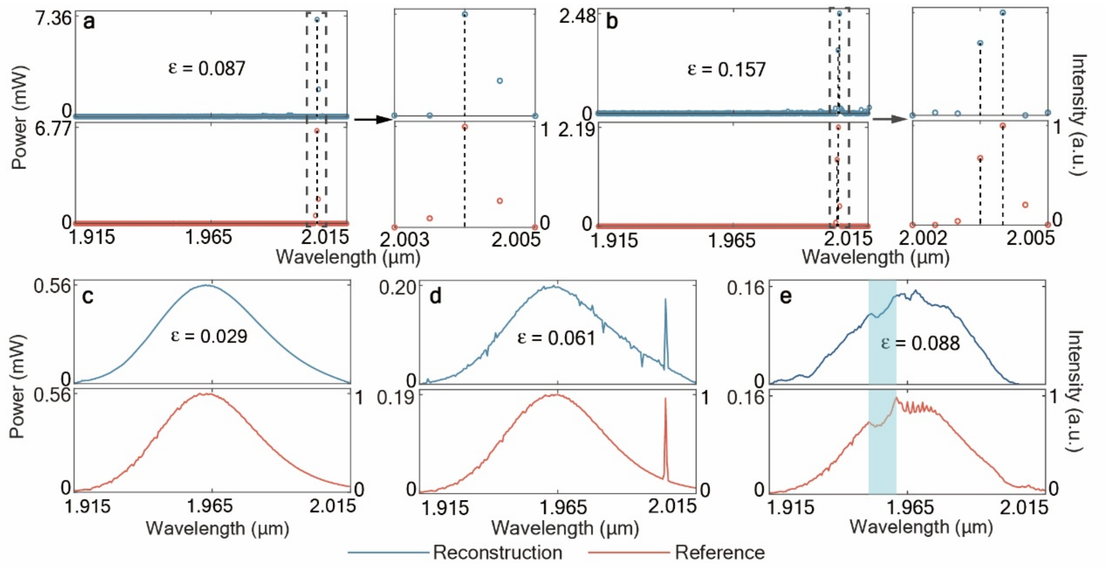

Fig. R21 | a-e The blue line is the algorithm reconstruction result, and the red line is the reference measured by the commercial spectrometer. **a** Single peak input spectrum and its reconstructed result. The peak is at 2004 nm. The right side shows an enlargement of the left side, which exhibits a full width at half maximum (FWHM) of 0.5 nm. **b** Dual peaks reconstructed result. An additional peak is set at 2003.5 nm adjacent to the 2004 nm peak. The right side displays a zoomed-in view of the left side. Compared to the a, the reconstruction result displays the additional peak line. **c** Broadband ASE input spectrum and its reconstructed result. **d** The input hybrid spectrum, which combines the ASE and a laser at 2004 nm. **e** Spectrum of ASE after absorption by carbon dioxide and its reconstruction result. The blue region represents the reconstructed absorption envelope.

Further, what would be useful is to know whether the 4 seconds of reconstruction is down to the need to 'collect more light' or the reconstruction algorithm. Thus, could you include a plot of potentially reconstruction accuracy as a function of time? As for example, there are some applications which require highly accurate spectra and some which only require the knowledge of the presence of some signal rather than complete reconstruction

Reply: The authors appreciate the reviewer's comments. We sincerely appreciate your constructive comments, which help to clarify the time consumption mechanism and performance trade-off of our system.

The 4-second reconstruction time reported in this paper is entirely attributed to the computational process of the spectral reconstruction algorithm, distinct from the optical signal acquisition time. Specific explanations are as follows: The system is solid-state architecture with no moving parts, and the adopted thermal modulation technology has a rate of up to 10 kHz. Thus, the actual optical signal acquisition process can be fast. For computationally reconstructed spectrometers, the computing time of the algorithm is the core source of the total system time consumption, which is mainly determined by the number of sampling channels (m) and the number of spectral channels (n). Given that our system adopts an oversampling design to ensure reconstruction accuracy, in practical applications, the computational speed can be significantly improved by reducing the number of sampling channels involved in reconstruction, achieving a flexible trade-off between accuracy and speed within an acceptable precision range.

In response to your request, we have added relevant content in the main text and supplementary note 14. Fig. **R22** demonstrates the corresponding relationships between reconstruction accuracy and the number of sampling channels, clearly presenting the performance trade-offs under different channel number configurations. For spectra dominated by smooth features, reconstruction can be achieved with a small number of detections, while for sparse spectra, increasing the number of detections can significantly reduce the reconstruction error. Furthermore, while reducing the number of sampling channels decreases the reconstruction time, our results demonstrate that the subspace-based alternating optimization method is relatively insensitive to increases in matrix dimensionality. As shown in Fig. **R23**, the reconstruction time remains consistently in the order of seconds.

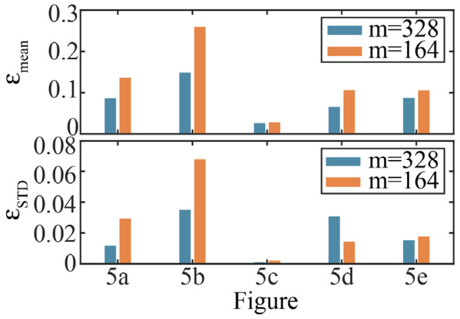

Fig. R22 | The mean ε_{mean} and standard deviation ε_{STD} of reconstruction error based on Fig. 5 presented in the main text.

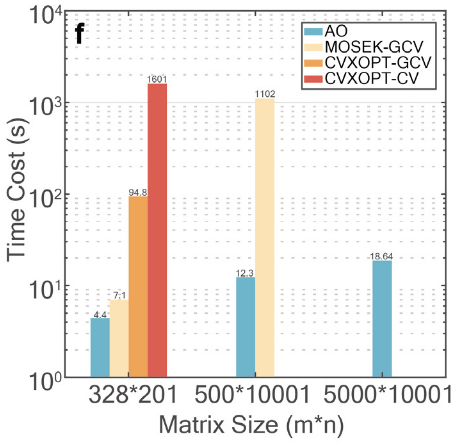

Fig. R23 | Reconstruction efficiency of different methods for different matrix dimensions. For larger-scale problems, the reconstruction time of our alternating optimization does not increase significantly. Direct solution via convex optimization was implemented using CVXPY (solver: CVXOPT and MOSEK), while the alternating optimization method was carried out with the IRtools. Calculations were all performed on a laptop with a 10-core Apple M1 Pro CPU and 16 GB of RAM. AO: alternating optimization; MOSEK-GCV: convex optimization with MOSEK backend with GCV; CVXOPT-GCV: convex optimization with CVXOPT backend with GCV; CVXOPT-CV: convex optimization with CVXOPT backend with 10-fold-CV.

Changes made to the manuscript:

Supplementary Note 14

14. Reconstruction Consistency

To evaluate the reliability of the results, we performed an additional five sampling reconstructions for each case in the main text and calculated the mean ε_{mean} and standard deviation ε_{STD} of the reconstruction errors across these five independent reconstructions. In addition to our standard sampling (m = 328), we also adopted an under-sampling scheme for spectral reconstruction (m = 164). As illustrated in Fig. **S21**, ε_{mean} of narrow-band spectra (5a, 5b) are more sensitive to the number of channels, and finer sampling results in lower narrow-band reconstruction errors. In contrast, the reconstruction errors of broadband spectra (5c) are less sensitive to the number of sampling channels.

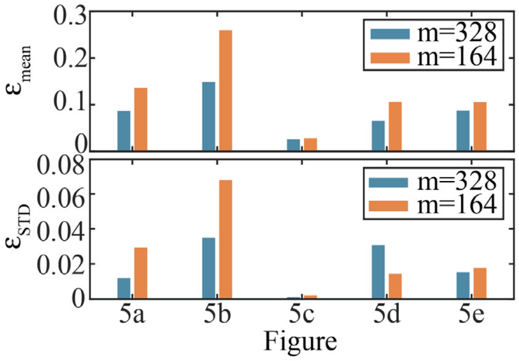

Fig. S21. The mean ε_{mean} and standard deviation ε_{STD} of reconstruction error based on Fig. 5 presented in the main text.

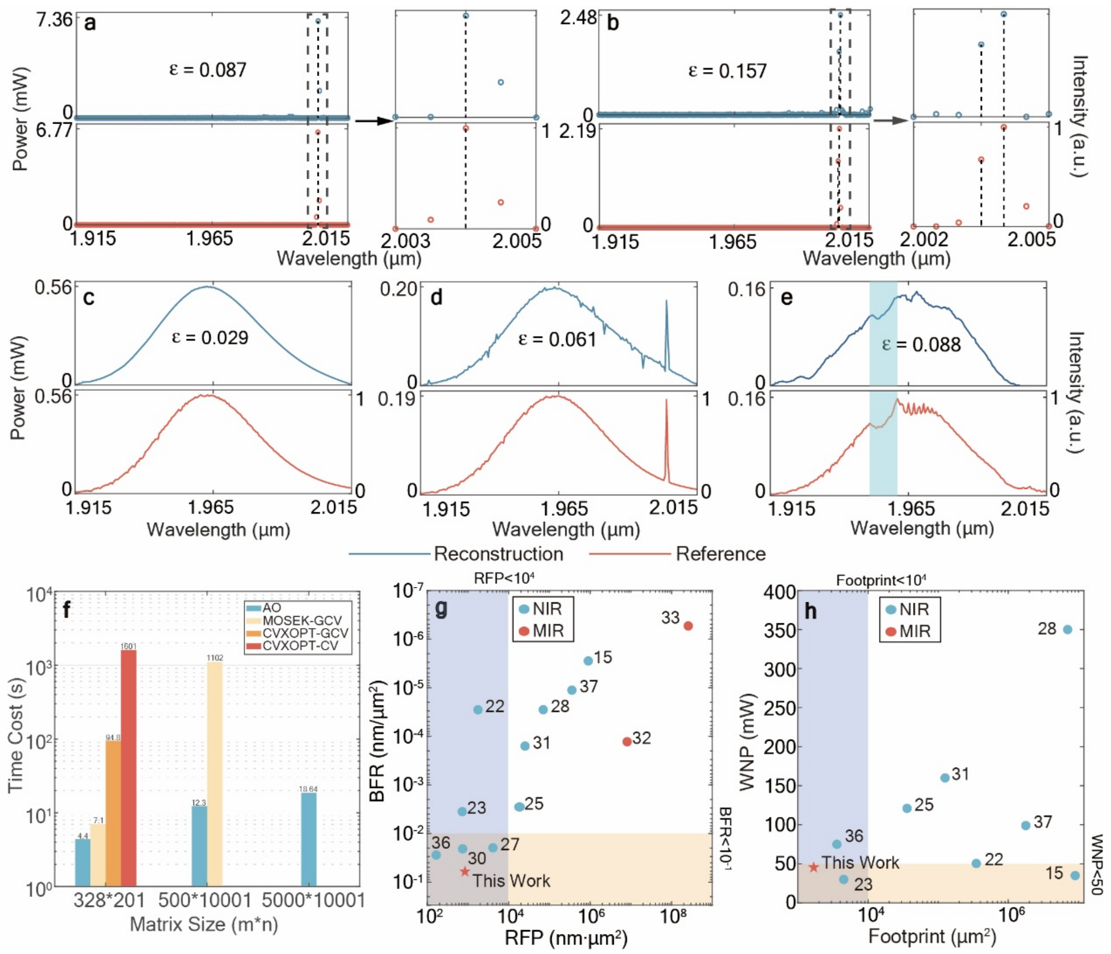

Fig. 5 | Reconstruction results and performance comparison. a-e The blue line is the algorithm reconstruction result, and the red line is the reference measured by the commercial spectrometer. **a** Single peak input spectrum and its reconstructed result. The peak is at 2004 nm. The right side shows an enlargement of the left side, which exhibits a full width at half maximum (FWHM) of 0.5 nm. **b** Dual peaks reconstructed result. An additional peak is set at 2003.5 nm adjacent to the 2004 nm peak. The right side displays a zoomed-in view of the left side. Compared to the **a**, the reconstruction result displays the additional peak line. **c** Broadband ASE input spectrum and its reconstructed result. **d** The input hybrid spectrum, which combines the ASE and a laser at 2004 nm. **e** Spectrum of ASE after absorption by carbon dioxide and its reconstruction result. The blue region represents the reconstructed absorption envelope. **f** Reconstruction efficiency of different methods for different matrix dimensions. For larger-scale problems, the reconstruction time of our alternating optimization does not increase significantly. Direct solution via convex optimization was implemented using CVXPY (solver: CVXOPT and MOSEK), while the alternating optimization method was carried out with the IRtools. Calculations were all performed on a laptop with a 10-core Apple M1 Pro CPU and 16 GB of RAM. AO: alternating optimization; MOSEK-GCV: convex optimization with MOSEK backend with GCV; CVXOPT-GCV: convex optimization with CVXOPT backend with GCV; CVXOPT-CV: convex optimization with CVXOPT backend with 10-fold-CV.The spectrometer performance is compared with the focus on **g** resolution-footprint product (RFP) and bandwidth-footprint ratio (BFR), **h** wavelength-normalized-power (WNP) and footprint.

Q14: Again, the idea of optical throughput (or efficiency), the authors state: “the system is readily scalable by adding more MPCW channels with varied lattice constants”, which is true, but this then divides the same optical power (which is low anyway) to more separate channels which is inherently more lossy. Therefore, how does this power scale? For example, does 1 more channel result in increased reconstruction time? Less reconstruction time but the tradeoff is power loss? If so, but how much and what is the balance here?

Reply: The authors appreciate the reviewer’s comments. We fully acknowledge the reviewer’s assessment: when the system is scaled by adding more MPCW channels with different lattice constants, there is indeed an optical power loss problem. The primary cause is that the incident optical power is split into more independent channels, resulting in a significant reduction in the optical throughput per channel—a common bottleneck in multi-channel on-chip spectrometer scaling.

To address this power loss issue, we propose a corresponding solution: additional wavelength division multiplexers (WDM) and optical switches are integrated on chip. The optical routing function is realized to intelligently distribute the incident light—instead of splitting the incident light into all channels simultaneously, the optical switch selects the channels adapted to specific wavebands according to the actual spectral detection needs, so that the incident optical power is concentrated on the selected channels. This effectively reduces the power loss caused by multi-channel splitting and ensures the optical throughput of a single working channel.

Regarding the impact of multi-channel scaling on reconstruction time: it should first be noted while an increased number of channels adds to the computational load, the subspace-based alternating optimization method ensures that the system remains scalable. The reconstruction time exhibits only a sub-linear growth rather than an order-of-magnitude increase, allowing the time cost to remain well-controlled. In addition, our system has flexible performance adjustment capabilities: without any modifications to the hardware structure, the reconstruction time can be significantly reduced by dynamically reducing the number of sampling channels involved in reconstruction during the sampling stage, at the cost of a small amount of spectral resolution. Thus, a flexible balance among "power loss, reconstruction time, and spectral resolution" is achieved, adapting to the needs of different application scenarios.

Q15: I couldn’t see any mention of polarization sensitivity but assume this is a critical parameter too – could you briefly indicate how the polarization control is maintained?

Reply: The authors appreciate the reviewer’s comments. In photonic integrated circuits, grating couplers are essential components for coupling off-chip light into the chip. Based on our prior work [*(s*, **31**, 39079–39087 (2023)], the proposed TE-polarized grating coupler exhibits exceptional polarization selectivity. The experimental characterization is presented in Fig. **R24**. The laser source (OELTS-300) output power was measured at approximately 9.81 dBm with a power fluctuation of 3.11%. When the input polarization matches the grating (TE mode), the coupled optical power remains stable at -5.82 dBm with a fluctuation of 4.23%. Conversely, when the input is perpendicular to the grating's preferred direction (TM mode), the coupled power drops to -37.5 dBm. Consequently, the grating coupler demonstrates a high polarization extinction ratio exceeding 32 dB, ensuring that the TM mode has a negligible impact on our experimental results. Furthermore, our MPCW structure itself **exclusively supports the TE mode**, providing an additional layer of filtering polarization. In our experiments, the ASE source is unpolarized, while the polarization state

of the laser is precisely governed by a manual polarization controller to ensure alignment with the TE mode of the chip.

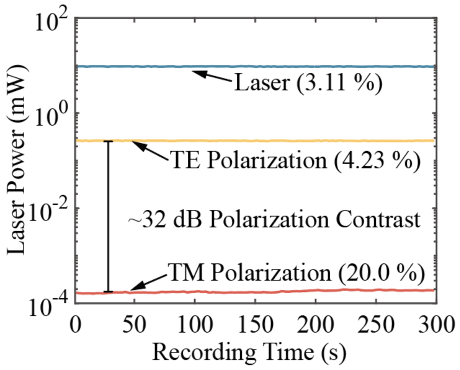

Fig. R24 | Stability of the laser output power (blue) and the corresponding output optical power after inputting TE polarization (yellow) and TM polarization (red) into the reference grating. The polarization contrast ratio of the grating is > 32 dB.

Changes made to the manuscript:

Supplementary Note 7

7. Polarization Stability

During the experiment, we also used a polarization controller to adjust the polarization state of the input light, ensuring that the polarization of light passing through the grating coupler always matched the designed polarization direction of the grating. We separately recorded the laser output power, as well as the output power when TE-polarized and TM-polarized light was injected into the reference waveguide. Our grating coupler is a TE-polarized coupler, and the measurement results are shown in Fig. **S13**.

It can be seen that the laser output power is around 9.81 dBm (OELTS-300), with a power fluctuation of 3.11%. When the input light polarization matches the grating (TE polarization input), the optical power is stable at −5.82 dBm, with an output power fluctuation of 4.23%. When the input polarization is perpendicular to the selected polarization direction of the grating (TM polarization input), the output optical power is −37.5 dBm. Therefore, our grating coupler exhibits high polarization selectivity, with a polarization contrast ratio higher than 32 dB, and TM polarization does not influence our experiment. In addition, our MPCW only supports TE mode.

To ensure the power stability of the MPCW spectrometer, we fixed the transmission fiber using tape during the experiment to avoid excessive bending and torsion of the fiber, which would induce polarization changes and affect the response and output optical power of the MPCW.

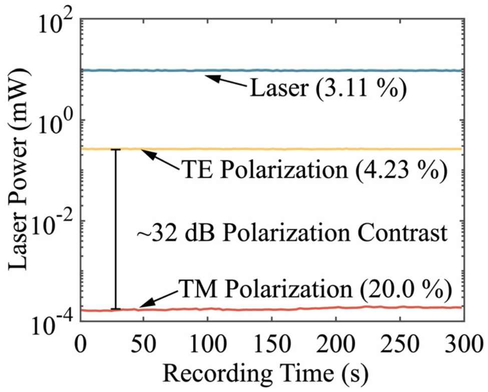

Fig. **S13**. Stability of the laser output power (blue) and the corresponding output optical power after inputting TE polarization (yellow) and TM polarization (yellow) into the reference grating. The polarization contrast ratio of the grating is > 32 dB.

Q16: MEMS FTIR [Hamamatsu light engine] comparison: I can currently buy a small MEMS FTIR which covers 1.1 to 2.5 um, with spectral resolution down to 5 nm (but can be adjusted). The cost will be cheaper, throughput higher, bandwidth much larger etc. And when comparing total package volume and weight, I suspect not too dissimilar to the PIC approach (when combined with coupling optics, TEC etc.). Therefore, can you please compare and contrast to actual commercial spectrometers? Because the state of the art is not just what researchers are doing but what one can actually buy and I'd like to know the potential of the architecture here compared to what's commercially available and what need your approach is satisfying.

Reply: The authors appreciate the reviewer's comments. Our spectrometer is mainly based on PIC architecture. Compared with the commercial spectrometers you mentioned, our device is more tailored for specialized on-chip integration rather than general-purpose standalone instruments. Although our spectrometer was fabricated using EBL, large-scale PIC production with photolithography can significantly reduce the cost. In contrast, the electronic peripheral components currently dominate the total footprint. Nevertheless, the core structure of our spectrometer is compact and allows monolithic integration with other functional photonic devices, such as sensors or detectors, to form integrated functional systems.

In addition, we have looked into the structure of the spectrometer you mentioned, which is an FTIR based on a MEMS movable mirror design. Compared with this structure, our spectrometer contains solid-state architecture with no mechanical moving parts, and the single SOI chip is thinner, making it more suitable for integration into portable equipment. Combined with the subspace-based alternating optimization algorithm, the computation can be performed on edge devices such as mobile phones.

Admittedly, compared with the commercial spectrometers you mentioned, our prototype has certain performance limitations, for example, in achieving a wider bandwidth. This is primarily because the waveguide dispersion is inherently more pronounced than in free-space optics, which imposes a bandwidth constraint on integrated photonic devices. However, owing to the flexible device design, our spectrometer can achieve a finer spectral resolution.

Overall, the two types of spectrometers target different application scenarios. We present a table here for a direct comparison with the commercial spectrometer you suggested.

Feature	FTIR light engine	MPCW spectrometer
Internal optical path	Free space	Optical waveguide
Moving parts	Yes	No
Vibration sensitivity	Sensitive	Insensitive
Temperature sensitivity	Relatively sensitive	Sensitive
Measurement speed	Second scale (moving unit)	Tens of milliseconds (micro-heater)
Computational	Simple	Moderate (subspace method)

complexity		
Spectral resolution	Moderate<br>(5 nm@1.1 μm–15 nm@2.5 μm)	High<br>(0.5 nm@2 μm)
Spectral bandwidth	Large	Moderate
TEC requirement	Not mentioned	Required
Detector	External	Short wavelength: on-chip integration (SiGe);<br>long wavelength: additional bonding process required
Control unit	FPGA	FPGA or microcontroller
System volume	Optical chip is compact and thin, integrable with some smart devices; overall volume similar due to peripheral circuits	

Reviewer 3:

The authors present a mid-infrared on-chip computational spectrometer based on microcavity-coupled photonic crystal waveguides (MPCWs), featuring a tailored reconstruction algorithm and thermo-optic tuning. While the work demonstrates careful engineering and achieves competitive specifications (0.5 nm resolution over 100 nm bandwidth around 2 μm), I find that the study does not meet the high bar of novelty, broad interest, or conceptual advancement required for publication in Nature Communications. I therefore recommend rejection in its current form. Below are my major concerns:

We appreciate the reviewer's comments and have addressed the concern raised below.

Q1: The core architecture—combining photonic crystal waveguides with side-coupled cavities and thermal tuning—has been extensively explored in the near-infrared regime. While the translation to the mid-infrared (MIR) is valuable, the manuscript does not introduce a fundamentally new operating principle or physical mechanism. The improvements (e.g., non-uniform FSR, slow-light enhanced tuning) appear as logical extensions of existing photonic crystal concepts rather than a transformative step. For Nature Communications, the work would need to demonstrate a more paradigm-shifting innovation or a breakthrough in performance far beyond the state-of-the-art.

Reply: The authors appreciate the reviewer's comments. We agree that the fundamental building blocks, including photonic crystal waveguides, side-coupled cavities, and thermal tuning, have been widely investigated in the near-infrared regime, and this work does not introduce fundamentally new physical mechanisms. However, we would like to emphasize that the core contributions of this manuscript lie not in proposing a disruptive new physics, but in paradigm-shifting advances at the system and application levels, which we summarize as follows:

1. Extension to the MIR Spectral Range: We have successfully adapted and optimized the photonic crystal platform for the MIR regime. This effort establishes a reusable design and integration paradigm for on-chip MIR spectrometers and validates the platform's scalability to longer wavelengths.
2. Synergistic Architecture for Performance Enhancement: We present a synergistic design that combines a non-uniform FSR with slow-light-enhanced tuning. This configuration enables higher spectral resolution and a broader operating bandwidth while utilizing a significantly limited number of physical channels.

3. Efficient Computational Framework: We introduce an alternating optimization scheme for spectral reconstruction, which accelerates the convergence of the solution process. Combined with the automated regularization parameter selection, this framework significantly reduces the computational burden.

We believe such system-level synergy hold great potential for advancing mid-infrared spectrometers.

Q2: The manuscript focuses heavily on device design and spectral reconstruction under controlled conditions but lacks a compelling application demonstration. MIR spectroscopy is motivated by strong molecular absorption for sensing and diagnostics; however, the authors only test the system with simple laser lines and an ASE source. There is no validation using actual MIR-relevant samples (e.g., chemical vapors, biomolecules) to showcase the claimed advantages for gas sensing, environmental monitoring, or biomedical diagnostics. Without such evidence, the practical impact remains speculative.

Reply: The authors appreciate the reviewer's comments. The point raised regarding application-specific validation is indeed crucial. Given that absorption spectroscopy is a primary application in the MIR regime, we have conducted an additional experiment. Specifically, we constructed a prototype 1-m-long absorption cell and utilized two collimated fibers to facilitate the coupling and propagation of the ASE source through the cell. Due to the lack of precise flow control in our gas delivery system, the concentration exhibited pronounced fluctuations during the sampling period. Consequently, the resulting reconstruction error is higher compared to that of a stable ASE spectrum. Nevertheless, the characteristic absorption envelope of is clearly discernible in the reconstructed spectrum, as shown in Fig. **R25e**.

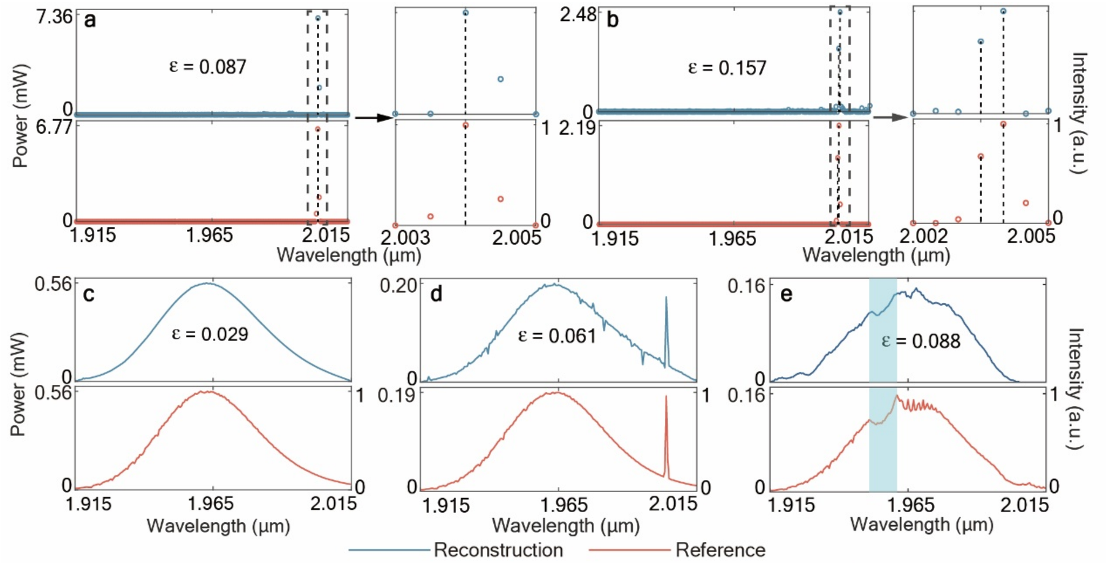

Fig. R25 | a-e The blue line is the algorithm reconstruction result, and the red line is the reference measured by the commercial spectrometer. **a** Single peak input spectrum and its reconstructed result. The peak is at 2004 nm. The right side shows an enlargement of the left side, which exhibits a full width at half maximum (FWHM) of 0.5 nm. **b** Dual peaks reconstructed result. An additional peak is set at 2003.5 nm adjacent to the 2004 nm peak. The right side displays a zoomed-in view of the left side. Compared to the a, the reconstruction result displays the additional peak line. **c** Broadband ASE input spectrum and its reconstructed result. **d** The input hybrid spectrum, which combines the ASE and a laser at 2004 nm. **e** Spectrum of ASE after absorption by carbon dioxide and its

reconstruction result. The blue region represents the reconstructed absorption envelope.

Changes made to the manuscript:

In addition, we constructed a prototype 1-m-long carbon dioxide gas cell. Following the alignment of two collimated fibers, the 2 μm ASE light source was coupled into the gas cell to acquire the absorption spectrum. Due to the lack of precise flow control in the carbon dioxide delivery system, the gas concentration fluctuated during the sampling process, leading to a degradation in reconstruction fidelity. The carbon dioxide absorption envelope remains clearly discernible in the reconstructed spectrum in Fig. 5e.

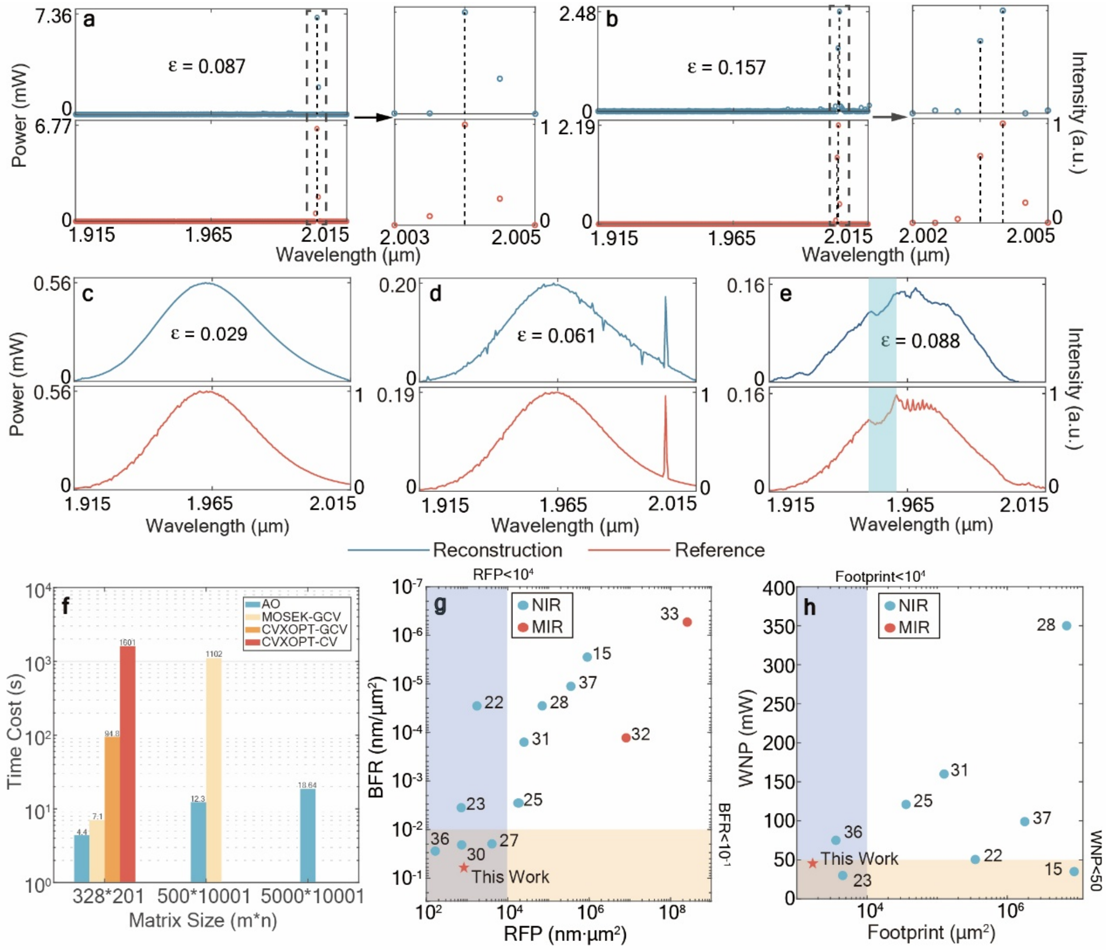

Fig. 5 | Reconstruction results and performance comparison. a-e The blue line is the algorithm reconstruction result, and the red line is the reference measured by the commercial spectrometer. **a** Single peak input spectrum and its reconstructed result. The peak is at 2004 nm. The right side shows an enlargement of the left side, which exhibits a full width at half maximum (FWHM) of 0.5 nm. **b** Dual peaks reconstructed result. An additional peak is set at 2003.5 nm adjacent to the 2004 nm peak. The right side displays a zoomed-in view of the left side. Compared to the **a**, the reconstruction result displays the additional peak line. **c** Broadband ASE input spectrum and its reconstructed result. **d** The input hybrid spectrum, which combines the ASE and a laser at 2004 nm. **e** Spectrum of ASE after absorption by carbon dioxide and its reconstruction result. The blue region represents the reconstructed absorption envelope. **f** Reconstruction efficiency of different methods for different matrix dimensions. For larger-scale problems, the reconstruction time of our alternating optimization does not increase significantly. Direct solution via convex optimization was implemented using CVXPY (solver: CVXOPT and MOSEK), while the alternating optimization

method was carried out with the IRtools. Calculations were all performed on a laptop with a 10-core Apple M1 Pro CPU and 16 GB of RAM. AO: alternating optimization; MOSEK-GCV: convex optimization with MOSEK backend with GCV; CVXOPT-GCV: convex optimization with CVXOPT backend with GCV; CVXOPT-CV: convex optimization with CVXOPT backend with 10-fold-CV.The spectrometer performance is compared with the focus on **g** resolution-footprint product (RFP) and bandwidth-footprint ratio (BFR), **h** wavelength-normalized-power (WNP) and footprint.

Q3: The performance comparison (Figs. 5f–5g) selectively highlights metrics where the device excels (e.g., footprint-related ratios) but overlooks other critical parameters such as absolute accuracy, dynamic range, or power consumption relative to prior MIR on-chip spectrometers. The claimed 0.5 nm resolution is demonstrated with a single laser peak but not rigorously validated across the entire band or under realistic noisy conditions (Fig. 3e only shows simulation). Additionally, the algorithm's speed advantage, while notable, is not benchmarked against other recent advanced reconstruction methods beyond basic convex optimization.

Reply: The authors appreciate the reviewer's comments. We acknowledge that the initial comparison focused primarily on device footprint and resolution, while metrics such as absolute accuracy and dynamic range were not exhaustively discussed. It is noteworthy that in the burgeoning field of computational on-chip spectrometers, standardized reporting of absolute accuracy and dynamic range remains less common than traditional metrics like spectral resolution, bandwidth, and footprint. Additionally, the literature on MIR on-chip spectrometers is still limited, with the majority of reported devices being passive and lacking active tuning mechanisms or detailed system-level power analysis, as shown in the following table. The MEMS based photonics spectrometer [*ACS Photonics* **9**, 2367–2377 (2022)], the driving voltage used in the reported MEMS device is as high as 80 V, while the maximum voltage for our thermal modulation is only around 4.2 V.

Structure	Footprint (μm^2)	WB (μm)	RES (nm)	BW (nm)	CH	Active?	WNP (mW)	RFP (nm·μm^2)	BFR (nm·μm^{-2})
**FTS Array[12]**	NA	MIR (3.30)	12	60	12	N	/	/	/
**FTS Array[13]**	9.5×10^7	MIR (3.75)	2.7	51	42	N	/	2.57×10^8	5.37×10^{-7}
**Waveguide Coupler[14]**	2.7×10^6	MIR (3.85)	3	350	1	Y	NA	8.1×10^6	1.29×10^{-4}
**MPCW (This work)**	**1680**	**MIR (2.00)**	**0.5**	**100**	**8**	**Y**	**45.6**	**840**	**0.06**

WB: waveband, RES: resolution, BW: bandwidth, CH: channel, WNP: wavelength-normalized power, RFP: resolution-footprint product, BFR: bandwidth-footprint ratio.

We sincerely appreciate your rigorous comment on the validation of the claimed 0.5 nm spectral resolution. We agree that demonstrating resolution with only a single laser peak is insufficient to verify performance across the entire operating band and under realistic noisy conditions. Due to the limited operating bandwidth of the laser (OELTS-300, 1970–2070 nm), we conducted evaluations using peaks at different wavelengths within the 1970–2015 nm range and compared the results with those measured by the OSA (Fig. **R26**). This systematically verified that a resolution of 0.5 nm can

be stably achieved across the most working range, rather than only being effective at a single wavelength point. In addition, the laser itself exhibits power instability, which inherently introduces a certain amount of random noise.

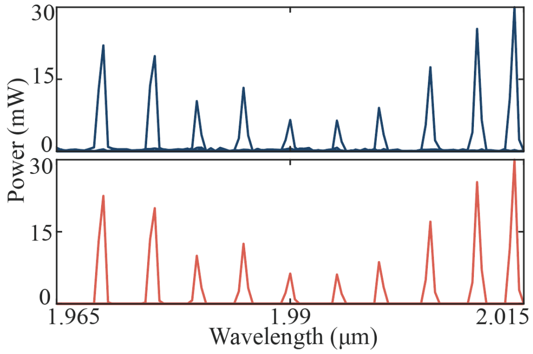

Fig. R26 | Reconstructed spectrum of the tunable laser in the range of 1970–2015 nm. The red curve denotes the reference spectrum, and the blue curve represents the reconstruction result.

We sincerely appreciate your comments on reconstruction algorithms. Over the past few years, the mathematical framework for computational spectrometers has predominantly centered on convex optimization schemes to address the underlying inverse problems. [*Nat Commun* **12**, 2704 (2021); *Nat Commun* **14**, 6376 (2023); *Laser & Photonics Reviews* **17**, 2200792 (2023); *Light Sci Appl* **12**, 156 (2023); *Nat Commun* **9**, 4405 (2018); *ACS Photonics* **12**, 5413–5421 (2025); *ACS Photonics* **12**, 1849–1856 (2025); *Light Sci Appl* **14**, 331 (2025)]. However, the point you raised is crucial, as we have observed that even when different solvers are employed, the computational latency varies due to their distinct built-in algorithms (e.g., interior-point method, ADMM, etc.). To provide a rigorous benchmark, we compared our proposed algorithm against established solvers with varying efficiencies, specifically the CVXOPT solver (standard efficiency) and the MOSEK solver (high efficiency). Additionally, since many studies on computational spectrometers either omit the details of regularization parameter selection, rely on empirical tuning, or employ standard K-fold cross-validation (CV), we believe a more refined comparison is necessary. Consequently, we specifically designed a relatively universal GCV scheme tailored to similar optimization objectives to automatically select regularization parameters. **We would like to kindly clarify that, to the best of our knowledge, no existing work on computational reconstruction spectrometers has proposed a dual-parameter optimization scheme for simultaneously selecting L1 and L2 regularization parameters to date. Thus, our work essentially develops an optimized scheme building upon prior studies, and we compared this optimized scheme with the alternating optimization approach.** Then, we compared the average reconstruction speed of different methods using the test cases presented in Figs. 5a-5e. as shown in Fig. **R27**. Additionally, we also examined the computation time of these methods for larger-scale calibration matrices. The time shown in Fig. **R27** includes the parameter search duration. These methods include the direct solution [Eq. (3)] via convex optimization (using CVXOPT and MOSEK solvers) which searches for regularization parameters through 10-fold CV and GCV methods, and the alternating optimization method with automatic parameter selection. For convex optimization, it is worth noting that parameter searching in CV is time-consuming. The GCV methods in convex optimization perform well when the matrix size is small. However, as the size of the calibration matrix increases, the overhead of the optimization process and the computational cost of the GCV function calculation increases

significantly, leading to an increase in the overall computation time. For alternating optimization, since the problem is solved within the projected subspace, the computation time does not increase significantly. Moreover, since the GCV value is computed within subspace, the computational burden is significantly reduced. Overall, the alternating optimization scheme offers rapid computation and exhibits excellent scalability, as the computational latency does not increase significantly with higher dimensionality. This characteristic is particularly advantageous for the future expansion of the spectrometer's measurement range.

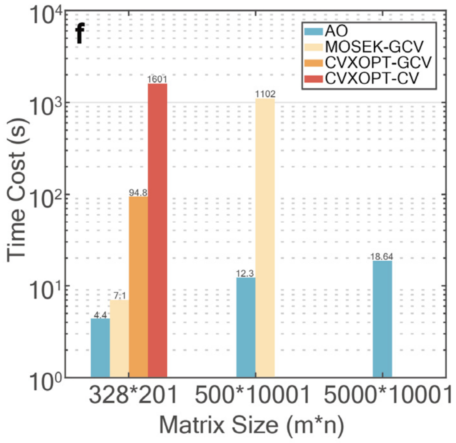

Fig. R27 | Reconstruction efficiency of different methods for different matrix dimensions. For larger-scale problems, the reconstruction time of our alternating optimization does not increase significantly. Direct solution via convex optimization was implemented using CVXPY (solver: CVXOPT and MOSEK), while the alternating optimization method was carried out with the IRtools. Calculations were all performed on a laptop with a 10-core Apple M1 Pro CPU and 16 GB of RAM. AO: alternating optimization; MOSEK-GCV: convex optimization with MOSEK backend with GCV; CVXOPT-GCV: convex optimization with CVXOPT backend with GCV; CVXOPT-CV: convex optimization with CVXOPT backend with 10-fold-CV.

Changes made to the manuscript:

Supplementary Note 11

11. Spectral Reconstruction of Lasers at Different Wavelengths

Since our tunable laser (OELTS-300) operates in the range of 1970–2070 nm, which does not fully cover the operating range of our spectrometer. Thus, we reconstructed the laser spectrum within the range of 1970–2015 nm. The reconstruction results are shown in Fig. **S18**.

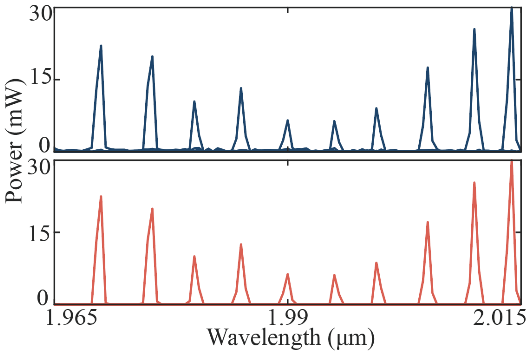

Fig. **S18**. Reconstructed spectrum of the tunable laser in the range of 1970–2015 nm. The red curve denotes the reference spectrum, and the blue curve represents the reconstruction result.

Main Text

Additionally, we compared the average reconstruction speed of different methods (Fig. 5f) using the test cases presented in Figs. 5a-5e. Furthermore, we also examined the computation time of these methods for larger-scale calibration matrices. The time shown in Fig. 5f includes the parameter search duration. These methods include the direct solution [Eq. (3)] via convex optimization (using CVXOPT and MOSEK solvers) which searches for regularization parameters through K-fold CV and GCV methods (K=10, see Supplementary Note 15 for details on CV and GCV), and the alternating optimization method with automatic parameter selection. For convex optimization, it is worth noting that parameter searching in CV is time-consuming. Reducing the search time may lead to failing to identify the optimal regularization parameters, which in turn results in larger reconstruction errors compared to the alternating optimization method. The GCV methods in convex optimization perform well when the matrix size is small. However, as the size of the calibration matrix increases, the overhead of the optimization process and the computational cost of the GCV function calculation increases significantly, leading to an increase in the overall computation time. For alternating optimization, since the problem is solved within the projected subspace, the computation time does not increase significantly. Moreover, since the GCV value is computed within subspace, the computational burden is significantly reduced.

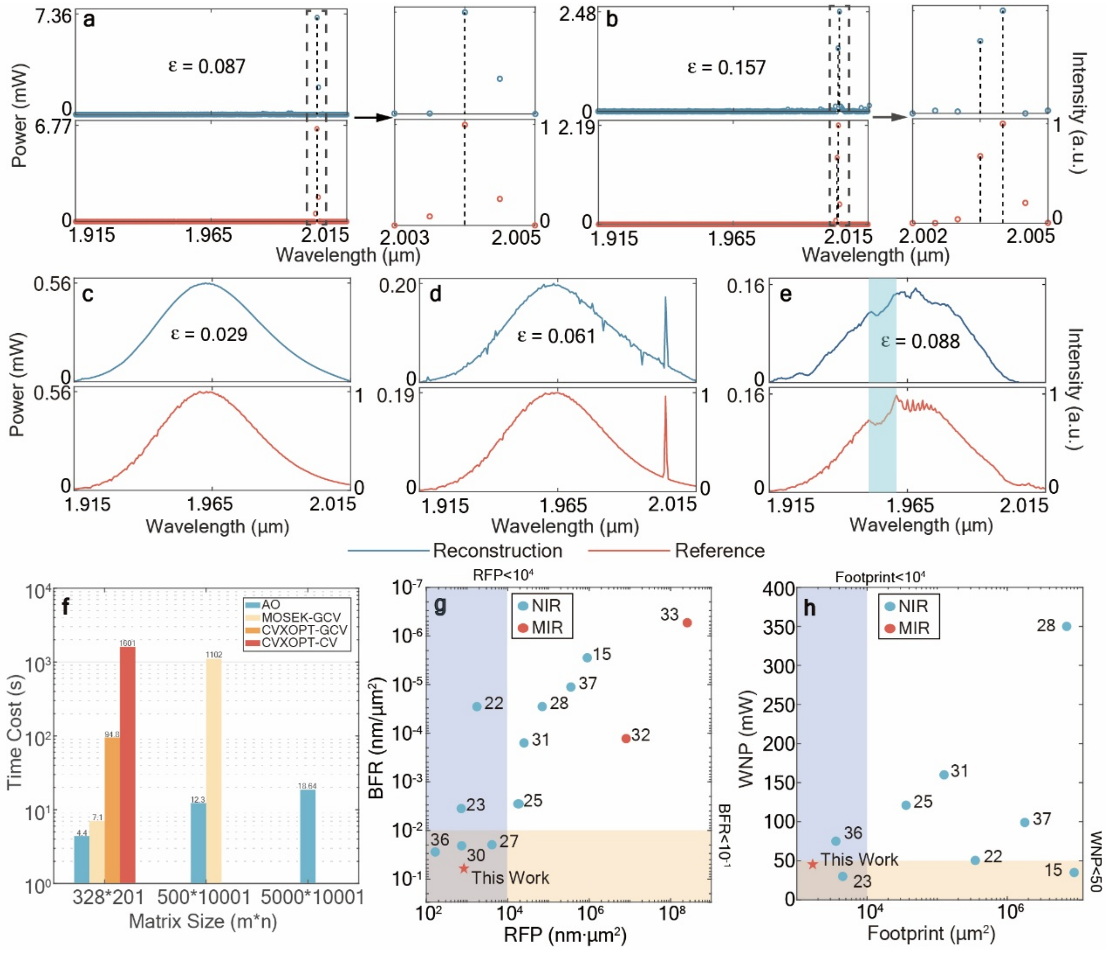

Fig. 5 | Reconstruction results and performance comparison. a-e The blue line is the algorithm reconstruction result, and the red line is the reference measured by the commercial spectrometer. **a** Single peak input spectrum and its reconstructed result. The peak is at 2004 nm. The right side shows an enlargement of the left side, which exhibits a full width at half maximum (FWHM) of 0.5

nm. **b** Dual peaks reconstructed result. An additional peak is set at 2003.5 nm adjacent to the 2004 nm peak. The right side displays a zoomed-in view of the left side. Compared to the **a**, the reconstruction result displays the additional peak line. **c** Broadband ASE input spectrum and its reconstructed result. **d** The input hybrid spectrum, which combines the ASE and a laser at 2004 nm. **e** Spectrum of ASE after absorption by carbon dioxide and its reconstruction result. The blue region represents the reconstructed absorption envelope. **f** Reconstruction efficiency of different methods for different matrix dimensions. For larger-scale problems, the reconstruction time of our alternating optimization does not increase significantly. Direct solution via convex optimization was implemented using CVXPY (solver: CVXOPT and MOSEK), while the alternating optimization method was carried out with the IRtools. Calculations were all performed on a laptop with a 10-core Apple M1 Pro CPU and 16 GB of RAM. AO: alternating optimization; MOSEK-GCV: convex optimization with MOSEK backend with GCV; CVXOPT-GCV: convex optimization with CVXOPT backend with GCV; CVXOPT-CV: convex optimization with CVXOPT backend with 10-fold-CV.The spectrometer performance is compared with the focus on **g** resolution-footprint product (RFP) and bandwidth-footprint ratio (BFR), **h** wavelength-normalized-power (WNP) and footprint.

Supplementary Note 15

15. Selection of Regularization Parameters in Convex Optimization.

In the spectral reconstruction process of a computational spectrometer, cross-validation (CV) primarily partitions the detection channels of the calibration matrix (i.e., the rows of the matrix) into a reconstruction part (rp) and a validation part (vp). Spectral reconstruction is performed using the calibration results and detected power of the reconstruction part channels ($\mathbf{T}_{rp}$ and $\mathbf{d}_{rp}$) and evaluates the quality of regularization parameters by comparing the error between the predicted power ($\mathbf{T}_{vp}\hat{\mathbf{s}}_{rp}$), obtained by applying the reconstructed spectrum ($\hat{\mathbf{s}}_{rp}$) to the validation part channels, and the measured power $\mathbf{d}_{vp}$ of the validation part. This error can be expressed as:

$$\frac{1}{N_{vp}}\sum_{vp}\left(\mathbf{T}_{vp}\hat{\mathbf{s}}_{rp}-\mathbf{d}_{vp}\right)^2$$

N_{rp} and N_{vp} denote the number of channels in the reconstruction part and validation part, respectively.

The indices of all channels are shuffled, then partitioned into ten groups, each of which serves as the validation part in turn. For each validation group, the indices not included in the validation part form the reconstruction part. For each regularization parameter, the CV error of each group is calculated, and the optimal parameters are selected as those corresponding to the minimum CV error. In practical implementation, we noticed that the computational time required for K-fold CV is excessively long. Additionally, we introduce a generalized cross-validation (GCV) function for selecting regularization parameters, which references the GCV function used in the subspace iteration method, though this GCV function is approximate for L1 regularization parameter selection. The GCV function is defined as follows:

$$G(\alpha,\beta)=\frac{\|\mathbf{r}(\alpha,\beta)\|_2^2}{\left(trace(\mathbf{I}-\mathbf{T}(\mathbf{T}^{\dagger}\mathbf{T}+\alpha^2\,\mathbf{D}^{\dagger}\,\mathbf{D}+\beta^2\,\mathbf{F}^{\dagger}\,\mathbf{F})^{-1}\mathbf{T}^{\dagger})\right)^2}$$

where $\mathbf{r}(\alpha,\beta)$ denotes the residual at regularization parameters α and β. The matrix $\mathbf{F}$ can be expressed as:

$$\mathbf{F} = \mathrm{diag}\left(\left[2\sqrt{s_i^2 + \varepsilon}\right]^{-\frac{1}{2}}\right)_{i=1}^{n}$$

The optimization procedure for the GCV method is as follows: initialize α and β, perform one round of solution using convex optimization tools, minimize the GCV function with the obtained solution, update α and β, and repeat this process until the GCV values reach a plateau.

Q4: The manuscript briefly mentions scalability to other wavelengths but does not address practical barriers to larger channel counts or broader bandwidths. The current design uses only 8 MPCW units and requires 328 calibration measurements—a process that may become cumbersome for mass fabrication or field deployment. Furthermore, the impact of fabrication variability on cavity resonances and band-edge uniformity is not quantified, nor is the long-term stability of the thermo-optic tuning under repeated cycling discussed. These aspects are critical for assessing the technology's readiness for real-world use.

Reply: The authors appreciate the reviewer's comments. We sincerely appreciate your insightful comments on scalability, calibration effort, fabrication variability, and long-term stability. These are critical aspects for evaluating the practical readiness of our technology, and we fully agree that they deserve thorough discussion. For practical barriers for scaling to larger channel counts and broader bandwidths, we agree that the current 8-MPCW design is at a proof-of-concept level. The main challenges for further scaling include optical power splitting, crosstalk, and computational complexity. However, scaling can be made practical by using on-chip wavelength division multiplexing (WDM) and optical switches to realize dynamic optical routing, thus avoiding excessive power division. Meanwhile, our subspace-based alternating optimization algorithm exhibits favorable scalability: the reconstruction time increases only mildly with the number of channels, rather than exponentially, supporting feasible extension to a larger number of channels.

The 328 calibration measurements reported represent a rigorous and comprehensive calibration procedure for laboratory validation. To transition toward mass fabrication and field deployment, this requirement can be significantly reduced. Specifically, model-driven sparse calibration and transfer learning can be applied by leveraging high consistency among fabricated devices, allowing a pre-trained model from a master device to be mapped to subsequent batches with minimal additional measurements.

For impact of fabrication variability on cavity resonances and band-edge uniformity, in general, fabrication errors mainly arise from variations in the hole diameter. We present the relationship between the diameter variation of the photonic crystal holes and the corresponding transmission spectrum shift (Fig. **R28**). A typical fabrication error of 10 nm in the hole diameter usually leads to a spectral deviation of approximately 15 nm. In practical applications, one or two additional redundant MPCW units can be employed to ensure the stability of the operating spectral range. Furthermore, spectral variations caused by fabrication errors are recorded in the calibration matrix. Since our main sampling channels are implemented based on thermally tuned physical channels, after measuring the passive-state spectra of the physical channels, the impact of fabrication errors can also be mitigated by combining model-driven methods with the similarity of thermally modulated spectral structures.

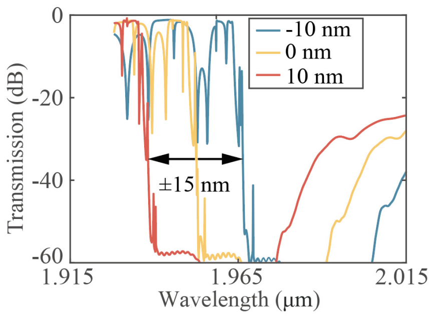

Fig. R28 | Simulation results of response spectrum variation caused by fabrication errors in photonic crystal hole diameters.

For the long-term stability of the thermo-optic tuning under repeated cycling, we evaluated the stability of the response spectrum under constant heating power (0 mW and 60 mW). Here, the average spectrum fluctuation is defined as:

$$f(t) = \frac{\left\| R(t) - R_{average} \right\|_2}{\left\| R_{average} \right\|_2}$$

where $R(t)$ is the response spectrum acquired at time t, and $R_{average}$ is the average response spectrum taken at different times. The average fluctuation measured is approximately 0.9%, as shown in Fig. **R29a**. In addition, the fluctuations of four rounds of calibration matrix measurements at 30-minute intervals is depicted in Fig. **R29b**. It can be observed that when the background temperature is stable, the average relative fluctuation of the calibration matrix is about 0.7%. These relative fluctuations can be attributed to the precision limit of TEC controller. However, compared with external noise, the error caused by such inherent temperature control fluctuations is relatively small. Using a higher-speed and more stable TEC can alleviate response fluctuations.

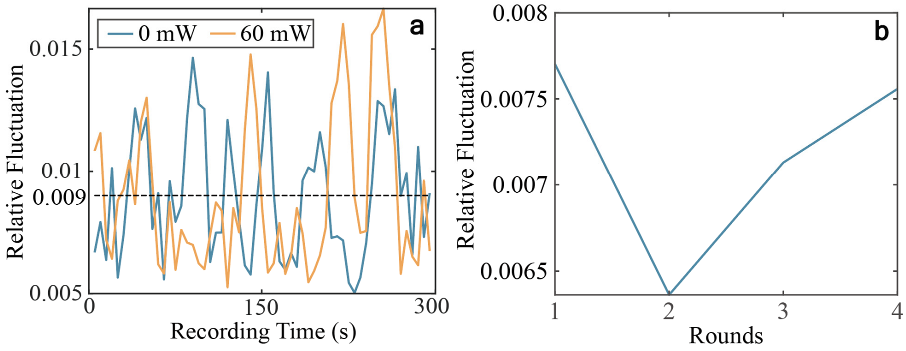

Fig. R29 | a Fluctuations in the response spectra during long-term measurements at a specific heating power **b** Fluctuations in the calibration matrix across multiple measurement runs.

Changes made to the manuscript:

Supplementary Note 5

5. Fabrication Tolerance

During fabrication, the positioning error of electron-beam lithography is very small, but the size of the exposed holes is difficult to control precisely. Therefore, we simulated the influence of hole diameter errors on the transmission characteristics of the filter. The simulation results are shown in Fig. **S8**. It can be seen that when the hole diameter error is within ±10 nm, the band-edge wavelength of the filter exhibits a red-shift or blue-shift of approximately 15 nm due to the variation in hole size. In practical mass production, photolithography is typically used for the exposure process, and the hole size can be kept relatively stable across different batches. In addition, the impact of fabrication errors on the spectrometer can be reduced by adding two extra MPCW units to cover the wavelength regions that cannot be sampled due to response shifts caused by process tolerances.

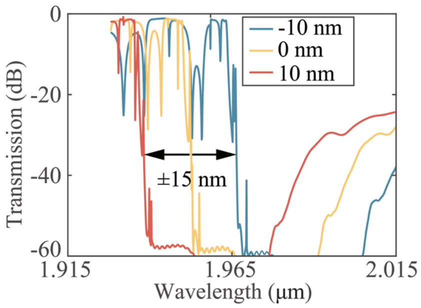

Fig. **S8**. Simulation results of response spectrum variation caused by fabrication errors in photonic crystal hole diameters.

Supplementary Note 10

10. Operation Repeatability

For the operational repeatability, we first measured the average relative fluctuation of the spectra of the MPCW element at 0 mW and 60 mW (with the TEC base temperature set to 23 °C). Similar to temperature-induced variations, the average spectrum fluctuation is defined as:

$$f(t) = \frac{\left\| R(t) - R_{average} \right\|_2}{\left\| R_{average} \right\|_2}$$

where $R(t)$ is the response spectrum acquired at time t, and $R_{average}$ is the average response spectrum taken at different times. As shown in Fig. **S17a**, the average relative fluctuation of the spectrum is approximately 0.9%, with the maximum fluctuation not exceeding 1.6%. These fluctuations are primarily attributed to the precision limit of TEC controller.

Furthermore, we performed four complete measurements of the response matrix at 30-minute intervals. In each measurement cycle, the filters underwent a full heating and cooling cycle. We calculated the relative variation of the average transmission characteristics for each channel across different measurement rounds. As shown in Fig. **S17b**, the average relative fluctuation of the entire response matrix is roughly 0.7%. This demonstrates that the designed thermo-optically controlled photonic crystal filters maintain high operational repeatability across multiple thermal tuning cycles..

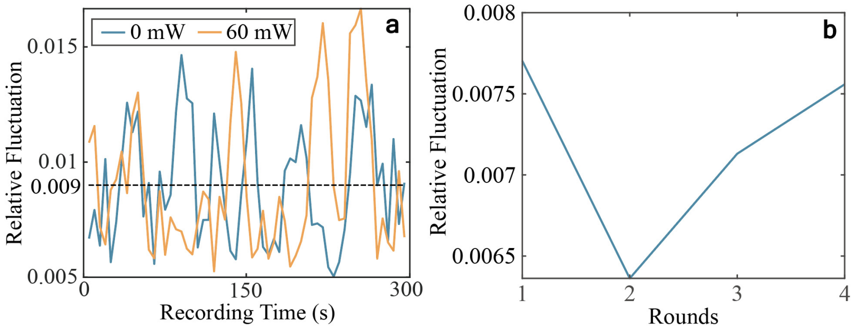

Fig. **S17**. **a** Fluctuations in the response spectra during long-term measurements at a specific heating power. **b** Fluctuations in the calibration matrix across multiple measurement runs.

Q5: The spectra for reconstruction are relatively simple, only laser peaks and broad ASE spectrum.

Reply: The authors appreciate the reviewer's comments. Same as **Q2** and **Q3**, we reconstructed spectra from broadband lasers to demonstrate the operating bandwidth of our system. In addition, light sources around the 2-μm wavelength have far fewer options compared with those in the

near-infrared band. We therefore constructed a prototype carbon dioxide gas cell to verify CO_2 absorption (Fig. **R29**). Although gas instability introduced reconstruction errors, the absorption envelope remains clearly discernible. Additionally, the lasers within 1970-2015 nm range are reconstructed (Fig. **R30**).

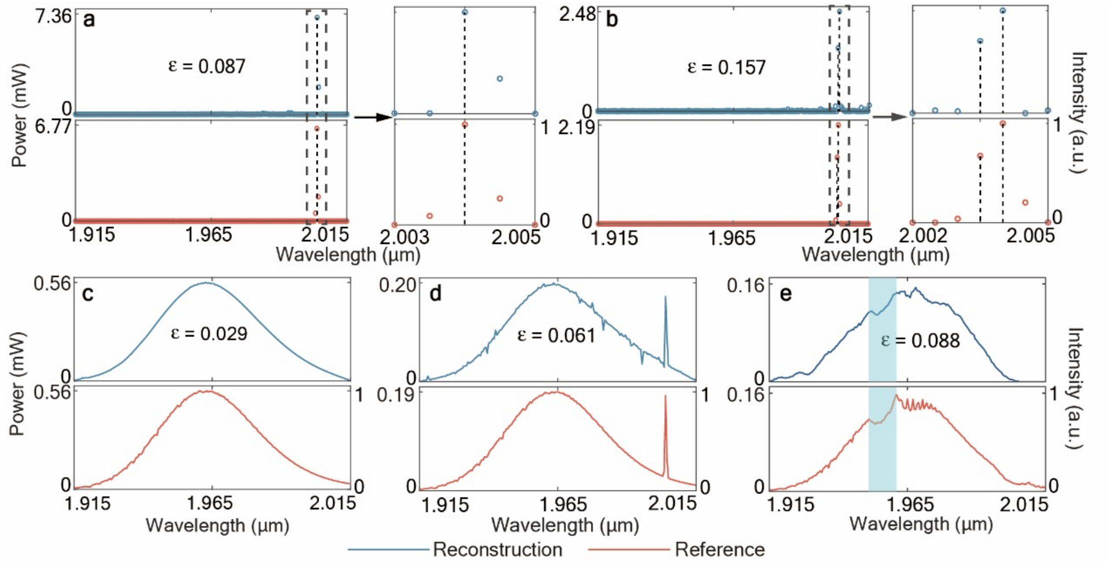

Fig. R29 | a-e The blue line is the algorithm reconstruction result, and the red line is the reference measured by the commercial spectrometer. **a** Single peak input spectrum and its reconstructed result. The peak is at 2004 nm. The right side shows an enlargement of the left side, which exhibits a full width at half maximum (FWHM) of 0.5 nm. **b** Dual peaks reconstructed result. An additional peak is set at 2003.5 nm adjacent to the 2004 nm peak. The right side displays a zoomed-in view of the left side. Compared to the a, the reconstruction result displays the additional peak line. **c** Broadband ASE input spectrum and its reconstructed result. **d** The input hybrid spectrum, which combines the ASE and a laser at 2004 nm. **e** Spectrum of ASE after absorption by carbon dioxide and its reconstruction result. The blue region represents the reconstructed absorption envelope.

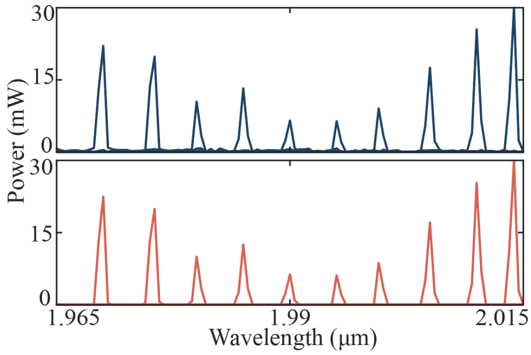

Fig. R30 | Reconstructed spectrum of the tunable laser in the range of 1970–2015 nm. The red curve denotes the reference spectrum, and the blue curve represents the reconstruction result.

Changes made to the manuscript:

In addition, we constructed a prototype 1-m-long carbon dioxide gas cell. Following the alignment of two collimated fibers, the 2 μm ASE light source was coupled into the gas cell to acquire the absorption spectrum. Due to the lack of precise flow control in the carbon dioxide delivery system, the gas concentration fluctuated during the sampling process during the sampling process, leading to a degradation in reconstruction fidelity. The carbon dioxide absorption envelope remains clearly

discernible in the reconstructed spectrum in Fig. 5e.

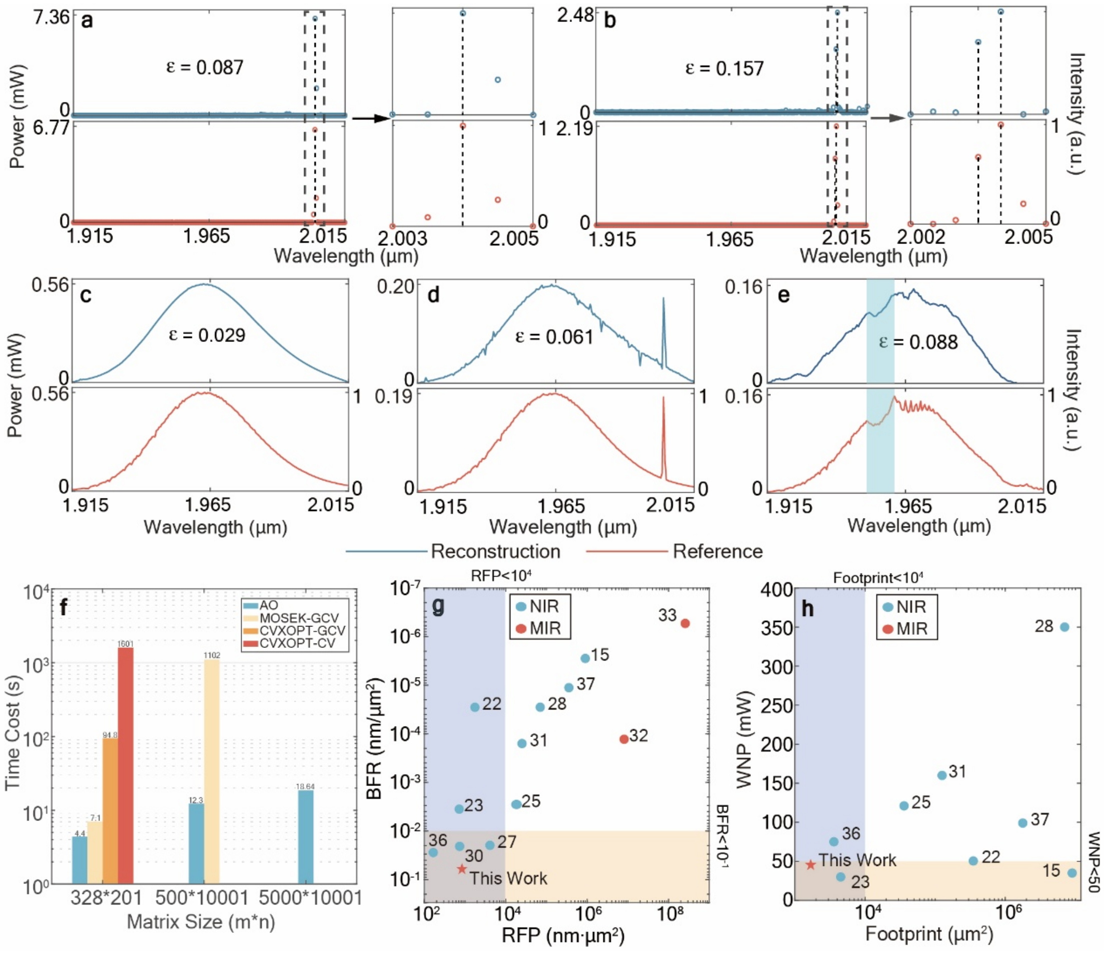

Fig. 5 | Reconstruction results and performance comparison. a-e The blue line is the algorithm reconstruction result, and the red line is the reference measured by the commercial spectrometer. **a** Single peak input spectrum and its reconstructed result. The peak is at 2004 nm. The right side shows an enlargement of the left side, which exhibits a full width at half maximum (FWHM) of 0.5 nm. **b** Dual peaks reconstructed result. An additional peak is set at 2003.5 nm adjacent to the 2004 nm peak. The right side displays a zoomed-in view of the left side. Compared to the **a**, the reconstruction result displays the additional peak line. **c** Broadband ASE input spectrum and its reconstructed result. **d** The input hybrid spectrum, which combines the ASE and a laser at 2004 nm. **e** Spectrum of ASE after absorption by carbon dioxide and its reconstruction result. The blue region represents the reconstructed absorption envelope. **f** Reconstruction efficiency of different methods for different matrix dimensions. For larger-scale problems, the reconstruction time of our alternating optimization does not increase significantly. Direct solution via convex optimization was implemented using CVXPY (solver: CVXOPT and MOSEK), while the alternating optimization method was carried out with the IRtools. Calculations were all performed on a laptop with a 10-core Apple M1 Pro CPU and 16 GB of RAM. AO: alternating optimization; MOSEK-GCV: convex optimization with MOSEK backend with GCV; CVXOPT-GCV: convex optimization with CVXOPT backend with GCV; CVXOPT-CV: convex optimization with CVXOPT backend with 10-fold-CV.The spectrometer performance is compared with the focus on **g** resolution-footprint product (RFP) and bandwidth-footprint ratio (BFR), **h** wavelength-normalized-power (WNP) and footprint.

Supplementary Note 11

11. Spectral Reconstruction of Lasers at Different Wavelengths

Since our tunable laser (OELTS-300) operates in the range of 1970–2070 nm, which does not fully cover the operating range of our spectrometer. Thus, we reconstructed the laser spectrum within the range of 1970–2015 nm. The reconstruction results are shown in Fig. **S18**.

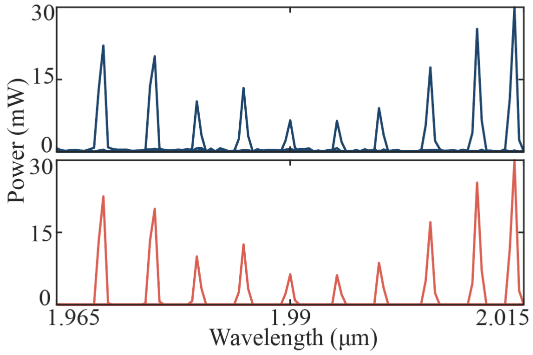

Fig. **S18**. Reconstructed spectrum of the tunable laser in the range of 1970–2015 nm. The red curve denotes the reference spectrum, and the blue curve represents the reconstruction result.

Q6: The term "benchmarking" in the title is misleading, as the study does not provide a systematic comparison against a range of existing MIR spectrometer technologies.

Reply: The authors sincerely appreciate the reviewer's professional insight regarding the use of the term "benchmarking." We agree that a systematic comparison across MIR is currently constrained by the relatively limited number of reported works in the on-chip MIR regime, most of which primarily utilize passive spectrometer architectures.

However, to provide a meaningful evaluation of our device's performance, we have expanded our comparative analysis to include several state-of-the-art NIR computational spectrometers. This cross-band comparison allows us to situate our work within the broader context of integrated spectrometer development. We have summarized the quantitative metrics (footprint, working band, resolution, bandwidth, channels, wavelength-normalized power, resolution-footprint product, and bandwidth-footprint ratio) in Supplementary Note 16.

Structure	Footprint (μm^2)	WB (μm)	RES (nm)	BW (nm)	CH	Active?	WNP (mW)	RFP ($nm \cdot \mu m^2$)	BFR ($nm \cdot \mu m^{-2}$)
Random disorder scattering[1]	1250	NIR (1.51)	0.75	25	25	N	/	5625	0.02
Stratified waveguide filters[2]	9100	NIR (1.55)	0.45	180	32	N	/	4095	0.0198
Cascaded nanobeam[3]	4500	NIR (1.55)	0.16	16	3	Y	30	720	3.6×10^{-3}
Grating-assisted F-P cavity[4]	3.6×10^{4}	NIR (1.50)	0.43	73.2	5	Y	121	1.84×10^{4}	2.9×10^{-3}
Photonic molecule[5]	3600	NIR (1.55)	0.04	100	1	Y	75	144	0.028
Programmable photonic circuits[6]	7.03×10^{6}	NIR (1.55)	0.01	200	1	Y	350	7.03×10^{4}	1.42×10^{-5}

**Digital FTS[7]**	1.78×10^{6}	NIR (1.55)	0.2	20	1	Y	99	3.55×10^{5}	1.13×10^{-5}
**Ring-assisted FTS[8]**	NA	NIR (1.60)	0.47	90	1	Y	1835	/	/
**High-Q Ring[9]**	3.5×10^{5}	NIR (1.55)	0.005	10	10	Y	50.4	1.75×10^{3}	2.86×10^{-5}
**Micro-disk[10]**	1.25×10^{5}	NIR (1.55)	0.2	20	1	Y	160	2.51×10^{4}	1.59×10^{-4}
**Arrayed Waveguide Grating and Ring[11]**	9×10^{6}	NIR (1.55)	0.1	25.4	9	Y	35	9×10^{5}	2.82×10^{-6}
**FTS Array[12]**	NA	MIR (3.30)	12	60	12	N	/	/	/
**FTS Array[13]**	9.5×10^{7}	MIR (3.75)	2.7	51	42	N	/	2.57×10^{8}	5.37×10^{-7}
**Waveguide Coupler[14]**	2.7×10^{6}	MIR (3.85)	3	350	1	Y	NA	8.1×10^{6}	1.29×10^{-4}
**MPCW (This work)**	**1680**	**MIR (2.00)**	**0.5**	**100**	**8**	**Y**	**45.6**	**840**	**0.06**

WB: waveband, RES: resolution, BW: bandwidth, CH: channel, WNP: wavelength-normalized power, RFP: resolution-footprint product, BFR: bandwidth-footprint ratio.

However, to avoid any potential misunderstanding and to more accurately reflect the core contributions of our work, we have revised the title. In light of the reviewer's insightful suggestions and the technical focus of this research, we have removed "benchmarking" from the title and revised it to:

"An efficient mid-infrared computational spectrometer based on synergistic microcavity-coupled photonic crystal waveguides"

We believe this revised title better captures the innovation of our hardware-software co-design and the resulting efficiency in spectral reconstruction.

Changes made to the manuscript:

Supplementary Note 16

16. Comparison of On-chip Spectrometers

Structure	Footprint (μm^2)	WB (μm)	RES (nm)	BW (nm)	CH	Active?	WNP (mW)	RFP ($nm\cdot\mu m^2$)	BFR ($nm\cdot\mu m^{-2}$)
**Random disorder scattering[1]**	1250	NIR (1.51)	0.75	25	25	N	/	5625	0.02
**Stratified waveguide filters[2]**	9100	NIR (1.55)	0.45	180	32	N	/	4095	0.0198
**Cascaded nanobeam[3]**	4500	NIR (1.55)	0.16	16	3	Y	30	720	3.6×10^{-3}

**Grating-assisted F-P cavity[4]**	3.6×10^{4}	NIR (1.50)	0.43	73.2	5	Y	121	1.84×10^{4}	2.9×10^{-3}
**Photonic molecule[5]**	3600	NIR (1.55)	0.04	100	1	Y	75	144	0.028
**Programmable photonic circuits[6]**	7.03×10^{6}	NIR (1.55)	0.01	200	1	Y	350	7.03×10^{4}	1.42×10^{-5}
**Digital FTS[7]**	1.78×10^{6}	NIR (1.55)	0.2	20	1	Y	99	3.55×10^{5}	1.13×10^{-5}
**Ring-assisted FTS[8]**	NA	NIR (1.60)	0.47	90	1	Y	1835	/	/
**High-Q Ring[9]**	3.5×10^{5}	NIR (1.55)	0.005	10	10	Y	50.4	1.75×10^{3}	2.86×10^{-5}
**Micro-disk[10]**	1.25×10^{5}	NIR (1.55)	0.2	20	1	Y	160	2.51×10^{4}	1.59×10^{-4}
**Arrayed Waveguide Grating and Ring[11]**	9×10^{6}	NIR (1.55)	0.1	25.4	9	Y	35	9×10^{5}	2.82×10^{-6}
**FTS Array[12]**	NA	MIR (3.30)	12	60	12	N	/	/	/
**FTS Array[13]**	9.5×10^{7}	MIR (3.75)	2.7	51	42	N	/	2.57×10^{8}	5.37×10^{-7}
**Waveguide Coupler[14]**	2.7×10^{6}	MIR (3.85)	3	350	1	Y	NA	8.1×10^{6}	1.29×10^{-4}
**MPCW (This work)**	**1680**	**MIR (2.00)**	**0.5**	**100**	**8**	**Y**	**45.6**	**840**	**0.06**

WB: waveband, RES: resolution, BW: bandwidth, CH: channel, WNP: wavelength-normalized power, RFP: resolution-footprint product, BFR: bandwidth-footprint ratio.

The wavelength-normalized power (WNP) is defined as:

$$\mathrm{WNP} = P_{\mathrm{working}} \times \frac{P_{\pi}(1550)}{P_{\pi}(\text{center wavelength})}$$

Benchmarking Mid-Infrared On-Chip Spectrometer with Microcavity-Coupled Photonic Crystal Waveguides

Reviewer comments

The mid-infrared (MIR) waveband contains a vast number of vibrational absorption signatures for a range of applications. MIR spectroscopy is an important technique to identify these signatures. However, there is a growing demand to shrink the existing bulky and expensive systems (i.e. FTIR / otherwise). Integrated photonic approaches to realise miniaturized MIR spectroscopy are promising, but numerous roadblocks exist including materials transparency limitations, sensitivity (sensing architectures), optical throughputs, necessity for long optical path lengths (i.e. mimicking FTIR), narrow bandwidths etc. etc. In this work, the authors present quite a novel miniaturized MIR computational spectrometer prototype based on microcavity-coupled photonic crystal waveguide (MPCW) architecture. The metrics are a 100 nm bandwidth with 0.5 nm resolution (at approx. 2 um), spectrum acquisition of 4 seconds and footprint. This is achieved using an array of tapered PCWs, thermal tuning and computational reconstruction techniques. The architecture is novel, and performance is impressive compared to the state-of-the-art in on-chip spectrometers. Comparison to commercial systems is lacking, calibration and stability requirements are demanding, susceptibility to error is large, bandwidth is small (and debatable as to how scalable this is) and there are other numerous drawbacks (mentioned below). **Nonetheless, I would recommend to accept the work after addressing the changes / suggestions below. The points and questions I would like to addressed are as follows, and are listed in no particular order:**

- Authors state: “scalability of our MPCWs enables easy extension of the operational range to other wavelength bands”. Si-SiO2 is opaque beyond approx 4um (specifically the SiO2), therefore how is it expected to scale this to longer wavelengths? Are you fundamentally limited by your design to shorter wavebands? Also, to evidence this, the authors (in Fig S.2), have a band edge wavelength v lattice constant plot, however this doesn’t indicate the power loss associated with moving towards 3.5 um due to the increased extinction of the materials.
    - Further, on the topic of scalability: how would this system be practically translated to real world applications? As in it’s current form it is a world away from realization in any demanding environment.
- Bandwidth limitations: is there still a fundamental limitation to scaling beyond the 100 nm bandwidth presented? If so, this would mean again spectrometers for specific gases / analytes and not a broadband spectrometer (i.e. FTIR)
- The title of the paper and the SI are different.
    - The title of this paper should not have the word benchmarking in, as it doesn’t make sense here. Also, please clarify the actual paper title.
- Sensitivity and optical throughputs: typically, coupling into integrated photonic devices, then in-plane losses (splitters, etc result in very low throughputs and thus combined with fabrication errors etc. I would suspect the sensitivity of this device is low. Can you provide a quantitative measure please? The impact on the performance or detection limits is not discussed.
    - Further, any slow-light device (or PCW device) is highly susceptible to fabrication tolerances (ie. Resonant linewifth / band edge deviations) and even small deviations degrade performance significantly. Do you have simulation / otherwise results to indicate the tolerances and how you could get around them? i.e. computationally / otherwise.

- Cavity design choice: could the authors please discuss why the cavity design was chosen? Was this informed from simulations not shown in the paper? For example, one could imagine using non-uniform cavity designs which may offer ‘better’ performance.
- Thermal tuning and crosstalk: there must be some coupling between channels, especially if this is operated repeatedly. Does this result in performance degradation with time or longer term thermal drift?
- Clarification of “…due to the limitations of hardware sampling and computational resources, obtaining a continuous spectrum is impractical.”
- Computational reconstruction: The combined L1-L2 based reconstruction approach: These approaches are quite susceptible to noise and the actual reconstruction quality (i.e. some types of signals are poorly reconstructed).
    - Can you please quantify noise metrics and reconstruction quality to ground truth spectra of additional signal types (not just a laser or ASE), something more akin to sensing (molecular analyte)? I’m concerned that the chosen inputs are selected due to the reconstruction ‘ease’ / architecture being poor at reconstructing say more natural signals or more complex signals. Please alleviate my concerns. Also, normally one enforces the idea of sparsity or smooth signals, and thus real-world spectra may not ‘adhere’ to these constraints.
- Figure 4e (and data). Thermally tuned transmission spectra: The variation in the spectral function as a function of temperature (power) is quite minimal. For computational reconstruction, maximal variation in the encoder functions is normally preferred, therefore why is this PCW structure chosen (ie ordered) and not something more random / quasi-random? How can you increase the variation of the spectra rather than just band edge spectral shift?
- Subspeciality to error: How susceptible is your architecture to sources and magnitude of error? There is no mention of matrix condition number or reconstruction error bounds.
    - Why is 7% random noise chosen? Also, could you please indicate actual power too rather than normalized power (i.e. in Fig 3e)? this can be in the text if needed. Building on this, it is normal to include an idea of minimum noise floor for spectrometer systems (dark noise conventionally), what is this for this system?
- Figure 3b and 3c need scale bars. 3b needs identification of what each part of the micrograph is. Fig 4c needs a scale bar and identification of what each part is.
- The thermally tuned architecture appears relatively susceptible to ambient (or unwanted local) temperature fluctuations. The authors state the packaged chip is integrated with a TEC, however this is power hungry and bulky, is this a limitation for this approach? For example, even if the PIC is small, the cooler + MEMS optical switch + other elements etc. will always limit the system.
- Fig 5 f g, can you please indicate what the shaded blue and yellow regions represent.
- Throughout the paper (especially the figures), normalized power / transmission is used. It’s unclear as to what these are normalized against (e.g. themselves, or some other source, etc. etc.). When comparing to other approaches (which are normally higher optical throughput / efficiency) it would be beneficial to know the actual numerical values.
    - Further, what would be useful is to know whether the 4 seconds of reconstruction is down to the need to ‘collect more light’ or the reconstruction algorithm. Thus, could you include a plot of potentially reconstruction accuracy

as a function of time? As for example, there are some applications which require highly accurate spectra and some which only require the knowledge of the presence of some signal rather than complete reconstruction,

- Again, the idea of optical throughput (or efficiency), the authors state: “the system is readily scalable by adding more MPCW channels with varied lattice constants”, which is true, but this then divides the same optical power (which is low anyway) to more separate channels which is inherently more lossy. Therefore, how does this power scale? For example, does 1 more channel result in increased reconstruction time? Less reconstruction time but the tradeoff is power loss? If so, but how much and what is the balance here?
- I couldn’t see any mention of polarization sensitivity but assume this is a critical parameter too – could you briefly indicate how the polarization control is maintained?
- MEMS FTIR [Hamamatsu light engine] comparison: I can currently buy a small MEMS FTIR which covers 1.1 to 2.5 um, with spectral resolution down to 5 nm (but can be adjusted). The cost will be cheaper, throughput higher, bandwidth much larger etc. And when comparing total package volume and weight, I suspect not too dissimilar to the PIC approach (when combined with coupling optics, TEC etc.). Therefore, can you please compare and contrast to actual commercial spectrometers? Because the state of the art is not just what researchers are doing but what one can actually buy and I’d like to know the potential of the architecture here compared to what’s commercially available and what need your approach is satisfying.
